# Synthesis and ring openings of cinnamate-derived N-unfunctionalised aziridines

**DOI:** 10.3762/bjoc.8.199

**Published:** 2012-10-12

**Authors:** Alan Armstrong, Alexandra Ferguson

**Affiliations:** 1 Department of Chemistry, Imperial College London, South Kensington, London, SW7 2AZ, U.K.

**Keywords:** amino acids, aziridination, organocatalysis, ring opening, stereoselectivity

## Abstract

*tert*-Butyl cinnamates are aziridinated with high trans-selectivity by an N–N ylide generated in situ from *N-*methylmorpholine and *O-*diphenylphosphinyl hydroxylamine. The resulting N*-*unfunctionalised aziridines are shown to be versatile synthetic building blocks that undergo highly selective ring-opening reactions with a wide range of nucleophiles.

## Introduction

**Scheme 1:**
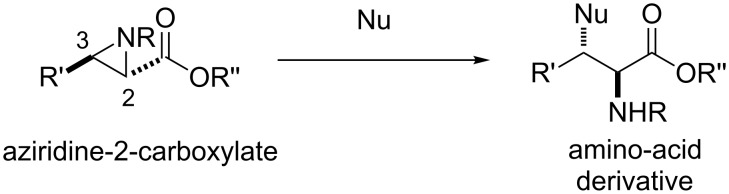
Regioselective ring opening of an aziridine-2-carboxylate.

Amino acids are the fundamental building blocks of life on earth, and consequently their molecular structure is ubiquitous in natural and synthetic chemistry [1]. An attractive method of installing the amino-acid functionality is through the regioselective ring opening of an aziridine-2-carboxylate (Scheme 1) [2–3].

Such a methodology has been developed for N*-*functionalised aziridines [4–9], as N*-*functionality offers both activation of the aziridine to ring opening [10], and protection of the nitrogen. However, an extra synthetic step may be required to remove the N-functionality, which is often difficult and low yielding [11–15]. Ring opening of N*-*unfunctionalised (*NH*-) aziridines allows direct access to amino-acid derivatives, yet the published methodology is currently limited for this transformation [16–20]. In literature examples where ring-opening protocols have been developed, synthesis of the starting *NH-*aziridine-2-carboxylates is multistep and low-yielding. Synthetic routes include Gabriel–Cromwell addition of ammonia to an enoate [16–17]; epoxide opening with azide, followed by ring closure [19]; and a sequence of olefin dihydroxylation, conversion to a cyclic sulfate, ring opening with azide, and finally ring closure to afford the *NH*-aziridine [18].

**Figure 1:**
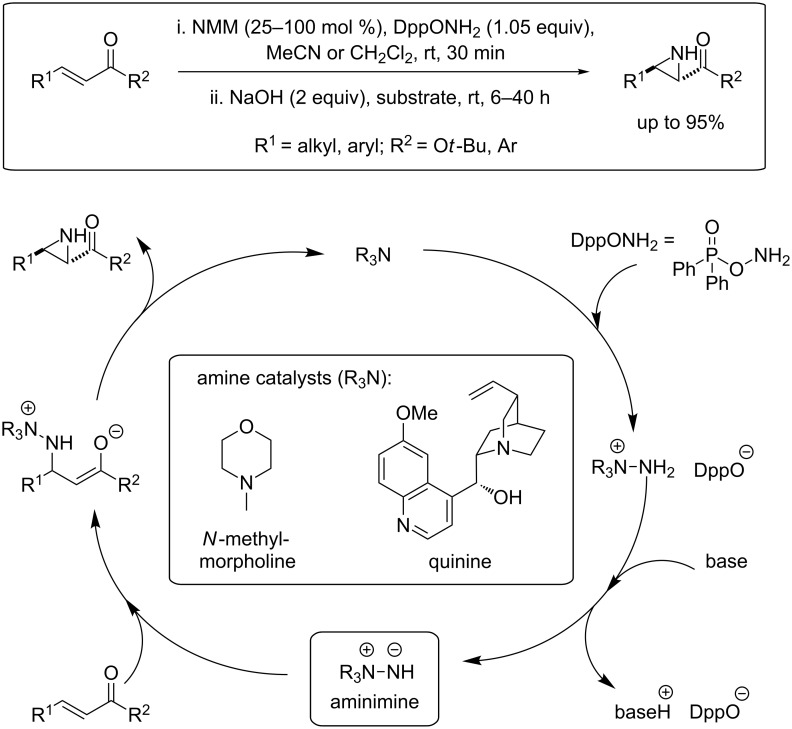
Amine-promoted alkene aziridination.

We recently reported [21–22] a nucleophilic aziridination methodology [23–30] that allows access to *NH*-aziridines in a single step from α,β-unsaturated carbonyl compounds. Our method produces *trans*-*NH*-aziridines from α,β-unsaturated carbonyl compounds in good to excellent yield with high diastereoselectivity (Figure 1) [21]. An N–N ylide (aminimine) is the presumed active intermediate, formed from the reaction of a tertiary amine promoter and an aminating agent. Conjugate addition of the ylide, followed by a ring-closure step affords the *NH*-aziridine. More recently, the methodology has been extended to α,β,γ,δ-unsaturated carbonyl compounds, with excellent regio- and diastereoselectivity [31].

Herein, we report the optimisation of our nucleophilic aziridination methodology to access *NH*-aziridine-2-carboxylates in a single step from enoates. To the best of our knowledge, there exist only three examples of this transformation. Furukawa used diphenyl sulfilimine to form *NH*-aziridines from fumarate esters [32], and *O*-mesitylenesulfonylhydroxylamine (MSH) was used to aziridinate electron-poor alkenes by Hamelin [33]. We described the aziridination of two *tert*-butyl enoate substrates in our initial disclosure, but arguably the more synthetically useful of these, *tert*-butyl cinnamate, gave a modest yield of 32% [21]. We also report here our studies into the ring opening of aziridine **1a**, derived from *tert-*butyl cinnamate. As a result, a diverse array of amino-acid derivatives is generated in three steps from commercially available starting materials.

## Results and Discussion

The aziridination of *tert*-butyl cinnamate was the starting point chosen for our optimisation. A control reaction in the absence of NMM gave no aziridine product. Despite the low conversion in the reported procedure [21], the only compounds apparent in the ^1^H NMR spectrum of the crude reaction mixture were the starting cinnamate and the aziridine. A time delay of 20 minutes between the addition of NaOH and the cinnamate to the reaction mixture led to increased conversion (Table 1, entry 1 versus entry 2), with longer times leading to lower conversions (Table 1, entry 3 and entry 4). Using 4 equiv of NaOH increased the conversion to 50% (Table 1, entry 5), although a large excess (20 equiv) did not increase the conversion any further (Table 1, entry 5 versus entry 6). Increasing the concentration of the reaction mixture was found to have the most significant effect; performing the reaction at a concentration of 0.24 M provided a 63% conversion to aziridine (Table 1, entry 8). Problems with mechanical stirring were observed at concentrations above 0.24 M, due to the heterogeneous nature of the reaction mixture. The isolated yield of this reaction (51%) was slightly lower than the conversion, but was reproducible on a 12 mmol scale (Table 1, entry 9). Use of an increased number of equiv of the aminating agent, tertiary amine and NaOH provided the aziridine in 65% isolated yield on a 3 mmol scale (Table 1, entry 10). The increased equiv of the heterogeneous reagents meant that the concentration of the reaction was reduced to 0.12 M for these conditions. The reaction was found to be specific for *tert*-butyl esters, with γ- and δ-benzylidene lactones, methyl, ethyl, phenyl, and benzyl cinnamates all suffering from degradation, presumably via hydrolysis pathways. β-Alkyl-substituted enoates were not explored, and will be the subject of future investigation. A preliminary screen of alternative solvents, tertiary amines and bases showed that NMM and NaOH in CH_2_Cl_2_ was the optimum reagent combination for the aziridination.

**Table 1:** Enoate aziridination optimisation.^a^

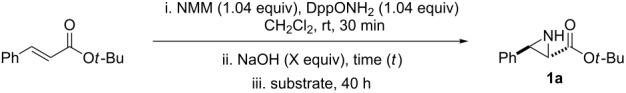				

Entry	NaOH (equiv)	Concentration^b^ (M)	Time for step ii (min)	Conversion (%)^c,d^

1	2	0.06	0	32 (30)
2	2	0.06	20	47
3	2	0.06	30	41
4	2	0.06	160	23
5	4	0.06	20	50
6	20	0.06	20	47
7	4	0.12	20	58
8	4	0.24	20	63 (51)
9^e^	4	0.24	20	63 (50)
10^f^	11	0.12	20	(65)

^a^All reactions performed on 0.11 mmol scale unless stated otherwise. ^b^With respect to *tert-*butyl cinnamate. ^c^ Estimated by ^1^H NMR: relative integration of *tert-*butyl peaks in starting material and product. ^d^Isolated yield in parentheses. ^e^12 mmol scale. ^f^NMM (3 equiv), DppONH_2_ (3 equiv), 3 mmol scale.

Having optimised our aziridination protocol for *tert-*butyl cinnamate, our attention then turned to examining the scope for aromatic substitution in the aziridination. A range of *ortho-, meta-* and *para-*substituted cinnamates were synthesised via Wittig, Heck or esterification routes (for details, see Supporting Information File 1). Each substrate was subjected to two sets of aziridination conditions, with the optimum yields reported.

Electron-rich substrates and the *ortho-*chloro cinnamate favoured conditions (B), in which increased equiv of reagents were used (Table 2, entries 2, 3, 7, 9). Electron-poor substrates and *para-*halo-substituted cinnamates provided optimum yields when subjected to conditions (A), using fewer equiv of reagents (Table 2, entries 4–6, 8, 10). Poor isolated yields were observed when conditions (B) were used on electron-poor substrates; this was attributed to the increased susceptibility to hydrolysis of the aziridine products. The increased steric demand posed by *ortho* substitution was found to have a detrimental effect on the aziridination (Table 2, entry 6 versus entry 8). A 5-chloro-furyl-substituted enoate was also subjected to the aziridination (Table 2, entry 10); a shorter reaction time of 20 h under the moderate set of aminating conditions (A) was optimal for this substrate. In all cases, only starting material and product were observed in the ^1^H NMR spectrum of the crude reaction mixture. However, a loss in mass recovery was often observed during the isolation/purification process (see Table 2, isolated yield and recovered starting material columns), meaning that many of the isolated yields of aziridine are moderate. Nonetheless, it is worth noting that this single-step transformation provides *NH*-aziridine-2-carboxylates in competitive or superior yields to alternative multistep methodologies.

**Table 2:** Substrate scope of enoate aziridination.

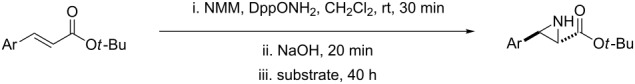					

Entry		Ar	Conditions^a^	Isolated yield (%)	R.S.M^b^ (%)

1^c^	**1a**	Ph	B	65	6
2	**1b**	(4-OMe)C_6_H_4_	B	39	34
3	**1c**	(4-Me)C_6_H_4_	B	36	12
4^d^	**1d**	(4-I)C_6_H_4_	A	33	40
5^e^	**1e**	(4-Cl)C_6_H_4_	A	53	12
6	**1f**	(4-NO_2_)C_6_H_4_	A	76	6
7	**1g**	(2-Cl)C_6_H_4_	B	47	5
8	**1h**	(2-NO_2_)C_6_H_4_	A	40	24
9	**1i**	(3-OMe)C_6_H_4_	B	32	14
10	**1j**	(5-Cl)furyl	A^f^	35	44

^a^Conditions A: NMM (1.04 equiv), DppONH_2_ (1.04 equiv), NaOH (4 equiv), 0.24 M; Conditions B: NMM (3 equiv), DppONH_2_ (3 equiv), NaOH (11 equiv), 0.12 M. All reactions performed on 0.20–0.23 mmol scale. ^b^Recovered starting material (cinnamate ester) following purification. ^c^2.90 mmol scale. ^d^0.15 mmol scale at 0.08 M. ^e^Reaction performed at 0.06 M. ^f^20 h reaction.

Having explored the scope of the aziridination, we were keen to investigate the susceptibility of our unactivated *NH*-aziridines to ring-opening chemistry. To the best of our knowledge, only four papers exist detailing the regioselective ring opening of *NH-*aziridine-2-carboxylates to provide amino-acid derivatives [16–19]. The substrate scope has been limited to *NH-*aziridine-2-carboxylates derived from isopropyl [16], menthyl [17], benzyl [18], and methyl [19] cinnamates. Nucleophiles are limited to thiophenols [17–19], fluoride [16], indole [18–19] and acetic acid [18–19] and a hydrogenation protocol also exists [18]. We were keen to test these nucleophiles in the ring opening of our *NH-*aziridines, in order to provide products with alternative ester functionality in the form of a *tert*-butyl group. Additionally, we wanted to expand the range of ring-opening nucleophiles, to provide access to a wider variety of amino-acid derivatives.

**Figure 2:**
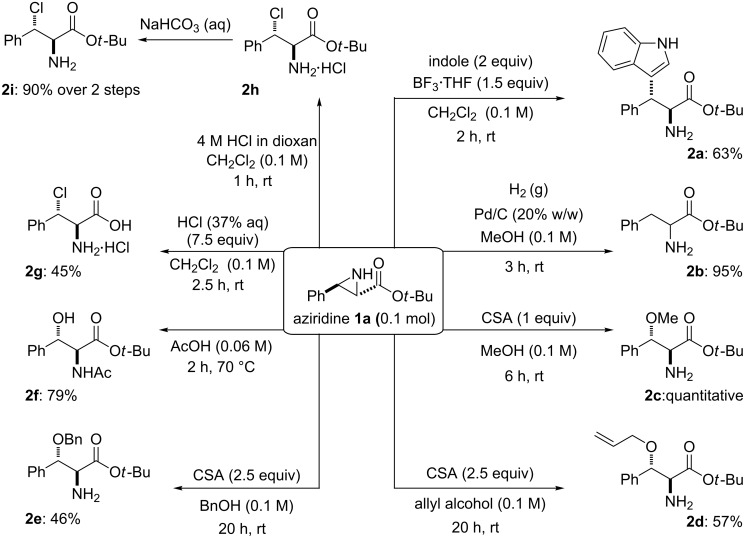
Ring-opened products of aziridine **1a**.

Aziridine **1a** was selected for initial investigations. Pleasingly, aziridine **1a** was found to undergo ring opening under Lewis and Brønsted acid catalysed conditions with a range of nucleophiles to provide amino-acid derivatives in good to excellent yield (Figure 2). The ring openings proved to be completely regioselective for the C3-benzylic position of aziridine **1a**. This regioselectivity was confirmed through atom correlations observed in 2D NMR experiments and by comparison with literature compounds, including C2-substituted analogues.

Indole was the first nucleophile investigated, as regioselective ring opening of the analogous benzyl [18] and methyl [19] ester protected *NH*-aziridines with indole in the presence of BF_3_·OEt_2_ had previously been reported, in 48 and 53% yields, respectively. On subjecting the orthogonally protected aziridine **1a** to the reported conditions, only a 7% isolated yield of ring-opened product was observed. Pleasingly, the use of BF_3_·THF resulted in a considerably cleaner reaction. Separation of a major and minor product was achieved, with the major product identified as the tryptophan derivative **2a**, isolated in 63% yield. The regiochemistry of **2a** was established through correlation spectroscopy. A ^2^*J* correlation between the *ipso*-carbons of both aromatic systems and the C3 proton was observed by HMBC NMR. Comparison of the ^1^H NMR chemical shifts of the minor product with **2a** and literature compounds led to the tentative assignment of the minor product as the C3 ring-opened diastereomer of **2a**. This reaction provides an attractive example of how our ring-opening chemistry can be used to form new carbon–carbon bonds.

Hruby reported the hydrogenation of the benzyl ester analogue of aziridine **1a**, resulting in the concomitant removal of the benzyl group to provide free phenylalanine [18]. Pleasingly, treatment of aziridine **1a** with H_2_ in the presence of Pd/C provides phenylalanine *tert-*butyl ester (**2b**) in an excellent yield of 95%, without the need for purification. The orthogonal protection provided by the *tert*-butyl ester yields a product that is easier to handle than the free amino acid.

The ring opening of *NH*-aziridine-2-carboxylates with alcoholic nucleophiles is almost unexplored. To the best of our knowledge, only one literature example exists, in which the *NH-*aziridine derived from dimethyl fumarate is subjected to ring opening with MeOH in 50% yield [20]. We report that the ring opening of aziridine **1a** with alcohols in the presence of camphorsulfonic acid (CSA), provides a facile synthesis of 1,2-aminoalcohols. The alcohol nucleophiles were used as solvents, as stoichiometric quantities resulted in decreased conversions. Single ring-opened products were observed in the ^1^H NMR spectra of the crude products in all cases. In MeOH, amino-acid derivative **2c** was obtained in quantitative yield, without the need for purification. This represents the first regioselective example of the ring opening of an *NH-*aziridine-2-carboxylate with methanol. Allyl alcohol gave access to amino-acid derivative **2d** (57%). The terminal olefin provides a useful handle for further functionalisation of this substrate. Reaction of benzyl alcohol yielded the β-benzyloxy amino-acid derivative **2e** in 46%. The orthogonal benzyl protection could allow the free alcohol to be accessed selectively by hydrogenation protocols, leaving the *tert-*butyl ester intact. Products **2d** and **2e** are the first examples of the ring opening of *NH-*aziridine-2-carboxylates with alcoholic nucleophiles other than MeOH.

Acetic acid has previously been used as a nucleophile to achieve ring opening of benzyl [18] and methyl-ester [19] substituted *NH-*aziridines in 88% and 69% yields, respectively, with *O-* to *N-*acetate transfer observed. Ring opening of **1a** in acetic acid at 70 °C provides pure product **2f** (79%), with orthogonal *tert-*butyl ester protection in contrast to the reported examples. As detailed in the literature, the acetate protecting group was found to transfer to the nitrogen of the ring-opened aziridine. Product **2f** can be considered complementary to **2e**; O- or N-protected amino-acid derivatives can be accessed easily, depending on the choice of conditions.

Finally, β-chlorophenylalanine derivatives were accessed in three different forms, depending on the choice of HCl source and work-up conditions employed. Treatment of aziridine **1a** with HCl (35% aq) in dichloromethane led to isolation of **2g**, i.e., the HCl salt of β-chlorophenylalanine, in 45% yield. It is notable that this is the only one of our examples in which the *tert*-butyl ester is cleaved to provide the amino-acid motif. Altering the HCl source to 4 M HCl in dioxane provided product **2h** as the HCl salt, with the *tert*-butyl ester intact. Treatment with aqueous NaHCO_3_ provided pure β-chloroamine **2i** in 90% yield over two steps. The chlorine atom provides a useful handle for further functionalisation, via substitution or coupling reactions, for example [34–35]. This is the first reported regio- and diastereoselective synthesis of *NH-*β-chlorophenylalanine derivatives from *NH-*aziridine-2-carboxylates.

## Conclusion

In summary, we have reported the synthesis of a range of amino-acid derivatives from the ring opening of an *NH-*aziridine in excellent regio- and diastereoselectivity. *NH-*aziridines were synthesised in a single step from *tert*-butyl cinnamates, following optimisation of our nucleophilic aziridination methodology. This allows access to amino-acid derivatives in three steps from commercially available starting materials. Efforts are currently underway to develop an enantioselective version of the aziridination that is applicable to *tert*-butyl enoates.

## Supporting Information

File 1 Experimental procedures and characterisation data for all new *NH-*aziridines, including ^1^H and ^13^C NMR spectra for all new compounds.
