# Supplementary material for: Synthesis and ring openings of cinnamate-derived N-unfunctionalised aziridines
Source: Beilstein J Org Chem. 2012 Oct 12;8:1747–52. doi: 10.3762/bjoc.8.199 (PMC3511008; doi:10.3762/bjoc.8.199)
Supplement: File 1 — Experimental procedures and characterisation data for all new NH-aziridines, including 1H and 13C NMR spectra for all new compounds. [file Beilstein_J_Org_Chem-08-1747-s001.pdf]

Supporting Information

for

Synthesis and ring openings of cinnamate-derived N-unfunctionalised aziridines

Alan Armstrong*, Alexandra Ferguson

Address: Department of Chemistry, Imperial College London, South Kensington, London, SW7 2AZ, U.K.

Email: Alan Armstrong* - A.Armstrong@imperial.ac.uk

*Corresponding author

Experimental procedures and characterisation data for all new *NH*-aziridines, including ^{1}H and ^{13}C NMR spectra for all new compounds

Contents

S2 General experimental
S2 Experimental details
S2 Synthesis of enoates **3a**–**j**
S6 Synthesis of aziridines **1a**–**j**
S11 Ring-opening products **2a**–**i**
S16 NMR spectra of compounds **3a**, **1b**–**j**, **2a**–**i**
S53 Crystal structure of aziridine **1a**
S54 References

General experimental

Methylene chloride, toluene, methanol and THF were purified by passage through an alumina column before use. Indole was purified by passage through a silica column and stored in a desiccator. $DppONH_2$ was prepared and stored in a desiccator. NaOH was ground to a fine powder immediately prior to use. All other commercial reagents were used as supplied. Reactions were run under a positive pressure of argon in oven- or flame-dried glassware with magnetic stirring. Reaction temperatures were recorded as bath temperatures. Flash column chromatography was carried out by using silica gel, particle size 40–60 µm. Analytical thin layer chromatography (TLC) was performed by using glass-backed plates precoated with silica gel 60 F254. Melting points were obtained using a hotplate microscope and are uncorrected. Infrared analyses were recorded using ATR. NMR analyses were recorded at 400 or 500 MHz in $CDCl_3$ or MeOD-d_4 as specified. Chemical shifts are quoted in ppm relative to TMS (as referenced to the residual solvent, e.g., $CHCl_3$ δH 7.26 or $CDCl_3$ δC 77.0), with coupling constants quoted in hertz and reported to the nearest 0.1 Hz. ^{13}C assignments, when given, are based on DEPT 135 experiments. Mass spectrometry analyses were carried out by using CI^+ (NH_3), ES^+ or EI.

Experimental details

Synthesis of enoates 3a–j

General procedure A:

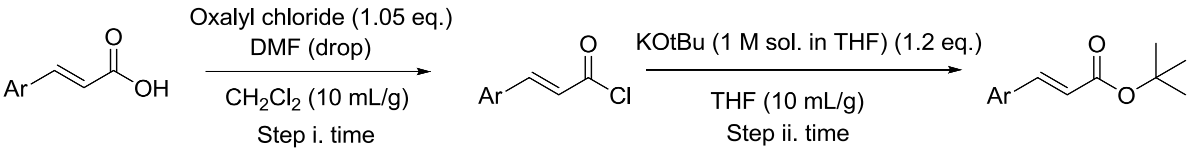

Oxalyl chloride (1.05 equiv) was added dropwise to the required cinnamic acid (1 equiv) in CH_2Cl_2 (10 mL/g), followed by a drop of DMF. Once all the starting acid had been consumed (TLC analysis of an aliquot after a MeOH quench), the solvent was removed *in vacuo*. THF (10 mL/g) was added and the mixture cooled to 0 °C. KO*t*-Bu (1M solution in THF, 1.2 equiv) was added. After 10 min at 0 °C, the ice bath was removed and the reaction mixture stirred at rt Once TLC analysis showed consumption of starting acid chloride, the reaction was diluted with diethyl ether (30 mL/g) and washed with water (30 mL/g). The aqueous phase was extracted with diethyl ether (3 × 30 mL/g), and the combined organic phases were dried over $MgSO_4$ and concentrated *in vacuo*.

(*E*)-*tert*-Butyl cinnamate (3b)

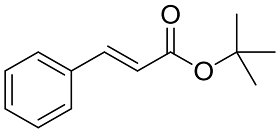

Following general procedure A; time for step i.: 2 h.; time for step ii.: 16 h. 3.62 g of cinnamic acid provided the title compound as a yellow oil (4.49 g, 90%) with no need for purification. R_f 0.35 (UV 254 nm, petroleum ether/EtOAc, 20:1); δ_H ($CDCl_3$, 400 MHz) 7.60 (1H, d, *J* 16.0, PhC**H**), 7.56–7.52 (2H, Ar**H**), 7.43–7.38 (3H, Ar**H**), 6.42 (1H, d, *J* 16.0, *t*-BuOC(O)C**H**), 1.55 (9H, s, C(C**H₃**)₃). All data are in agreement with literature values [1].

(*E*)-*tert*-Butyl 4-methoxycinnamate (3c)

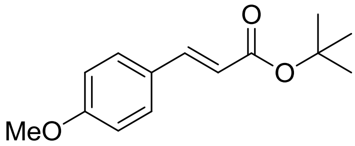

Following general procedure A; time for step i.: 30 min; time for step ii.: 16 h. 0.50 g of 4-methoxycinnamic acid provided the title compound as a white powdery solid (0.43 g, 65%), after purification by column chromatography on silica: petroleum ether/EtOAc (20:1). Mp 43–44 °C [lit: 41–44 °C]; R_f 0.30 (UV 254 nm, petroleum ether/EtOAc, 20:1); δ_H ($CDCl_3$, 400 MHz) 7.55 (1H, d, *J* 16.0, ArC**H**), 7.47 (2H, dt, *J* 8.8 and 2.0, OCH_3CCHC**H**), 6.91 (2H, dt, *J* 8.8 and 2.0, OCH_3CC**H**), 6.25 (1H, d, *J* 16.0, *t*-BuOC(O)C**H**), 3.86 (3H, s, OC$\mathbf{H_3}$), 1.55 (9H, s, C(C$\mathbf{H_3}$)$_3$). All data are in agreement with literature values [2].

(*E*)-*tert*-Butyl 4-nitrocinnamate (3d)

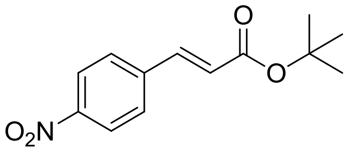

Following general procedure A; time for step i.: 50 min; time for step ii.: 4 h. 0.50 g of 4-nitrocinnamic acid provided the title compound as a brown solid (0.41 g, 63%), with no need for purification. Mp 152–55 °C [lit:155–57 °C]; R_f 0.25 (UV 254 nm, petroleum ether/EtOAc, 10:1); δ_H ($CDCl_3$, 400 MHz) 8.26 (2H, d, *J* 8.4, NO_2CC**H**), 7.67 (2H, d, *J* 8.4, NO_2CCHC**H**), 7.62 (1H, d, *J* 16.0, ArC**H**), 6.50 (1H, d, *J* 16.0, *t*-BuOC(O)C**H**), 1.56 (9H, s, C(C$\mathbf{H_3}$)$_3$). All data are in agreement with literature values [2].

(*E*)-*tert*-Butyl 4-methylcinnamate (3e)

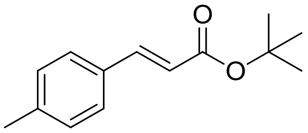

Following general procedure A; time for step i.: 50 min; time for step ii.: 4 h. 0.50 g of 4-methylcinnamic acid provided the title compound as a brown oil (0.53 g, 79%), with no need for purification. R_f 0.35 (UV 254 nm, petroleum ether/EtOAc, 10:1); δ_H ($CDCl_3$, 400 MHz) 7.57 (1H, d, *J* 16.0, ArC**H**), 7.42 (2H, d, *J* 8.0, CH_3CCHC**H**), 7.19 (2H, d, *J* 8.0, CH_3CC**H**), 6.33 (1H, d, *J* 16.0, *t*-BuOC(O)C**H**), 2.39 (3H, s, ArC**H₃**), 1.56 (9H, s, C(C**H₃**)₃). All data are in agreement with literature values [2].

(*E*)-*tert*-Butyl 4-chlorocinnamate (3f)

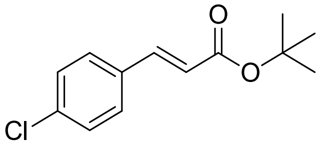

Following general procedure A; time for step i.: 1 h.; time for step ii.: 16 h. 0.50 g of 4-chlorocinnamic acid provided the title compound as a white solid (0.55 g, 84%), after purification by column chromatography on silica: petroleum ether/EtOAc (20:1). Mp 70–72 °C [lit:69–71 °C]; R_f 0.35 (UV 254 nm, petroleum ether/EtOAc, 20:1); δ_H ($CDCl_3$, 400 MHz) 7.54 (1H, d, *J* 15.6, ArC**H**), 7.46 (2H, dt, *J* 8.8 and 2.0, ClCC**H**), 7.36 (2H, dt, *J* 8.8 and 2.0, ClCCHC**H**), 6.35 (1H, d, *J* 15.6, *t*-BuOC(O)C**H**), 1.56 (9H, s, C(C**H₃**)₃). All data are in agreement with literature values [2].

(*E*)-*tert*-Butyl 3-methoxycinnamate (3g)

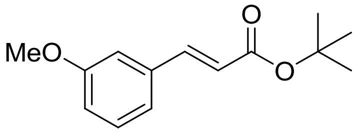

Following general procedure A; time for step i.: 1 h 30 min; time for step ii.: 4 h. 1.00 g of 3-methoxycinnamic acid provided the title compound as a yellow oil (1.32 g, quantitative yield), with no need for purification. R_f 0.40 (UV 254 nm, petroleum ether/EtOAc, 20:1); δH ($CDCl_3$, 400 MHz) 7.57 (1H, d, *J* 16.1, ArC**H**), 7.30 (1H, t, *J* 8.1, Ar**H**), 7.11 (1H, d, *J* 7.4, Ar**H**), 7.03 (1H, br t, *J* 2.4, Ar**H**), 6.93 (1H, dd, *J* 8.1 and 2.4, Ar**H**), 6.37 (1H, d, *J* 16.1, C(O)C**H**), 3.82 (3H, s, OC**H₃**), 1.54 (9H, s, C(C**H₃**)₃). All data are in agreement with literature values [3].

(*E*)-*tert*-Butyl 2-nitrocinnamate (3h)

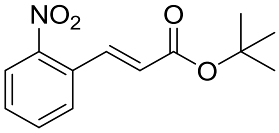

Following general procedure A; time for step i.: 2 h. then a further portion of oxalyl chloride (1.05 equiv) added, then 50 min; time for step ii.: 16 h. 1.00 g of 2-nitrocinnamic acid provided the title compound as a yellow oil (0.17 g, 13%), after purification twice by column chromatography on silica: petroleum ether/EtOAc (5:1), then petroleum ether/EtOAc (5:1). R_f 0.40 (UV 254 nm, petroleum ether/EtOAc, 5:1); ν_{max}/cm^{-1} 2990, 1736, 1704, 1524, 1332, 1145; δH ($CDCl_3$, 400 MHz) 8.01 (1H, d *J* 15.6, ArC**H**), 8.01 (1H, d *J* 7.6, Ar**H**), 7.64 (2H, Ar**H**), 7.55 (1H, m, Ar**H**), 6.32 (1H, d, *J* 15.6, ArCHC**H**), 1.53 (9H, s, C(C**H$_3$**)$_3$); δC ($CDCl_3$, 101 MHz) 165.0, 148.3, 138.7, 133.4, 130.7, 130.1, 129.1, 125.2, 124.8, 81.1, 28.1. All data are in agreement with literature values [4].

(*E*)-*tert*-Butyl 3-(5-chlorofuran-2-yl)propenoate (3a)

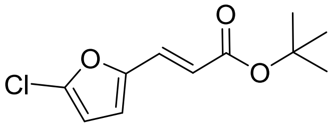

The ylide (*tert*-butoxycarbonyl-methylene)triphenylphosphorane (0.499 g, 1.3 mmol) was dissolved in THF (10 mL). 2-Chlorofuraldehyde (0.145 g, 1.1 mmol) in THF (5 mL) was added over 5 min. After 2 h, the solvent was removed *in vacuo*. Column chromatography on silica, eluting with petroleum ether/EtOAc (20:1) provided the pure product as a white crystalline solid (0.174 g, 71%). Mp 44–47 °C; R_f 0.25 (UV 254 nm, petroleum ether/EtOAc, 20:1); ν_{max}/cm^{-1} 3154, 2975, 1704, 1645, 1156; δH ($CDCl_3$, 400 MHz) 7.20 (1H, d, *J* 15.7, CC**H**), 6.50 (1H, d, *J* 3.4, Ar**H**), 6.21 (1H, d, *J* 15.7, C(O)C**H**), 6.20 (1H, d, *J* 3.4, Ar**H**), 1.48 (9H, s, C(C**H$_3$**)$_3$; δC ($CDCl_3$, 101 MHz) 166.0, 150.7, 138.9, 128.9, 118.3, 115.8, 109.0, 80.5, 28.1; *m*/*z* (NH_3 CI+), Found: MNH_4^+, 246.0905. $C_{11}H_{17}NO_3Cl$ requires 246.0897, Δ 3.3 ppm.

(*E*)-*tert*-Butyl 4-iodocinnamate (3i)

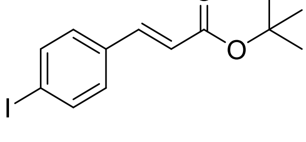

The ylide (*tert*-butoxycarbonyl-methylene)triphenylphosphorane (49 mg, 0.13 mmol) was dissolved in THF (2 mL). 4-Iodobenzaldehyde (25 mg, 0.11 mmol) in THF (1 mL) was added over 5 min. After 16 h, the solvent was removed *in vacuo* to provide the crude product as a yellow oil. Column chromatography on silica, eluting with EtOAc/petroleum ether (1:10) provided the pure product as a

pale yellow solid (28 mg, 79%). Mp 65–67 °C [lit. 65–66°C]; R_f 0.80 (UV 254 nm, EtOAc/petroleum ether, 1:10); δH ($CDCl_3$, 400 MHz) 7.71 (2H, d, *J* 9.0, ArC**H**), 7.51 (1H, d, *J* 16.2, ArC**H**), 7.24 (2H, d, *J* 9.0, Ar**H**), 6.38 (1H, d, *J* 16.2, C(O)C**H**), 1.52 (9H, s, C(C**H₃**)₃); *m/z* (NH_3 CI+), Found: MNH_3^+, 348.0466. $C_{13}H_{19}NO_2I$ requires 348.0461, Δ 1.4 ppm. All data are in agreement with literature values [5].

(*E*)-*tert*-Butyl 2-chlorocinnamate (3j)

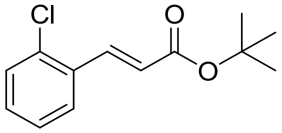

$PdCl_2(PPh_3)_2$ (0.118 g, 0.17 mmol) was added to a Wheaton vial. The vial was flushed with Ar (g), before the addition of Et_3N (12 mL), 1-chloro-2-iodobenzene (0.200 g, 0.84 mmol) and *tert*-butyl acrylate (0.212 mL, 1.68 mmol) sequentially. The vial was sealed and heated to 115 °C. After 18 h, the heat source was removed, and NH_4Cl (sat. aq., 15 mL) was added. The aqueous phase was extracted with CH_2Cl_2 (3 × 15 mL) and the organic phase was dried over Na_2SO_4. The solvent was removed *in vacuo* to provide the crude product as a pale yellow solid (0.447 g). Column chromatography on silica, eluting with EtOAc/Petroleum ether (1:30) provided the pure product as yellow oil (0.186 g, 93%). R_f 0.25 (UV 254 nm, petroleum ether/EtOAc, 30:1); ν_{max}/cm^{-1} 2979, 1714, 1320, 1155; δH ($CDCl_3$, 400 MHz) 8.02 (1H, d, *J* 16.2, ArC**H**), 7.61–7.59 (1H, m, Ar**H**), 7.41–7.38 (1H, Ar**H**), 7.30–7.23 (2H, Ar**H**), 6.38 (1H, d, *J* 15.9), 1.54 (9H, s, C(C**H₃**)₃); δC ($CDCl_3$, 101 MHz) 165.8, 139.4, 134.8, 132.9, 130.7, 130.1, 127.6, 127.0, 122.8, 80.8, 28.2; *m/z* (NH_3 CI+), Found: MNH_4^+, 256.1100. $C_{13}H_{19}NO_2Cl$ requires 256.1104, Δ 1.6 ppm. All data are in agreement with literature values [6].

Synthesis of aziridines 1a–j

General procedure C

$DppONH_2$ [7] (3 equiv) was added to anhydrous CH_2Cl_2 (0.12 M) in a flame-dried flask under Ar (g). NMM (3 equiv) was added dropwise over 1 min, and the reaction mixture was stirred for 30 min. NaOH (11 equiv) (ground to a fine powder immediately prior to use) was added, and the reaction mixture was stirred for a further 20 min. The required cinnamate ester (1 equiv) was then added. After 40 h stirring at rt, NH_4Cl (sat. aq. 60 mL/g) was added, and the aqueous phase extracted with CH_2Cl_2 (3 × 60 mL/g). The organic phase was dried over Na_2SO_4, and the solvent removed *in vacuo*.

General procedure D

$DppONH_2$ [7] (1.04 equiv) was added to anhydrous CH_2Cl_2 (0.24 M) in a flame-dried flask under Ar (g). NMM (1.04 equiv) was added dropwise over 1 min, and the reaction mixture was stirred for

30 min. NaOH (4 equiv) (ground to a fine powder immediately prior to use) was added, and the reaction mixture was stirred for a further 20 min. The required cinnamate ester (1 equiv) was then added. After 40 h stirring at rt, NH_4Cl (sat. aq. 60 mL/g) was added and the aqueous phase extracted with CH_2Cl_2 (3 × 60 mL/g). The organic phase was dried over Na_2SO_4 and the solvent removed *in vacuo*.

3-Phenylaziridine-2-carboxylic acid *tert*-butylester (1a)

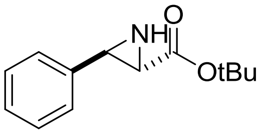

NMM (0.94 mL, 8.6 mmol) was added dropwise over 1 min to a suspension of $DppONH_2$ (2.00 g, 8.6 mmol) in CH_2Cl_2 (24 mL), and the reaction mixture was stirred for 30 min. NaOH (1.26 g, 31.4 mmol) (ground to a fine powder immediately prior to use) was added, and the reaction mixture was stirred for a further 20 min. *tert*-Butyl cinnamate (0.58 g, 2.9 mmol) was then added. After 40 h of stirring at rt, NH_4Cl (sat., aq., 40 mL) was added and the aqueous phase extracted with CH_2Cl_2 (3 × 30 mL). The organic phase was dried over Na_2SO_4 and the solvent removed *in vacuo*. Purification by column chromatography on silica: petroleum ether/EtOAc (10:1) provided the title compound as a white crystalline solid (0.40 g, 65%). Mp 63–66 °C; R_f 0.20 (petroleum ether/EtOAc, 10:1); δ_H ($CDCl_3$, 400 MHz) 7.34–7.27 (5H, Ph**H**), 3.17 (1H, br s, C**H**NH), 2.50 (1H, br s, C**H**NH), 1.82 (1H, br s, NH), 1.50 (9H, s, C(C**H**$_3$)$_3$); δ_C ($CDCl_3$, 101 MHz) 170.8, 138.2, 128.4, 127.7, 126.2, 82.4, 40.4, 39.9, 28.1. All data are in agreement with literature values [7]. A crystal structure was obtained for aziridine **1a**; see crystallographic data.

3-(4-Methoxyphenyl)aziridine-2-carboxylic acid *tert*-butylester (1b)

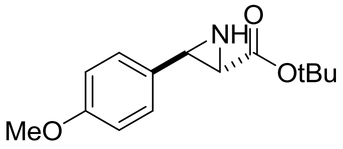

Following general procedure C, 4-methoxy-*tert*-butyl cinnamate (50 mg) provided the title compound as a pale yellow powder (21 mg, 39%) after purification by column chromatography on silica: petroleum ether/EtOAc (10:1). Mp 79–81 °C; R_f 0.40 ($KMnO_4$ dip, petroleum ether/EtOAc, 10:1); ν_{max}/cm^{-1} 3291, 2986, 2947, 1715; δH ($CDCl_3$, 400 MHz) 7.21 (2H, dt, *J* 8.7 and 2.6, Ar**H**), 6.87 (2H, dt, *J* 8.7 and 2.6, Ar**H**), 3.82 (3H, s, OC**H**$_3$), 3.14 (1H, dd, *J* 7.6 and 2.4,C**H**NH), 2.47 (1H, dd, *J* 7.6 and 2.4, C**H**NH), 1.78 (1H, br t, *J* 7.6, CHN**H**),1.53 (9H, s, C(C**H**$_3$)$_3$); δC ($CDCl_3$, 101 MHz) 170.9, 159.3, 130.2, 127.3, 113.9, 82.2, 55.3, 40.3, 39.6, 28.1; *m/z* (electrospray), Found: MNa^+, 272.1269. $C_{14}H_{19}NO_3Na$ requires 272.1263, Δ 2.2 ppm.

3-(4-Methylphenyl)aziridine-2-carboxylic acid *tert*-butylester (1c)

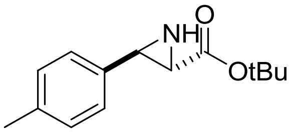

Following general procedure C, 4-methyl-*tert*-butyl cinnamate (50 mg) provided the title compound as a white powder (19 mg, 36%) after purification by column chromatography on silica: petroleum ether/EtOAc (50:1 to 10:1). Mp 62-65 °C; R_f 0.25 ($KMnO_4$ dip, petroleum ether/EtOAc, 10:1); ν_{max}/cm^{-1} 3275, 2981, 2933, 1714; δH ($CDCl_3$, 400 MHz) 7.21–7.14 (4H, m, Ar**H**), 3.14 (1H, dd, 8.8 and 2.7, C**H**NH), 2.48 (1H, dd, *J* 8.8 and 2.7, C**H**NH), 2.36 (3H, s, C**H**$_3$), 1.80 (1H, br t, *J* 8.8, CHN**H**), 1.52 (9H, s, C(C**H**$_3$)$_3$); δ_C ($CDCl_3$, 101 MHz) 170.9, 137.4, 135.2, 129.1, 126.0, 82.3, 40.4, 39.8, 28.1, 21.1; *m*/*z* (NH_3, CI), Found: MH^+, 234.1505. $C_{14}H_{20}NO_2$ requires 234.1494, Δ 4.7 ppm.

3-(2-Chlorophenyl)aziridine-2-carboxylic acid *tert*-butylester (1g)

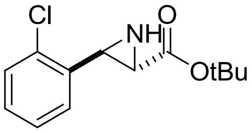

Following general procedure C, 2-chloro-*tert*-butyl cinnamate (50 mg) provided the title compound as a clear oil (25 mg, 47%) after purification by column chromatography on silica: petroleum ether/EtOAc (30:1 to 20:1). R_f 0.20 (UV 254 nm, EtOAc/petroleum ether, 1:20); ν_{max}/cm^{-1} 3293, 2982, 1729, 1229, 1161; δH ($CDCl_3$, 400 MHz) 7.35–7.33 (2H, m, Ar**H**), 7.24–7.17 (2H, m, Ar**H**), 3.45 (1H, br s, ArC**H**), 2.39 (1H, br s, C(O)C**H**), 1.74 (1H, br s, N**H**), 1.51 (9H, s, C(C**H**$_3$)$_3$); δC ($CDCl_3$, 101 MHz) 170.7, 135.8, 134.2, 129.0, 128.6, 127.2, 126.9, 82.4, 39.4, 37.8, 28.1; *m*/*z* (NH_3 CI+), Found: MH^+, 254.0947. $C_{13}H_{17}NO_2Cl$ requires 254.0948, Δ 0.4 ppm.

3-(3-Methoxyphenyl)aziridine-2-carboxylic acid *tert*-butylester (1i)

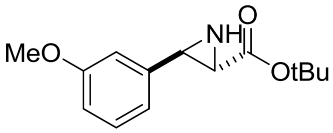

Following general procedure C, 3-methoxy-*tert*-butyl cinnamate (50 mg) provided the title compound as a clear oil (17 mg, 32%) after purification by column chromatography on silica: petroleum ether/EtOAc (20:1 to 10:1). R_f 0.20 (UV 254 nm, EtOAc/petroleum ether, 1:10); ν_{max}/cm^{-1} 2987, 1719, 1234, 1156; δH ($CDCl_3$, 400 MHz) 7.25–7.20 (1H, m, ArC**H**), 6.89 (1H, d, *J* 7.3, Ar**H**), 6.83–6.80 (2H, Ar**H**), 3.80 (3H, s, OC**H**$_3$), 3.15 (1H, d, *J* 2.3 ArC**H**), 2.50 (1H, d, *J* 2.3,

C(O)C**H**), 1.74 (1H, br s, N**H**), 1.50 (9H, s, C(C**H**$_3$)$_3$); δC ($CDCl_3$, 101 MHz) 170.7, 159.8, 139.9, 129.4, 118.7, 113.6, 111.1, 82.3, 55.3, 40.4, 39.9, 28.1; *m*/*z* (NH_3 CI+), Found: MH^+, 250.1441. $C_{14}H_{20}NO_3$ requires 250.1441, Δ 0 ppm.

3-(4-Iodophenyl)aziridine-2-carboxylic acid *tert*-butylester (1d)

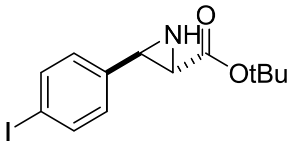

Following general procedure D, 4-iodo-*tert*-butyl cinnamate (50 mg) provided the title compound as a white solid (17 mg, 33%) after purification by column chromatography on silica: petroleum ether/EtOAc (30:1 to 20:1). Mp 83–85 °C; R_f 0.40 (UV 254 nm, EtOAc/petroleum ether, 1:20); ν_{max}/cm^{-1} 3273, 2979, 1717, 1360, 1151; δH ($CDCl_3$, 400 MHz) 7.64 (2H, d, *J* 8.5, Ar**H**), 7.03 (2H, d, *J* 8.5, Ar**H**), 3.11 (1H, d, *J* 2.0, ArC**H**), 2.44 (1H, d, *J* 2.0, C(O)C**H**), 1.76 (1H, br s, N**H**), 1.49 (9H, s, C(C**H**$_3$)$_3$); δC ($CDCl_3$, 101 MHz) 170.5, 138.0, 137.4, 128.1, 93.0, 82.6, 40.5, 39.4, 28.1; *m*/*z* (NH_3 CI+), Found: MH^+, 346.0295. $C_{13}H_{17}NO_2I$ requires 346.0304, Δ 2.6 ppm.

3-(4-Chloro)phenylaziridine-2-carboxylic acid *tert*-butylester (1e)

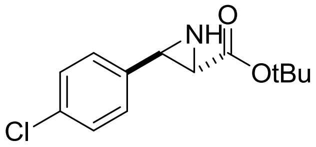

Following general procedure D, 4-chloro-*tert*-butyl cinnamate (57 mg) provided the title compound as a white oil (32 mg, 53%) after purification by column chromatography on silica: petroleum ether/EtOAc (15:1). R_f 0.35 ($KMnO_4$ dip, petroleum ether/EtOAc, 15:1); ν_{max}/cm^{-1} 3289, 2984, 2933, 1723; δ_H ($CDCl_3$, 400 MHz) 7.33–7.29 (2H, m, Ar**H**), 7.26–7.23 (2H, m, Ar**H**), 3.16 (1H, br d, *J* 4.8, C**H**NH), 2.48 (1H, br s, C**H**NH), 1.86 (1H, br s, CHN**H**), 1.53 (9H, s, C(C**H**$_3$)$_3$); δ_C ($CDCl_3$, 101 MHz) 170.5, 136.8, 133.4, 128.5, 127.5, 82.5, 40.5, 39.2, 28.1; *m*/*z* (NH_3 chemical), Found: MH^+, 254.0952. $C_{13}H_{17}NO_2Cl$ requires 254.0948, Δ 1.6 ppm.

3-(4-Nitrophenyl)aziridine-2-carboxylic acid *tert*-butylester (1f)

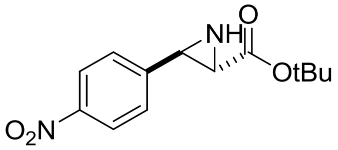

Following general procedure D, 4-nitro-*tert*-butyl cinnamate (63 mg) provided the title compound as a yellow powder (51 mg, 76%) after purification by column chromatography on silica: petroleum

ether/EtOAc (5:1). Mp 90-92 °C; R_f 0.25 ($KMnO_4$ dip, petroleum ether/EtOAc, 5:1); ν_{max}/cm^{-1} 3281, 2984, 2972, 1709; δ_H ($CDCl_3$, 400 MHz) 8.22–8.19 (2H, m, Ar**H**), 7.50–7.46 (2H, m, Ar**H**), 3.27 (1H, br s, C**H**NH), 2.52 (1H, br s, C**H**NH), 1.97 (1H, br s, CHN**H**), 1.54 (9H, s, C(C**H**3)3); δ_C ($CDCl_3$, 101 MHz) 169.9, 147.5, 145.8, 127.1, 123.7, 83.0, 41.0, 39.0, 28.0; *m*/*z* (NH_3, CI+), Found: MH^+, 265.1196. $C_{13}H_{17}N_2O_4$ requires 265.1188, Δ 3.0 ppm.

3-(2-Nitrophenyl)aziridine-2-carboxylic acid *tert*-butylester (1h)

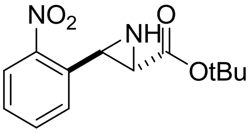

Following general procedure D, 2-nitro-*tert*-butyl cinnamate (50 mg) provided the title compound as a white oil (21 mg, 40%) after purification by column chromatography on silica: petroleum ether/EtOAc (10:1). R_f 0.40 (UV 254 nm, EtOAc/petroleum ether, 1:10); ν_{max}/cm^{-1} 3298, 2991, 1722, 1525, 1346; δH ($CDCl_3$, 400 MHz) 8.09 (1H, dd, *J* 8.4 and 1.0, Ar**H**), 7.74 (1H, d, *J* 7.5, Ar**H**), 7.62 (1H, t, *J* 7.5, Ar**H**), 7.45 (1H, t, *J* 8.4, Ar**H**), 3.69 (1H, d, *J* 5.8, ArC**H**), 2.41 (1H, d, *J* 5.8, C(O)C**H**), 1.86 (1H, m, N**H**), 1.53 (9H, s, C(C**H₃**)₃); δC ($CDCl_3$, 101 MHz) 170.4, 148.7, 134.0, 133.8, 128.9, 128.3, 124.7, 82.7, 39.5, 38.1, 28.0; *m*/*z* (NH_3 CI+), Found: MH^+, 265.1190. $C_{13}H_{17}N_2O_4$ requires 265.1188, Δ 0.8 ppm.

3-(5-Chlorofuran-2-yl)aziridine-2-carboxylic acid *tert*-butylester (1j)

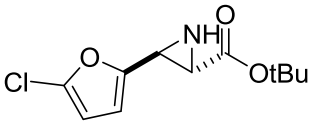

Following general procedure D, quenching the reaction after 20 h, 5-chloro-*tert*-butyl furfural (50 mg) provided the title compound as a yellow oil (19 mg, 35%) after purification by column chromatography on silica: petroleum ether/EtOAc (10:1). R_f 0.25 (UV 254 nm, petroleum ether/EtOAc, 10:1); ν_{max}/cm^{-1} 2987, 1722, 1531, 1233, 1158; δH ($CDCl_3$, 400 MHz) 6.27 (1H, d, *J* 3.1, Ar**H**), 6.09 (1H, d, *J* 3.1, Ar**H**), 3.15 (1H, dd, *J* 9.1 and 2.3, C**H**N), 2.76 (1H, dd, *J* 9.1 and 2.3, C**H**N), 1.75 (1H, br t, *J* 9.1, N**H**), 1.50 (9H, s, C(C**H₃**)₃); δC ($CDCl_3$, 101 MHz) 170.1, 150.9, 135.7, 109.6, 107.1, 82.8, 37.6, 33.7, 28.0; *m*/*z* (ESI+), Found: MH_3O^+, 262.0832. $C_{11}H_{17}NO_4Cl$ requires 262.0846, Δ 5.3 ppm.

Ring-opening products 2a–i

tert-Butyl 2-amino-3-phenyl-3-(indol-3-yl)propanoate (2a)

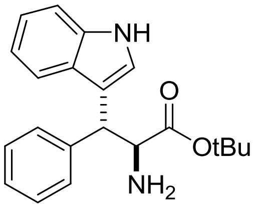

Aziridine **1a** (25 mg, 0.11 mmol) and indole (27 mg, 0.23 mmol) were dissolved in CH_2Cl_2 (1 mL), and cooled to 0 °C. BF_3·THF (19 µl, 0.17 mmol) was added over 30 s. The mixture was stirred at 0 °C for 5 min, before warming to rt, and after 24 h, the reaction was quenched with $NaHCO_3$ (5 mL). The aqueous phase was extracted with CH_2Cl_2 (3 × 5 mL), dried over Na_2SO_4, and concentrated *in vacuo* to provide the crude product as yellow oil (59 mg). Column chromatography eluting with petroleum ether/EtOAc (3:2) provided the pure product as a white solid (24 mg, 63%). Mp 167–169 °C, [lit: 175–176 °C [8]] R_f 0.10 (UV 254 nm, petroleum ether/EtOAc, 3:2); ν_{max}/cm^{-1} 3419, 2962, 2928, 2547, 1722; δH ($CDCl_3$, 500 MHz) 8.22 (1H, br s, ArNH), 7.49 (1H, d, *J* 8.0, Ar**H**), 7.40 (2H, d, *J* 7.5, Ar**H**), 7.33–7.25 (4H, Ar**H**), 7.23 (1H, tt, *J* 7.5 and 1.5, Ar**H**), 7.17 (1H, m, Ar**H**), 7.06 (1H, m, Ar**H**), 4.65 (1H, d, *J* 6.5, ArC**H**), 4.20 (1H, br s, NH_2C**H**), 1.72 (2H, br s, NH_2), 1.26 (9H, s, C(C**H**)$_3$); δ_C ($CDCl_3$, 101 MHz) 173.7, 140.6, 136.1, 129.0, 128.4, 127.0, 126.8, 122.3, 122.0, 119.4, 119.4, 116.6, 111.0, 81.2, 59.5, 47.3, 27.8; *m*/*z* (NH_3, CI+), Found: MH^+, 337.1916. $C_{21}H_{25}N_2O_2$ requires 337.1916, Δ 0 ppm.

Phenylalanine-_tert_-butyl ester (2b)

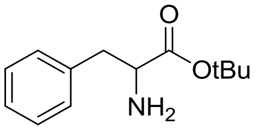

Aziridine **1a** (25 mg, 0.11 mmol) and Pd/C (5 mg, 20% w/w) were added to MeOH (1 mL). The flask was evacuated and refilled with H_2 (g), and the reaction mixture was stirred under a H_2 atmosphere. After 3 h, the Pd/C was removed by filtration, and the filtrate washed with CH_2Cl_2 (10 mL). The solvent was removed *in vacuo* to provide the pure product as a clear oil (24 mg, 95%). R_f 0.45 (UV 254 nm, EtOAc/MeOH, 20%); ν_{max}/cm^{-1} 3388, 2984, 1730; δH ($CDCl_3$, 400 MHz) 7.31–7.20 (5H, Ar**H**), 3.63 (1H, br t, *J* 6.2, NH_2C**H**), 3.06 (1H, dd, *J* 13.8 and 6.2, ArC**H**H), 2.87 (1H, dd, *J* 13.8 and 8.0, ArCH**H**), 1.52 (2H, br s, NH_2), 1.42 (9H, s, C(C**H$_3$**)$_3$; δC ($CDCl_3$, 101 MHz) 174.3, 137.6, 129.4, 128.4, 126.7, 81.2, 56.3, 41.3, 28.0; *m*/*z* (ESI+) $C_{13}H_{20}NO_2$ found 222 (M+), 150 (- OtBu). All data are in agreement with literature values [9].

***tert*-Butyl 2-amino-3-phenyl-3-(methoxy)propanoate (2c)**

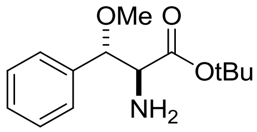

CSA (27 mg, 0.11 mmol) was added to anhydrous MeOH (1 mL) at 0 °C. After 2 min, the ice bath was removed and aziridine **1a** (25 mg, 0.11 mmol) added immediately. After 6 h, the reaction was quenched with $NaHCO_3$ (sat. aq., 5 mL). The aqueous phase was extracted with CH_2Cl_2 (3 × 5 mL), and the combined organic phases were dried over Na_2SO_4. The solvent was removed *in vacuo* to provide the pure product as a clear oil (29 mg, quantitative). R_f 0.45 (UV 254 nm, EtOAc/MeOH, 2%); ν_{max}/cm^{-1} 3399, 2982, 1735, 1370, 1159; δH ($CDCl_3$, 400 MHz) 7.41–7.28 (5H, Ar**H**), 4.39 (1H, d, *J* 5.9, ArC**H**), 3.67 (1H, d, *J* 5.9, C(O)C**H**), 3.27 (3H, s, OC$\mathbf{H_3}$), 1.58 (2H, br s NH_2), 1.42 (9H, s, C(C$\mathbf{H_3}$)$_3$); δC ($CDCl_3$, 101 MHz) 172.1, 137.4, 128.3, 128.3, 127.6, 85.4, 81.4, 60.5, 57.2, 28.0; *m/z* (NH_3, CI+), Found: MH^+, 252.1602. $C_{14}H_{22}NO_3$ requires 252.1600, Δ 0.8 ppm.

***tert*-Butyl 2-amino-3-phenyl-3-(allyloxy)propanoate (2d)**

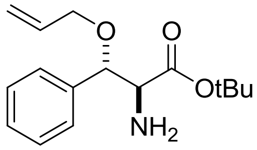

CSA (66 mg, 0.29 mmol) was added to allyl alcohol (1 mL) at 0 °C. After 2 min, the ice bath was removed and aziridine **1a** (25 mg, 0.11 mmol) added immediately. After 16 h, the reaction was quenched with $NaHCO_3$ (sat. aq., 5 mL). The aqueous phase was extracted with CH_2Cl_2 (3 × 5 mL), and the combined organic phases were dried over Na_2SO_4. The solvent was removed *in vacuo* to provide the crude product as a yellow oil (30 mg). Column chromatography, eluting with EtOAc/petroleum ether (1:10) then (1:2) provided the pure product as a clear oil (18 mg, 57%). R_f 0.25 ($KMnO_4$ dip, EtOAc/petroleum ether, 1:2); ν_{max}/cm^{-1} 2984, 2867, 1737, 1368, 1156; δH ($CDCl_3$, 400 MHz) 7.37–7.29 (5H, m, Ar**H**), 5.91–5.81 (1H, m, H_2C=C**H**), 5.25 (1H, dq, J_{trans} 17.5 and 1.5, **H**HC=CH), 5.16 (1H, dquart, J_{cis} 10.4 and 1.5 H**H**C=CH), 4.52 (1H, d, *J* 7.2, ArC**H**), 3.98 (1H, ddt, *J* 12.8, 5.7 and 1.5, OCH**H**), 3.82 (1H, ddt, *J* 12.8, 5.7 and 1.4, OC**H**H), 3.66 (1H, d, *J* 6.5, NC**H**), 1.47 (2H, br s, NH_2), 1.43 (9H, s, C(C$\mathbf{H_3}$)$_3$); δC ($CDCl_3$, 101 MHz) 172.3, 137.8, 134.5, 128.3, 128.3, 127.7, 116.9, 83.1, 81.4, 69.9, 60.7, 28.0; *m/z* (NH_3, CI+), Found: MH^+, 278.1766. $C_{16}H_{24}NO_3$ requires 278.1756, Δ 4.0 ppm.

tert-Butyl 2-amino-3-phenyl-3-(benzyloxy)propanoate (2e)

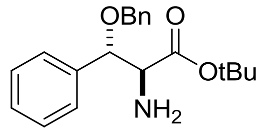

CSA (66 mg, 0.29 mmol) was added to benzyl alcohol (1 mL) at 0 °C. After 2 min of stirring, the ice bath was removed and aziridine **1a** (25 mg, 0.11 mmol) added immediately. After 16 h of stirring under Ar (g), the reaction was quenched with $NaHCO_3$ (sat. aq., 5 mL). The aqueous phase was extracted with hexane (3 × 5 mL). The organic phases were combined and washed with H_2O (3 × 8 mL) and dried over Na_2SO_4. The solvent was removed *in vacuo*. Column chromatography, eluting with EtOAc/petroleum ether (1:5) then (1:3) provided the pure product as a clear oil (17 mg, 46%). R_f 0.20 (UV 254 nm, EtOAc/petroleum ether, 1:3); ν_{max}/cm^{-1} 2982, 1730, 1372, 1153; δH ($CDCl_3$, 400 MHz) 7.39–7.27 (10H, Ar**H**), 4.61 (1H, d, *J* 6.5 BnOC**H**), 4.51 (1H, d, *J* 11.8, PhC**H**H), 4.35 (1H, d, *J* 11.8, PhCH**H**), 3.72 (1H, d, *J* 6.5, NC**H**), 1.67 (2H, br s, NH_2), 1.41 (9H, s, C(C**H**$_3$)$_3$); δC ($CDCl_3$, 101 MHz) 172.0, 137.9, 137.6, 128.4, 128.4, 128.3, 127.8, 127.7, 127.6, 83.2, 81.4, 70.8, 60.7, 28.0; *m*/*z* (NH_3, CI+), Found: MH^+, 328.1903. $C_{20}H_{26}NO_3$ requires 328.1913, Δ 3.0 ppm.

tert-Butyl 2-acetylamino-3-phenyl-3-(hydroxy)propanoate (2f)

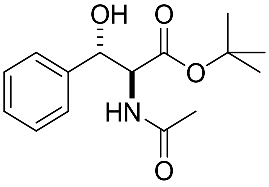

tert-Butyl aziridine **1a** (25 mg, 0.11 mmol) was suspended in acetic acid (2 mL) and heated to 70 °C. After 2 h, the heat source was removed and the reaction mixture cooled. The acetic acid was removed *in vacuo*, and the resulting white crystals dissolved in CH_2Cl_2 (4 mL). The organic phase was washed with $NaHCO_3$ and the aqueous phase extracted with dichloromethane (3 × 4 mL). The organic phase was dried over $MgSO_4$ and concentrated *in vacuo* to provide the pure product (27 mg, 79%). Mp 173–174 °C; R_f 0.20 ($KMnO_4$ dip, EtOAc/Hexane, 1:1); ν_{max}/cm^{-1} 3357 (br), 3266, 2931, 2861, 1734 (s), 1652 (s); δ_H ($CDCl_3$, 400 MHz) 7.34–7.24 (5H, m, Ar**H**), 6.42 (1H, d, *J* 6.7, NH), 5.26 (1H, d, *J* 3.0, ArC**H**), 4.90 (1H, dd, *J* 6.7 and 3.0, COC**H**), 4.30 (1H, br s, O**H**), 2.02 (3H, s, COC**H**$_3$), 1.41 (9H, s, OC(C**H**$_3$)$_3$); δ_C ($CDCl_3$, 101 MHz) 171.7, 168.4, 139.5, 128.1, 127.8, 126.0, 83.4, 75.3, 59.8, 27.9, 23.0; *m*/*z* (NH_3, CI+), Found: MH^+, 280.1551. $C_{15}H_{22}NO_4$ requires 280.1549, Δ 0.7 ppm.

3-Chloro-3-phenylalanine hydrochloride (2g)

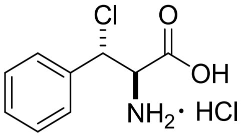

tert-Butyl aziridine **1a** (25 mg, 0.11 mmol) was dissolved in CH_2Cl_2 (1 mL) and HCl (37% aq., 10 drops) added. After 20 min, a white precipitate was seen forming. After 2 h, the precipitate was filtered off and washed with CH_2Cl_2 (4 mL). The precipitate was dried *in vacuo* for 15 min, providing the pure compound as a white flaky solid (12 mg, 45%). Mp 164–166 °C; R_f 0.0 (UV 254 nm, petroleum ether/EtOAc, 3:1); ν_{max}/cm^{-1} 2978 (br), 2904, 1739, 1208, 820; δH (d_6-DMSO, 400 MHz) 8.98 (2.5H, br s, O**H**/NH_2), 7.60–7.55 (2H, Ar**H**), 7.49–7.40 (3H, Ar**H**), 5.86 (1H, d, *J* 4.1, ArC**H**), 4.59 (1H, d, 4.28), 3.86 (0.5H, br s, O**H**/NH_2); δC (CD_3OD, 101 MHz) 166.5, 134.4, 129.3, 128.6, 127.5, 59.3, 59.2; *m*/*z* (ESI+), Found: MH^+, 200.0473. $C_9H_{11}NO_2Cl$ requires 200.0478, Δ 2.5 ppm.

3-Chloro-3-phenylalanine *tert*-butyl ester hydrochloride (2h)

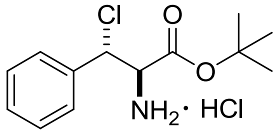

tert-Butyl aziridine **1a** (25 mg, 0.11 mmol) dissolved in CH_2Cl_2 (1 mL) and HCl (2 mL, 4 M in dioxan) was added. After 30 min, the reaction solvent was removed *in vacuo* to yield the crude product as an off-white solid. Et_2O (10 mL) was added, and the insoluble white solid was collected by filtration. Removal of the solvent from the solid *in vacuo* yielded the pure product as a white solid (12 mg, 36 %). R_f 0.0 (UV 254 nm, EtOAc/MeOH 5%); m.p. 155–158 °C; ν_{max}/cm^{-1} 2967, 1729, 1264, 1154; δH (CD_3OD, 400 MHz) 7.59 (2H, Ar**H**), 7.52 (3H, Ar**H**), 5.68 (1H, d, *J* 4.4, ArC**H**), 4.65 (1H, d, *J* 4.4, NH_2C**H**), 1.43 (9H, s, C(C$\mathbf{H_3}$)$_3$); δC (CD_3OD, 101 MHz) 164.4, 134.8, 129.1, 128.5, 127.4, 84.8, 59.7, 59.2, 26.6; *m*/*z* (ESI+), Found: MH^+, 256.1096. $C_{13}H_{19}NO_2Cl$ requires 256.1104, Δ 3.1 ppm.

3-Chloro-3-phenylalanine *tert*-butyl ester (2i)

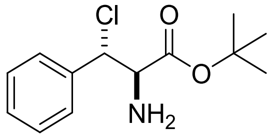

tert-Butyl aziridine **1a** (24 mg, 0.11 mmol) was dissolved in CH_2Cl_2 (1 mL), and HCl (2 mL, 4 M in dioxan) was added. After 1 h, the reaction solvent was removed *in vacuo* to yield the product as the hydrochloride salt. The salt was dissolved in CH_2Cl_2 (4 mL) and washed with $NaHCO_3$ (4 mL, sat. aq). The organic phase was dried over Na_2SO_4 and the solvent removed *in vacuo*, to provide the title

compound as a clear oil (25 mg, 90%). R_f 0.8 (UV 254 nm, EtOAc/MeOH 5%); ν_{max}/cm^{-1} 3394, 2979, 1731, 1371, 1153; δH ($CDCl_3$, 400 MHz) 7.43–7.32 (5H, Ar**H**), 5.06 (1H, d, *J* 7.2, ArC**H**), 3.87 (1H, d, *J* 7.2, C(O)C**H**), 1.52 (2H, br s, NH), 1.47 (9H, s, $C(C\mathbf{H_3})_3$); δC ($CDCl_3$, 101 MHz) 170.9, 137.3, 128.9, 128.6, 128.2, 82.3, 64.0, 62.2, 28.0; *m*/*z* (NH_3, CI+), Found: MH^+, 256.1105. $C_{13}H_{19}NO_2Cl$ requires 256.1104, Δ 0.4 ppm.

NMR Spectra of compounds 3a, 1b–j, 2a–i

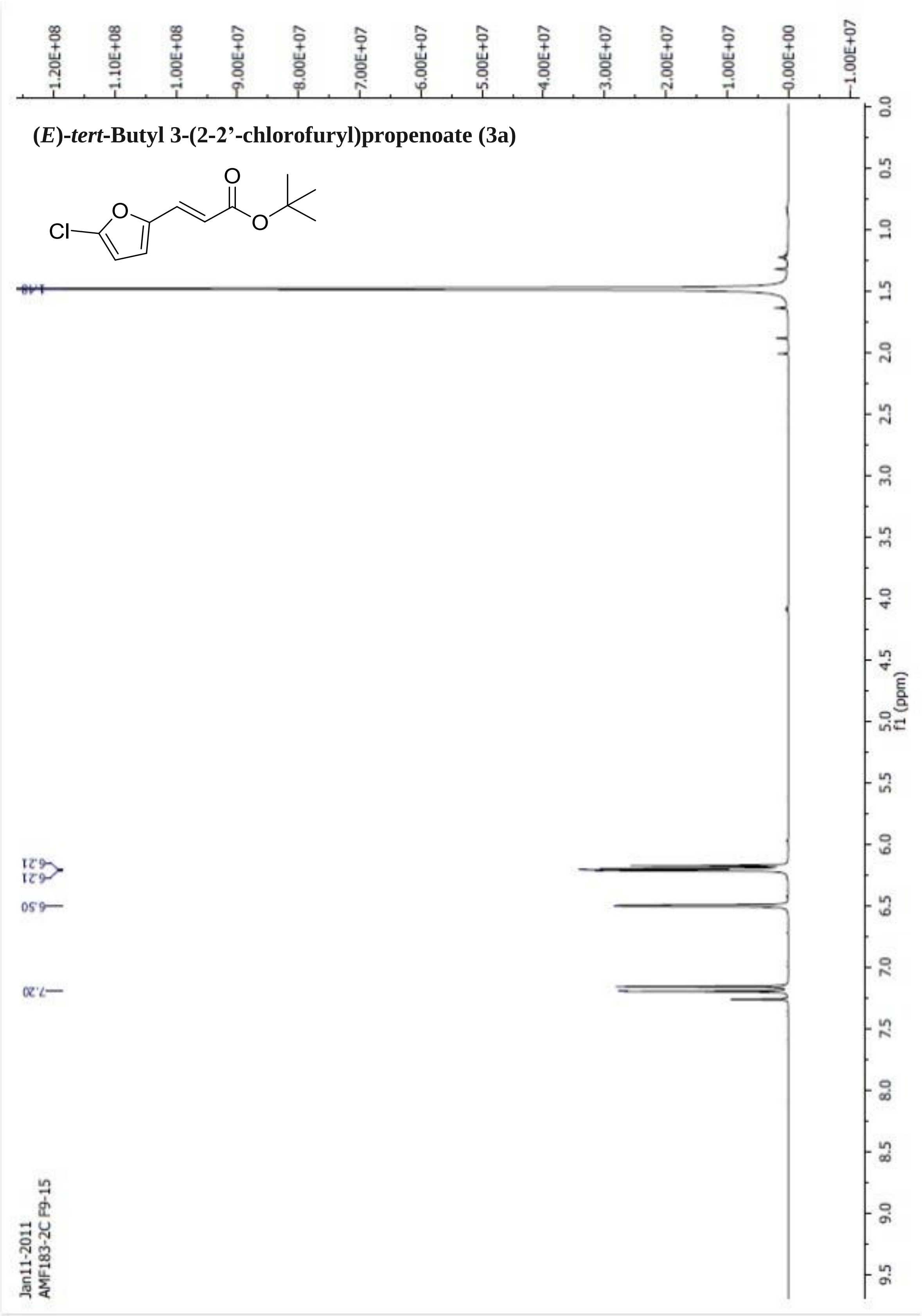

(*E*)-*tert*-Butyl 3-(2-2'-chlorofuryl)propenoate (3a)

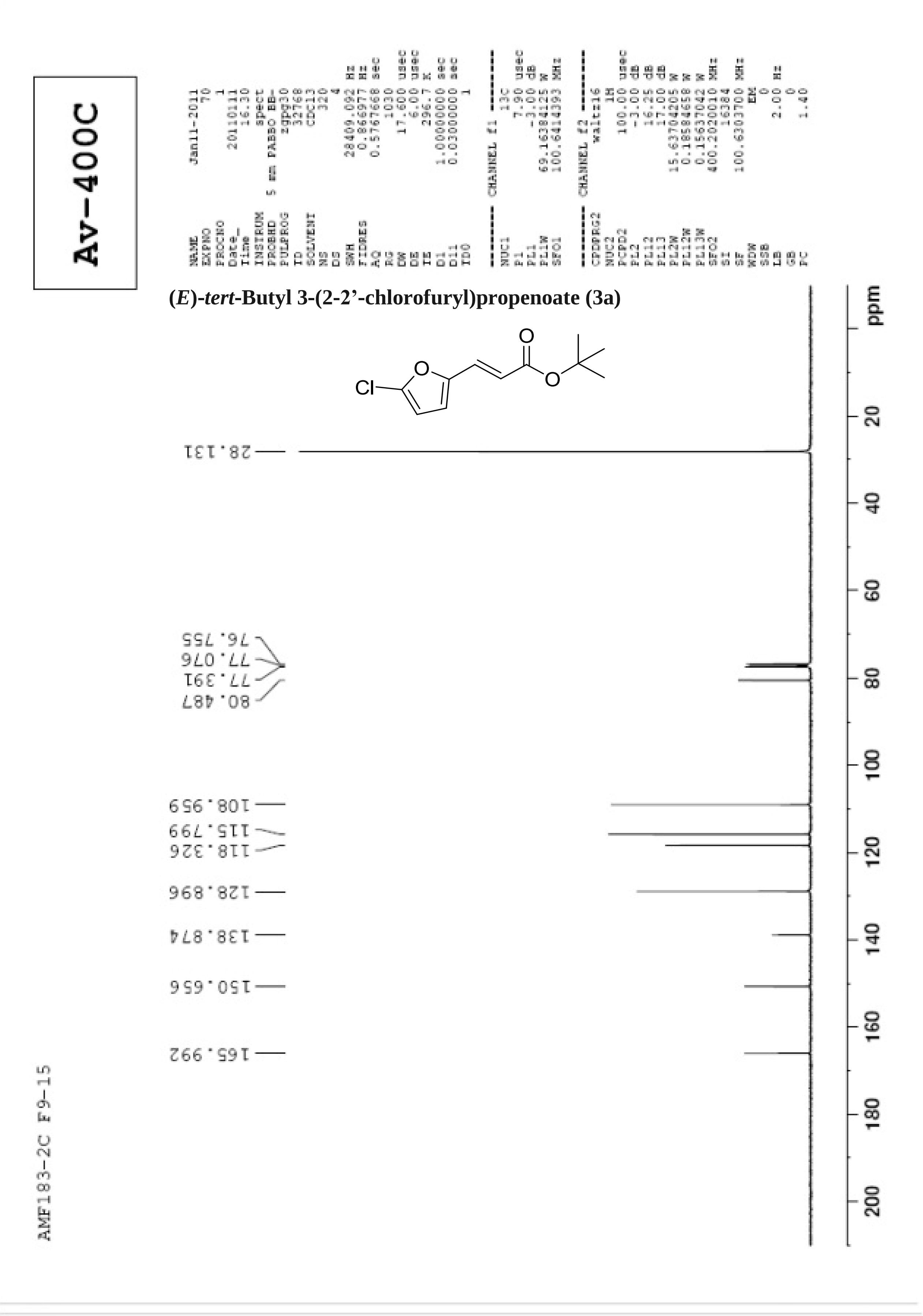

***(E)-tert*-Butyl aziridine-3-(4-methoxyphenyl)prop-2-enoate (1b)**

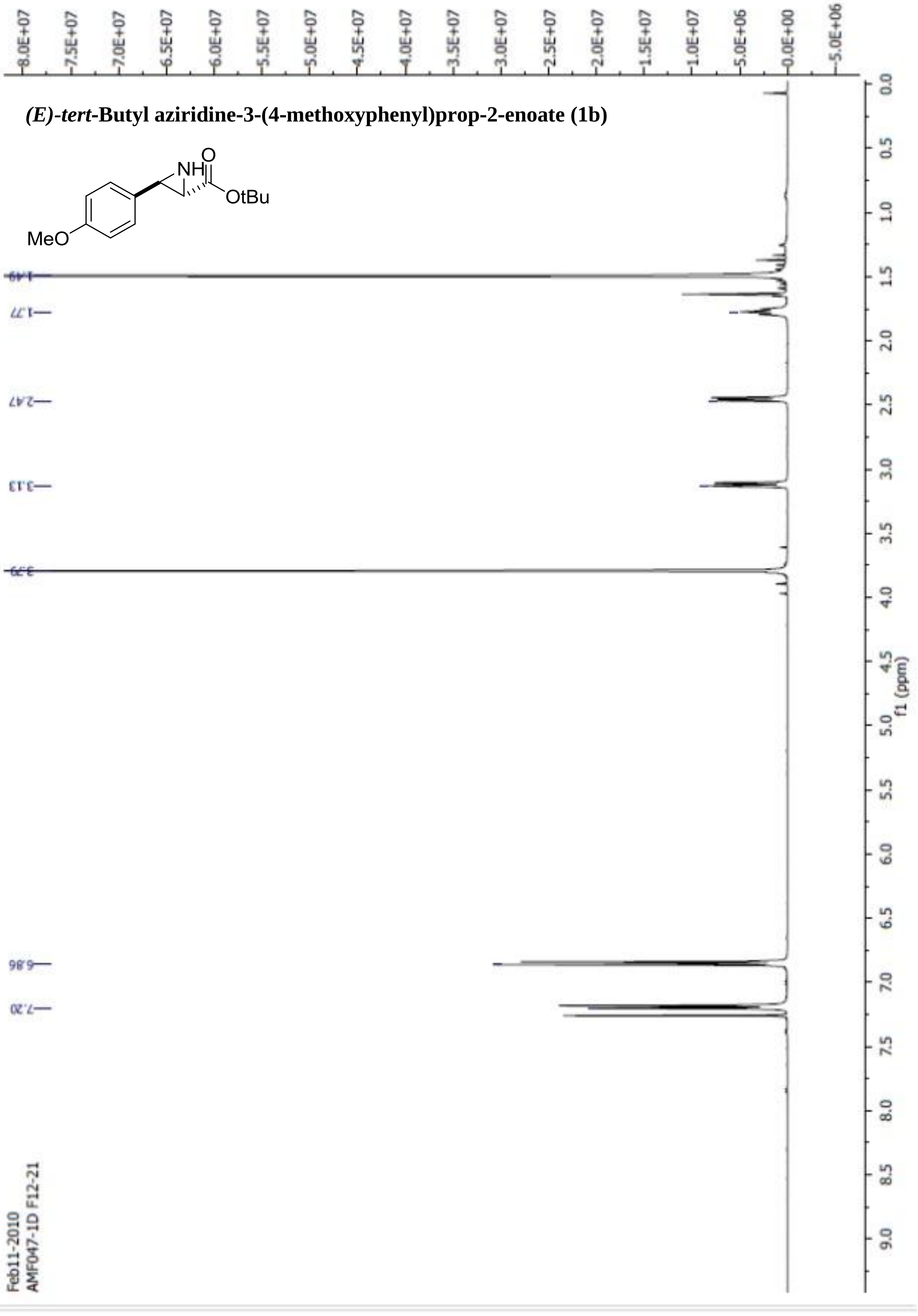

tert-Butyl (_E_)-aziridine-3-(4-methoxyphenyl)prop-2-enoate (1b)

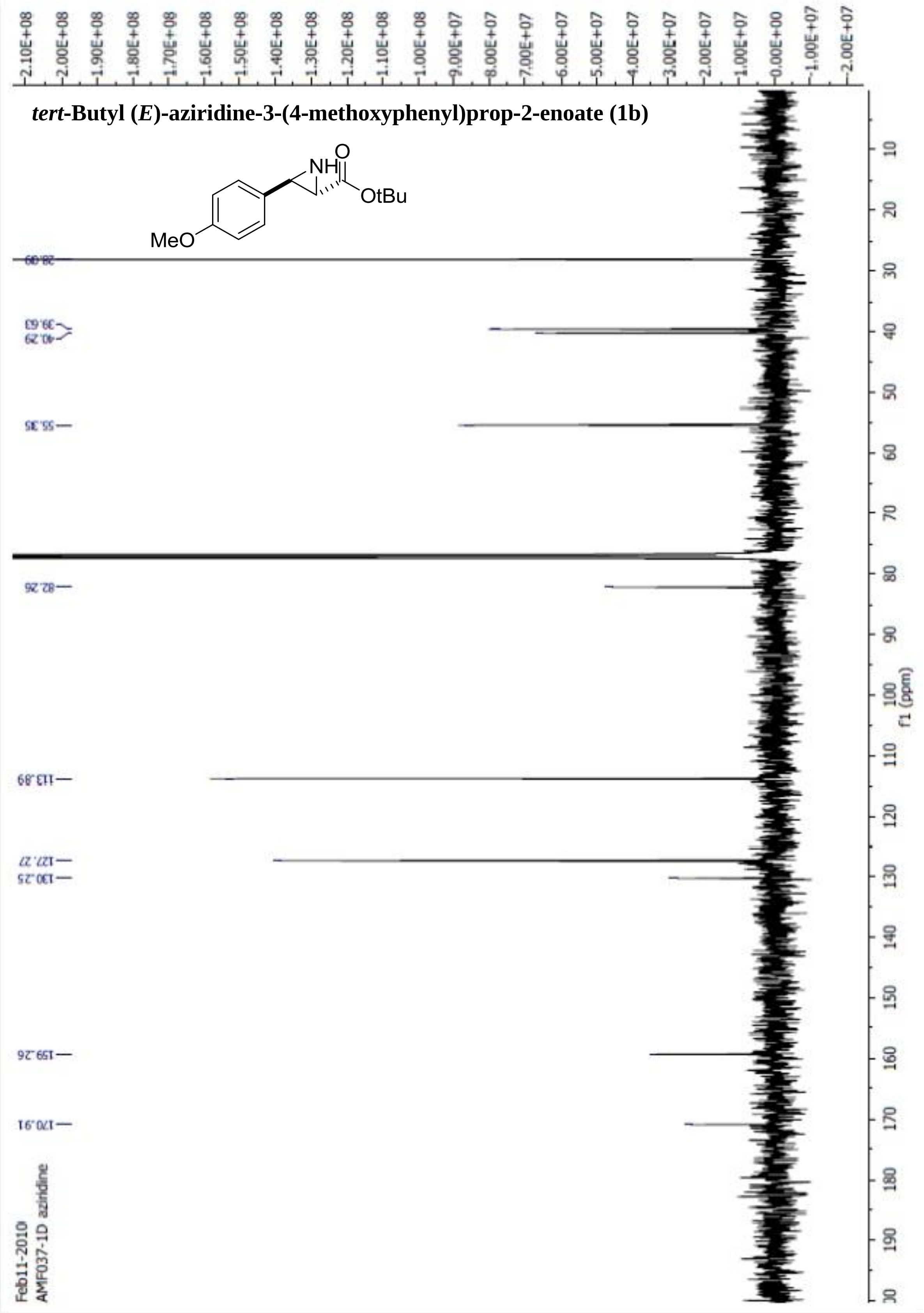

***tert*-Butyl (*E*)aziridine-3-(4-methylphenyl)prop-2-enoate (1c)**

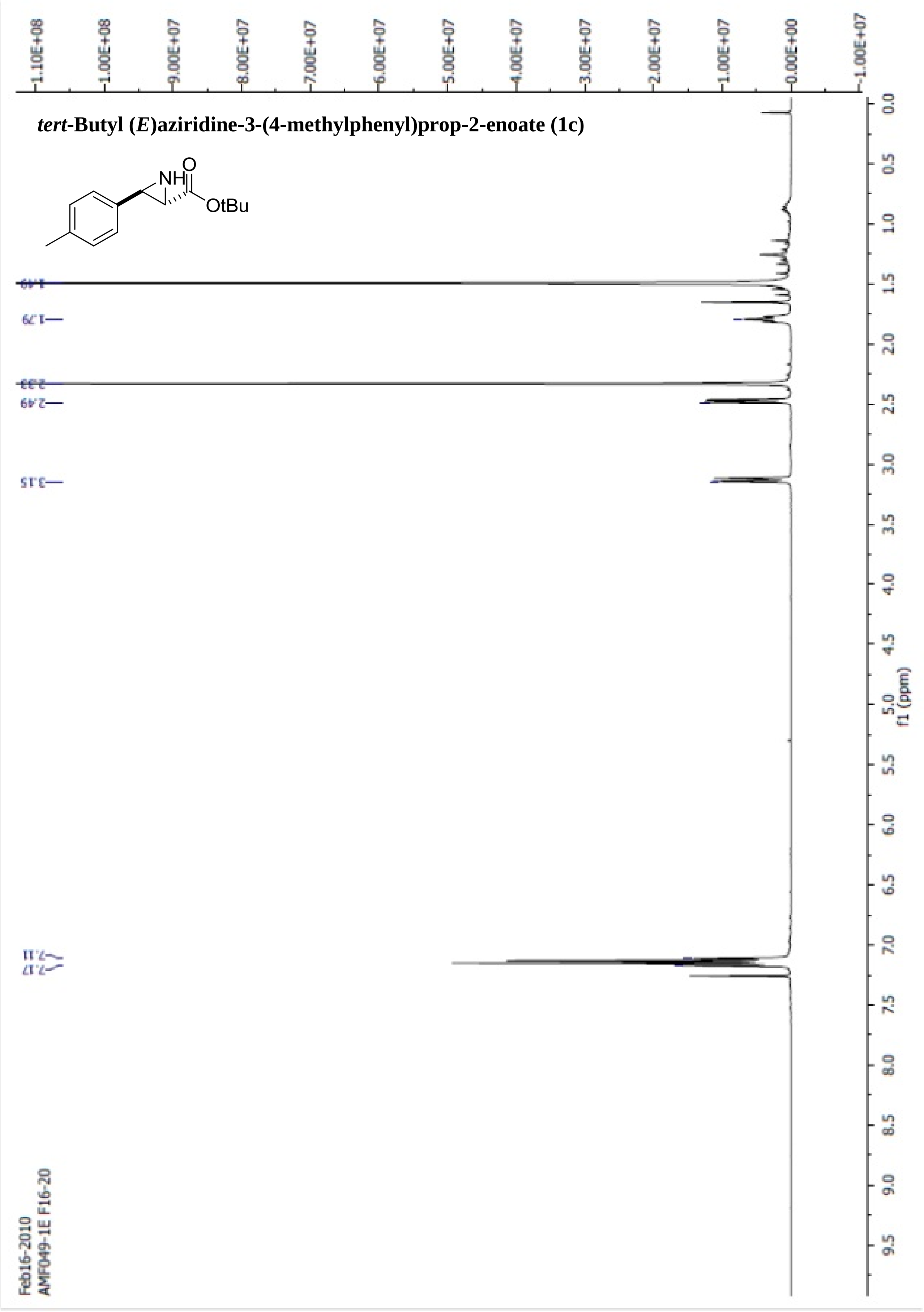

***tert*-Butyl (*E*)-aziridine-3-(4-methylphenyl)prop-2-enoate (1c)**

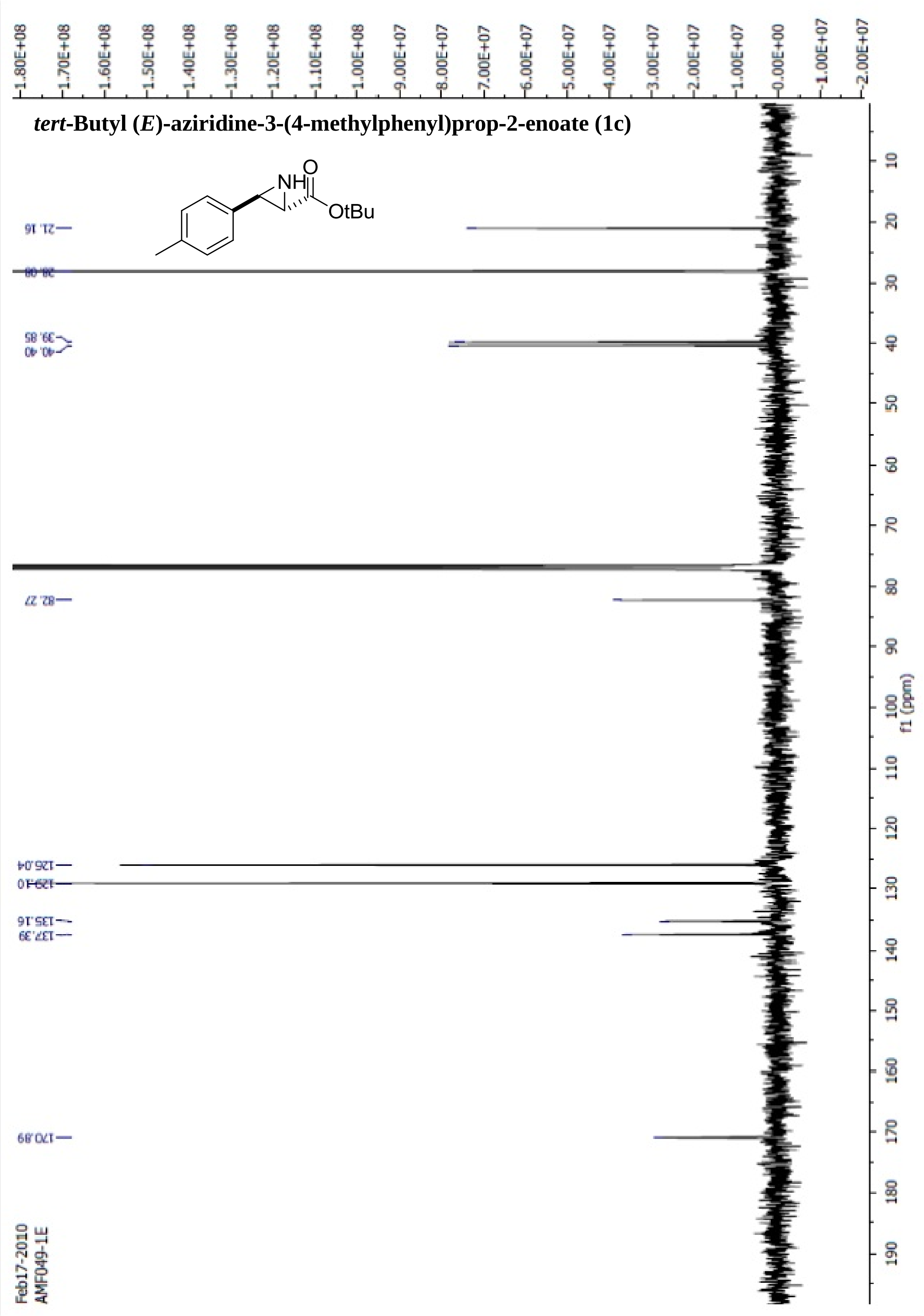

***tert*-Butyl (*E*)-aziridine-3-(4-chlorophenyl)prop-2-enoate (1e)**

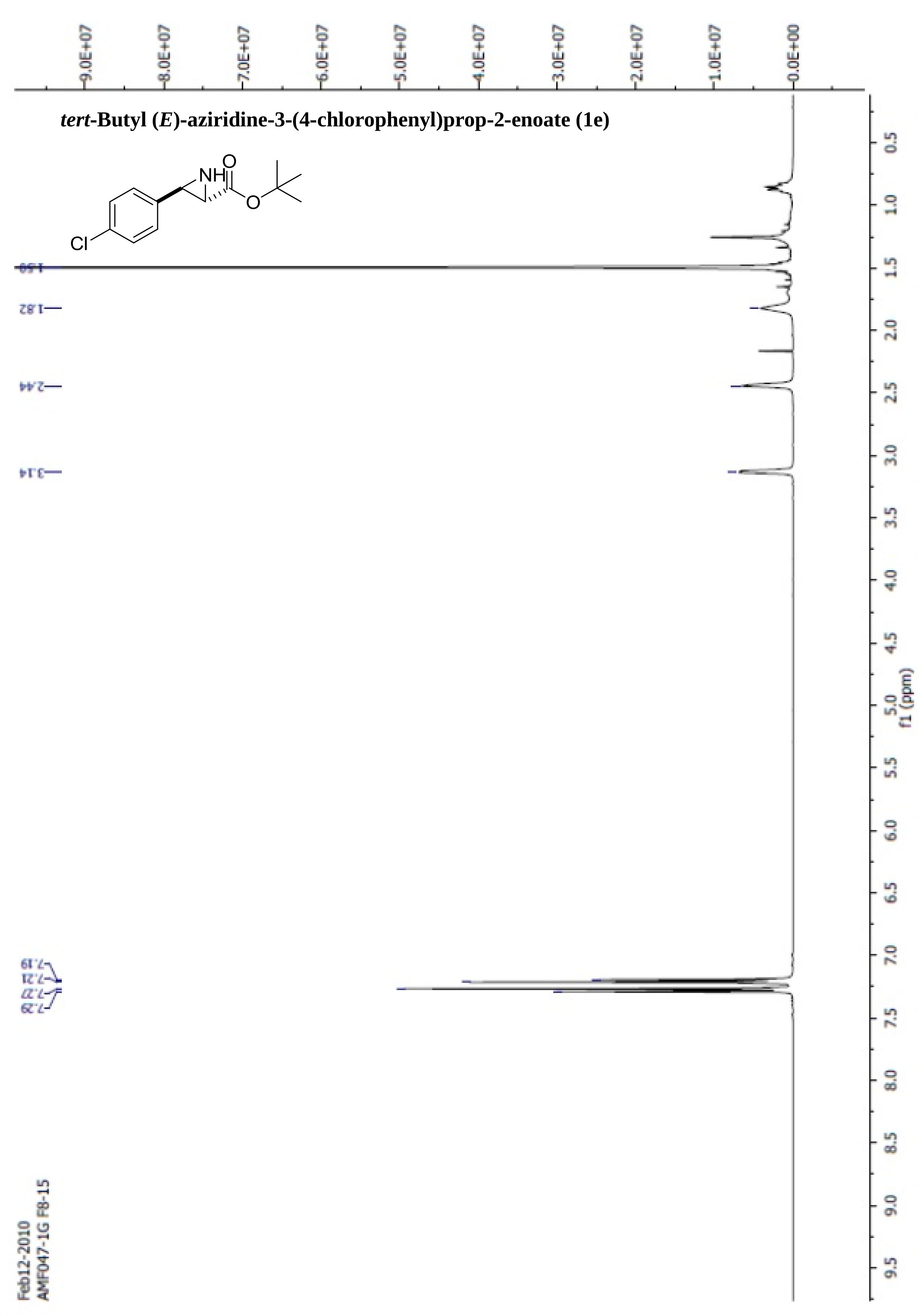

***tert*-Butyl (*E*)-aziridine-3-(4-chlorophenyl)prop-2-enoate (1e)**

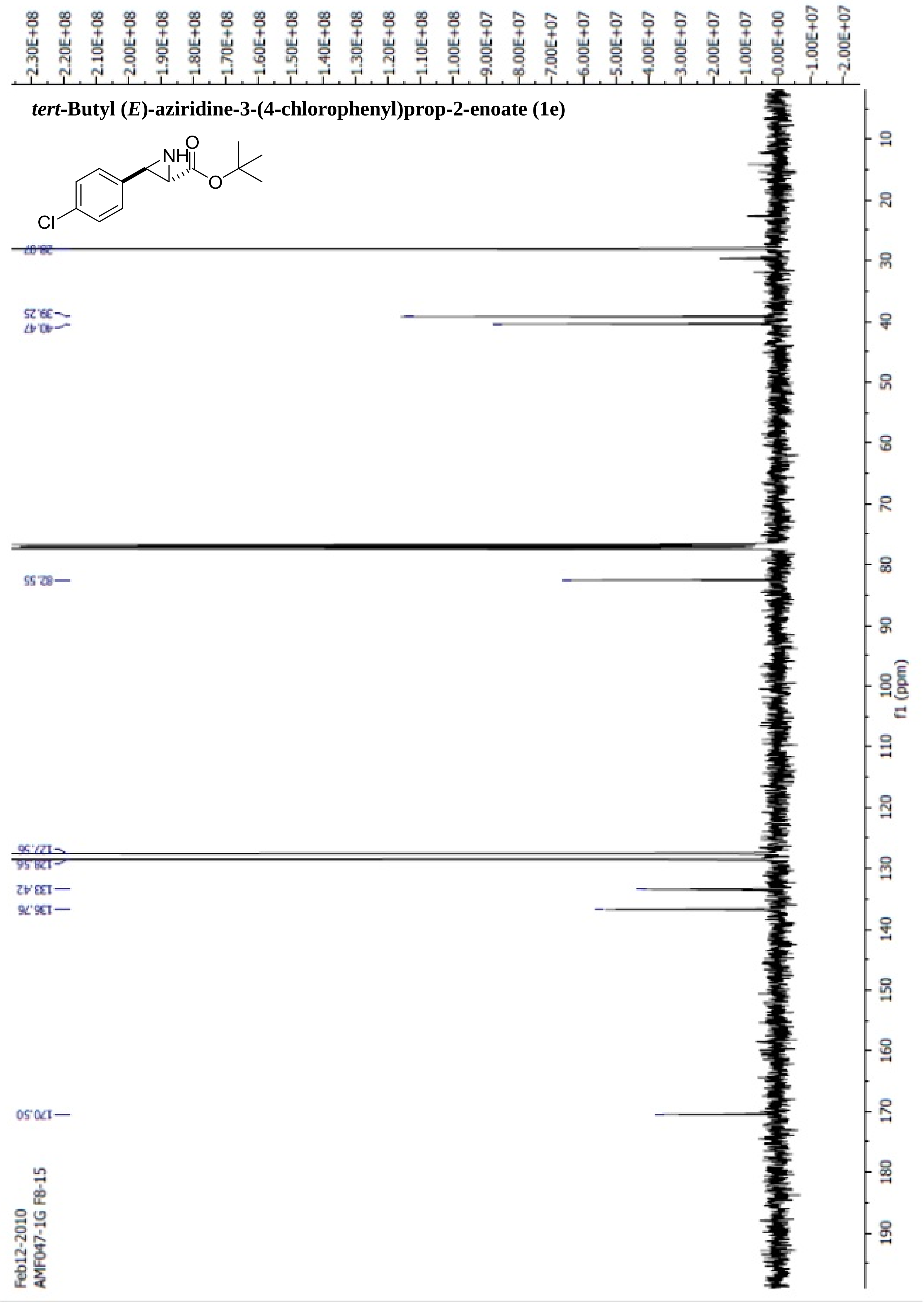

***tert*-Butyl (*E*)-aziridine-3-(4-nitrophenyl)prop-2-enoate (1f)**

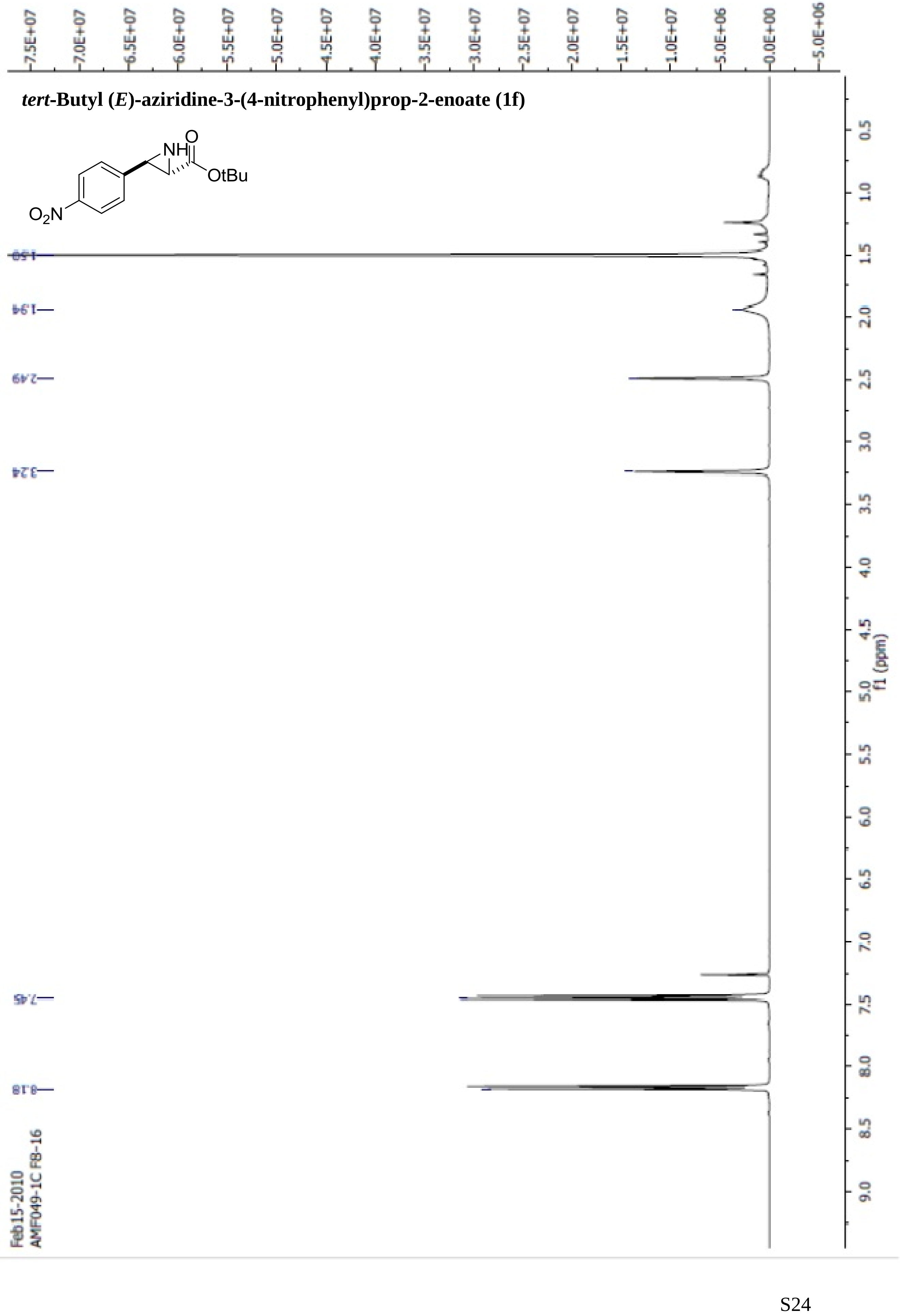

***tert*-Butyl (*E*)-aziridine-3-(4-nitrophenyl)prop-2-enoate (1f)**

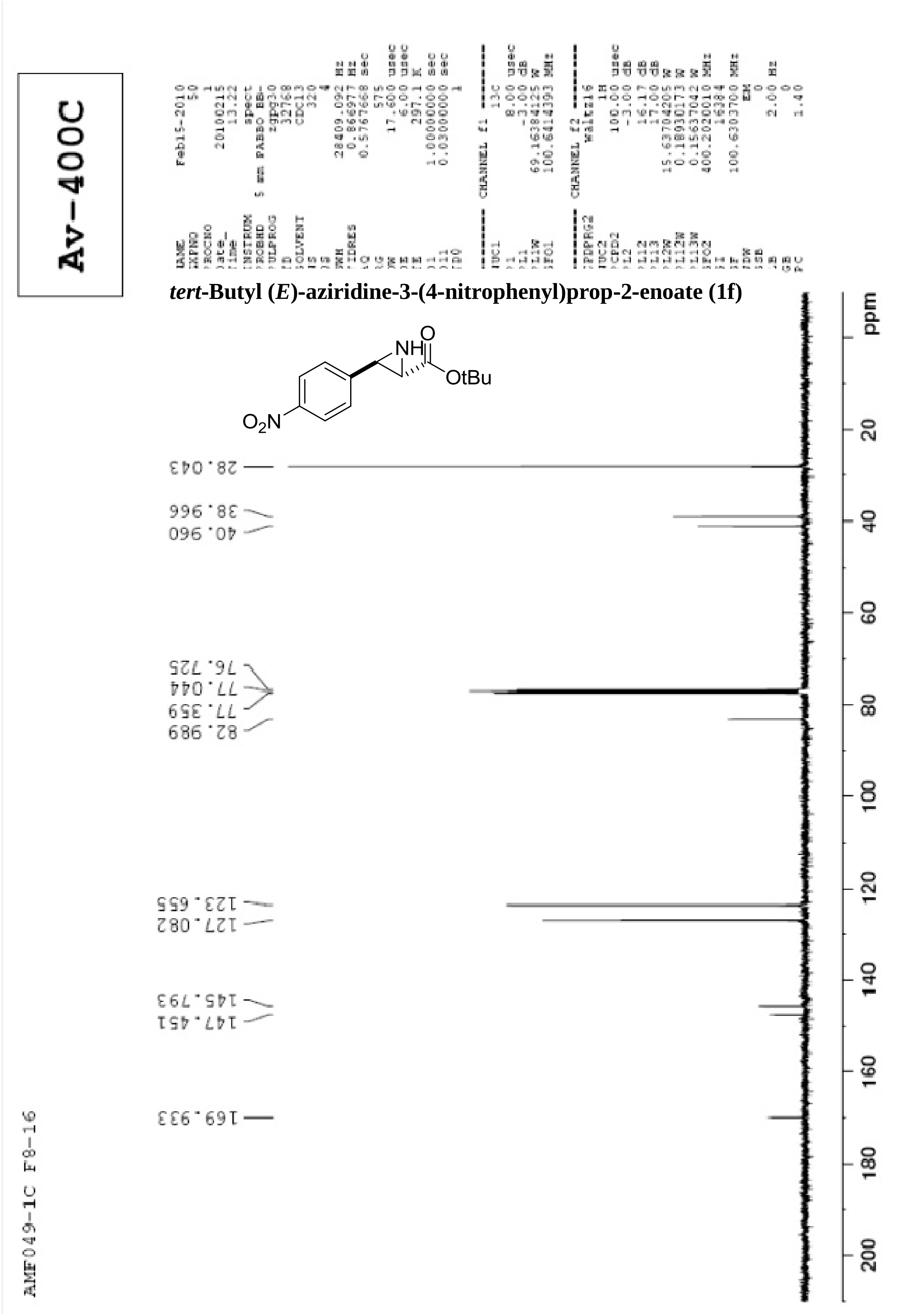

***tert*-Butyl (*E*)-aziridine-3-(2-chlorophenyl)prop-2-enoate (1g)**

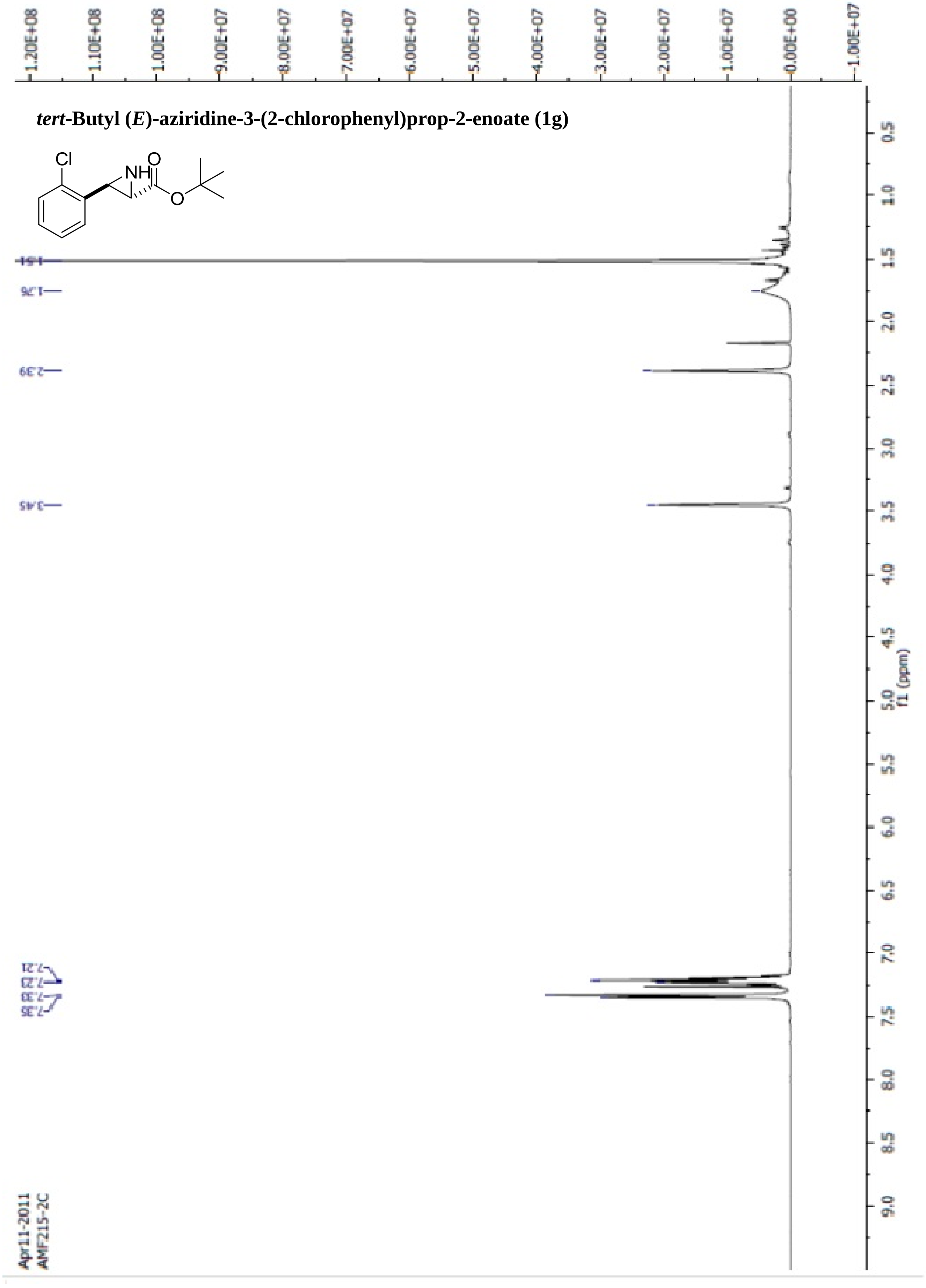

***tert*-Butyl (*E*)-aziridine-3-(2-chlorophenyl)prop-2-enoate (1g)**

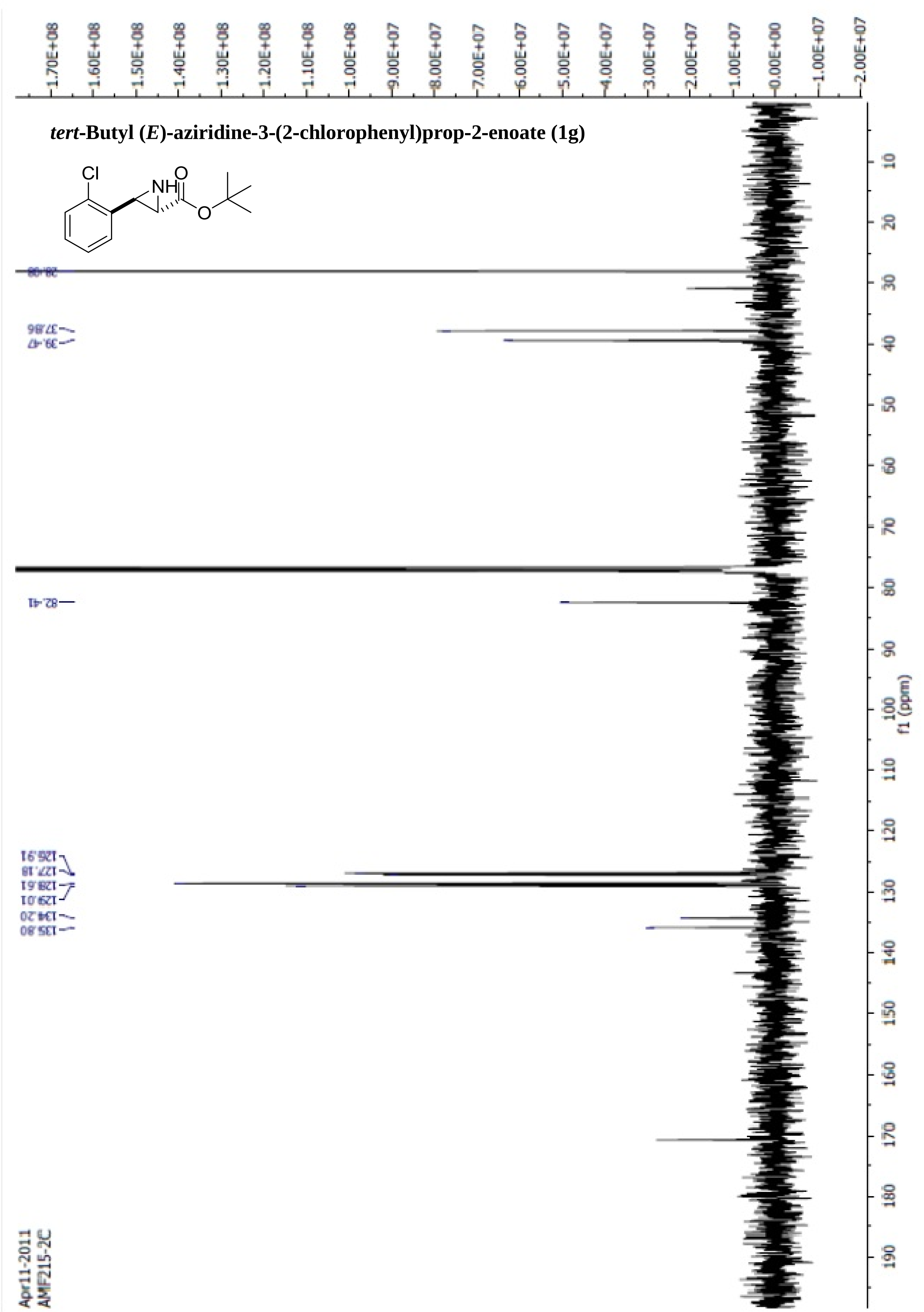

***tert*-Butyl (*E*)-aziridine-3-(3-methoxyphenyl)prop-2-enoate (1i)**

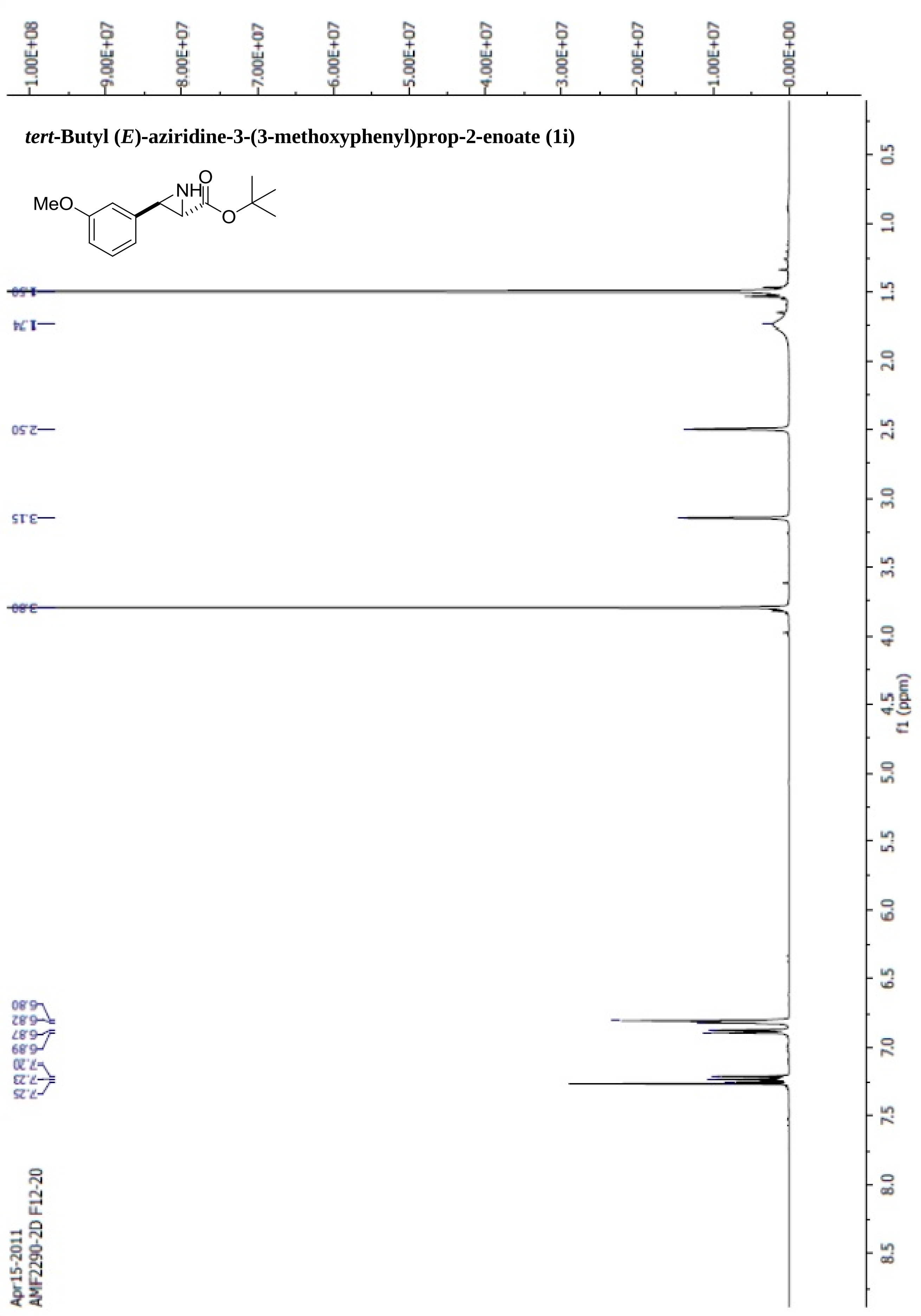

***tert*-Butyl (*E*)-aziridine-3-(3-methoxyphenyl)prop-2-enoate (1i)**

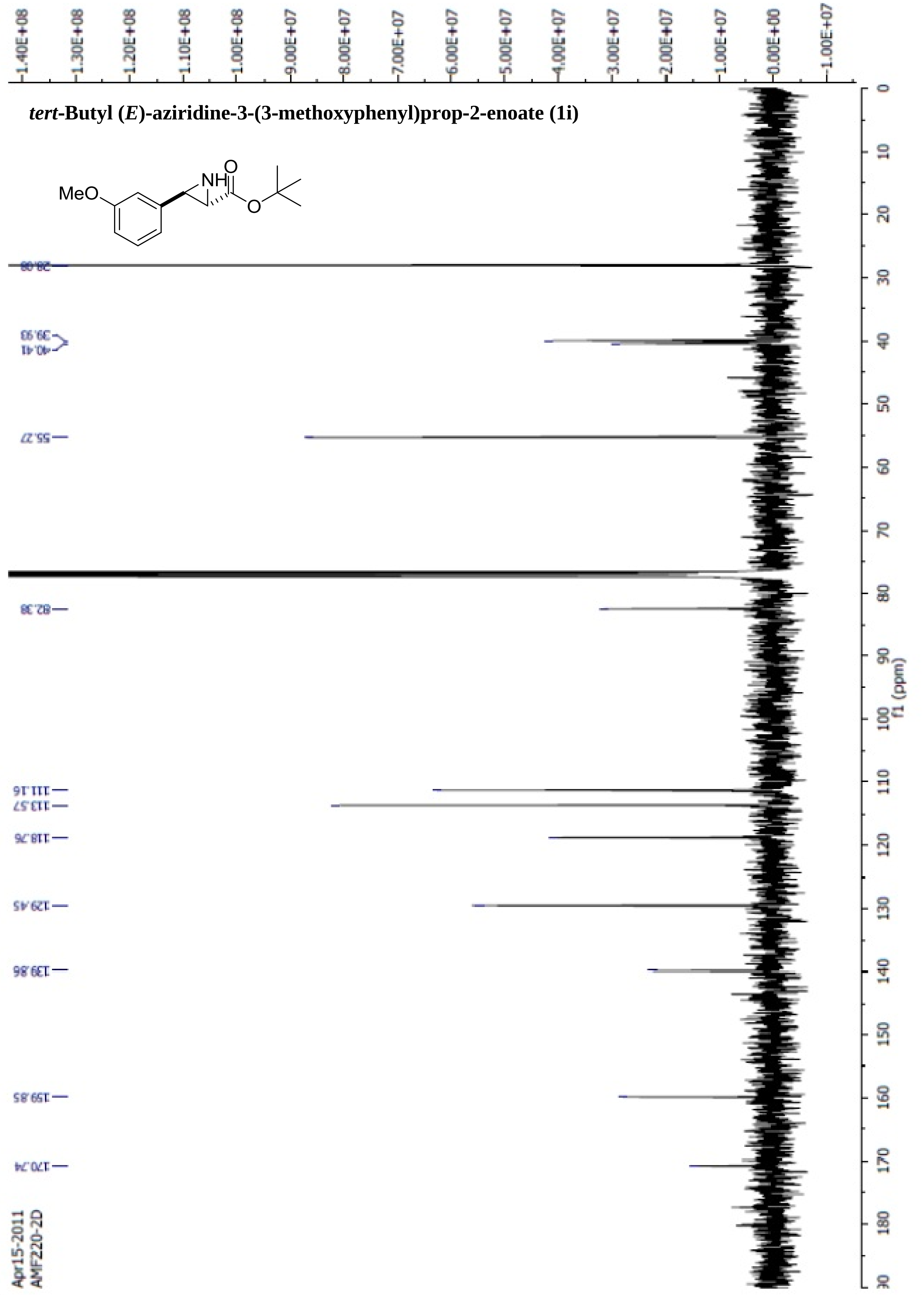

***tert*-Butyl (*E*)-aziridine-3-(4-iodophenyl)prop-2-enoate (1d)**

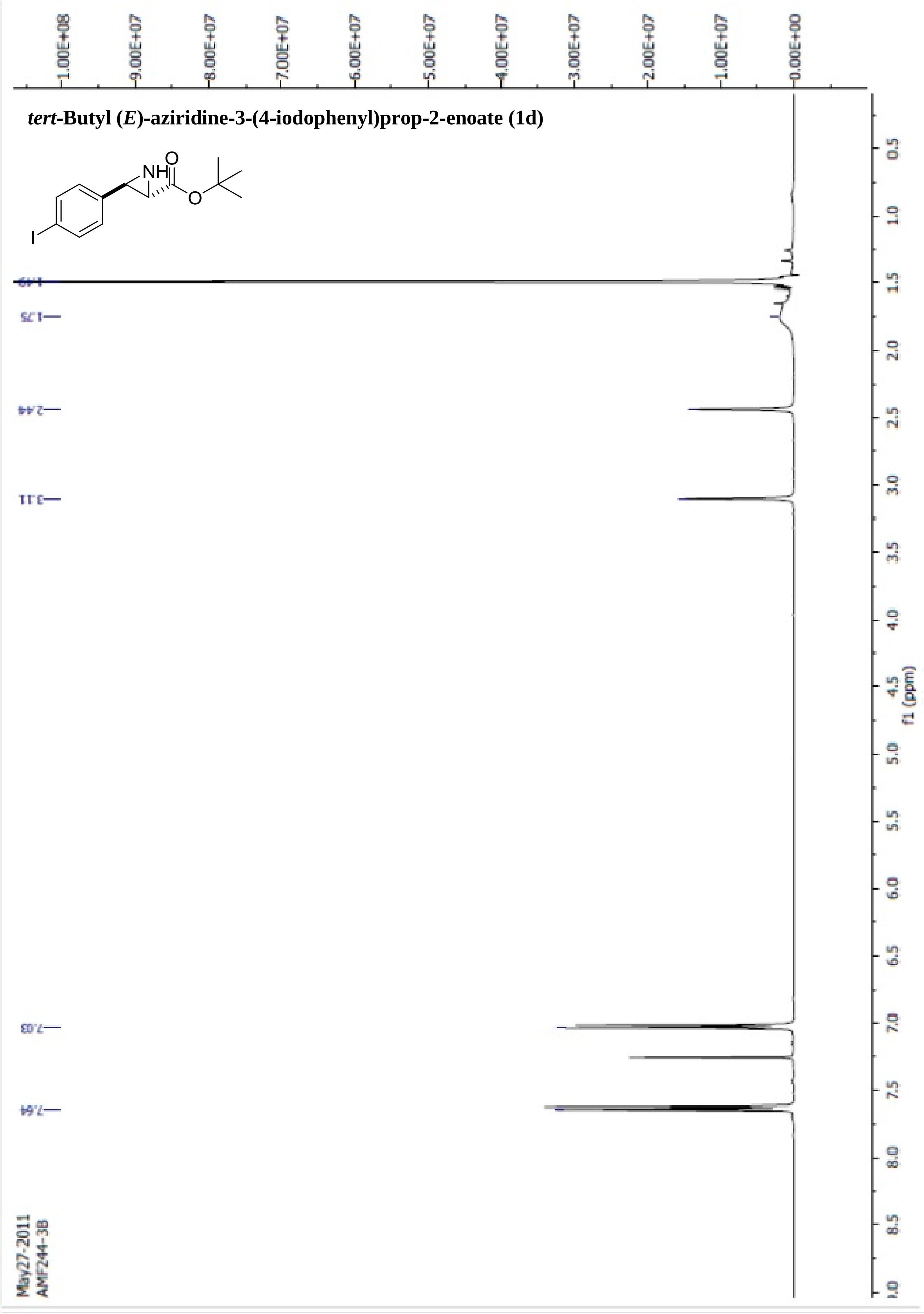

***tert*-Butyl (*E*)-aziridine-3-(4-iodophenyl)prop-2-enoate (1d)**

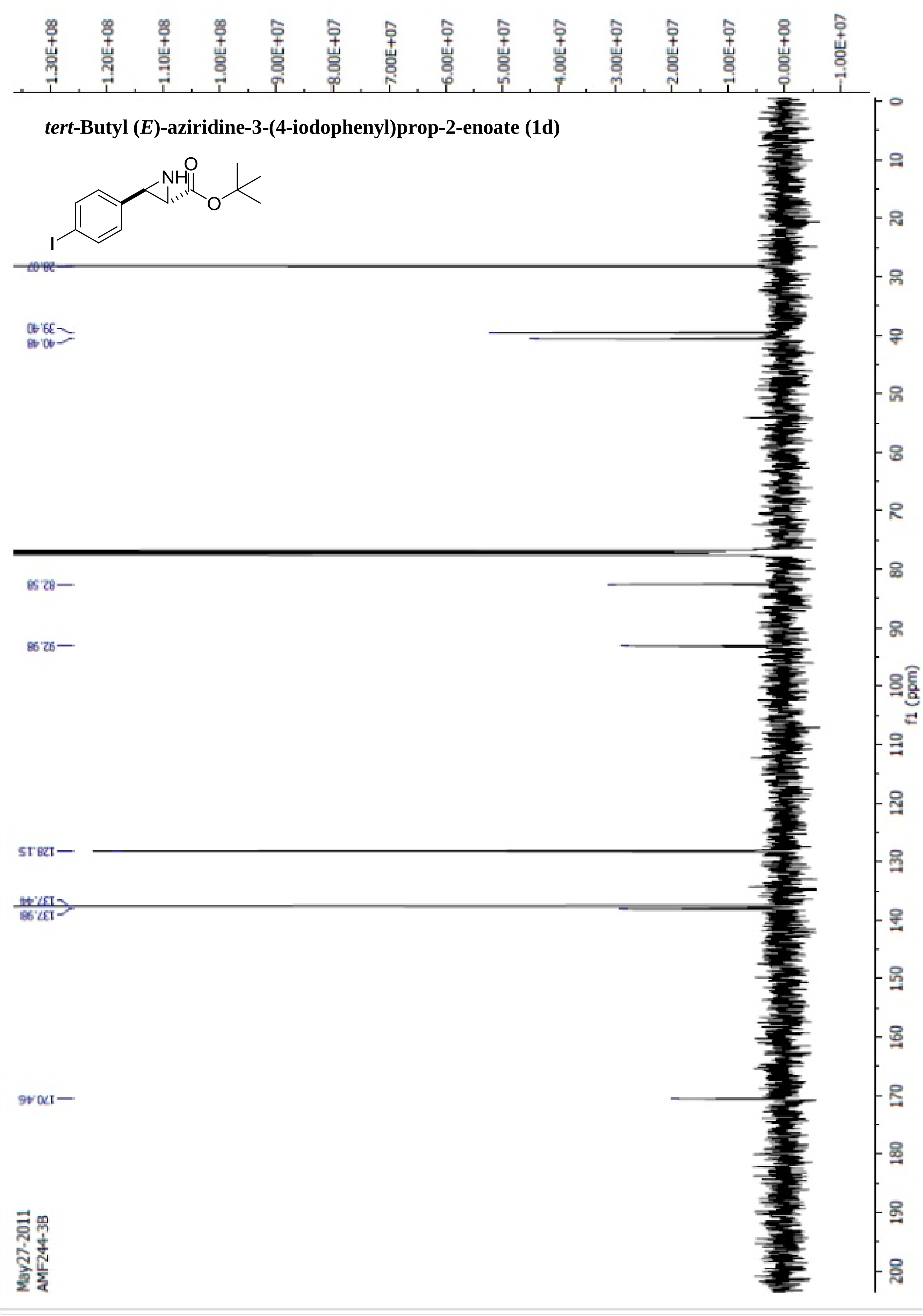

***tert*-Butyl (*E*)-aziridine-3-(2-nitrophenyl)prop-2-enoate (1h)**

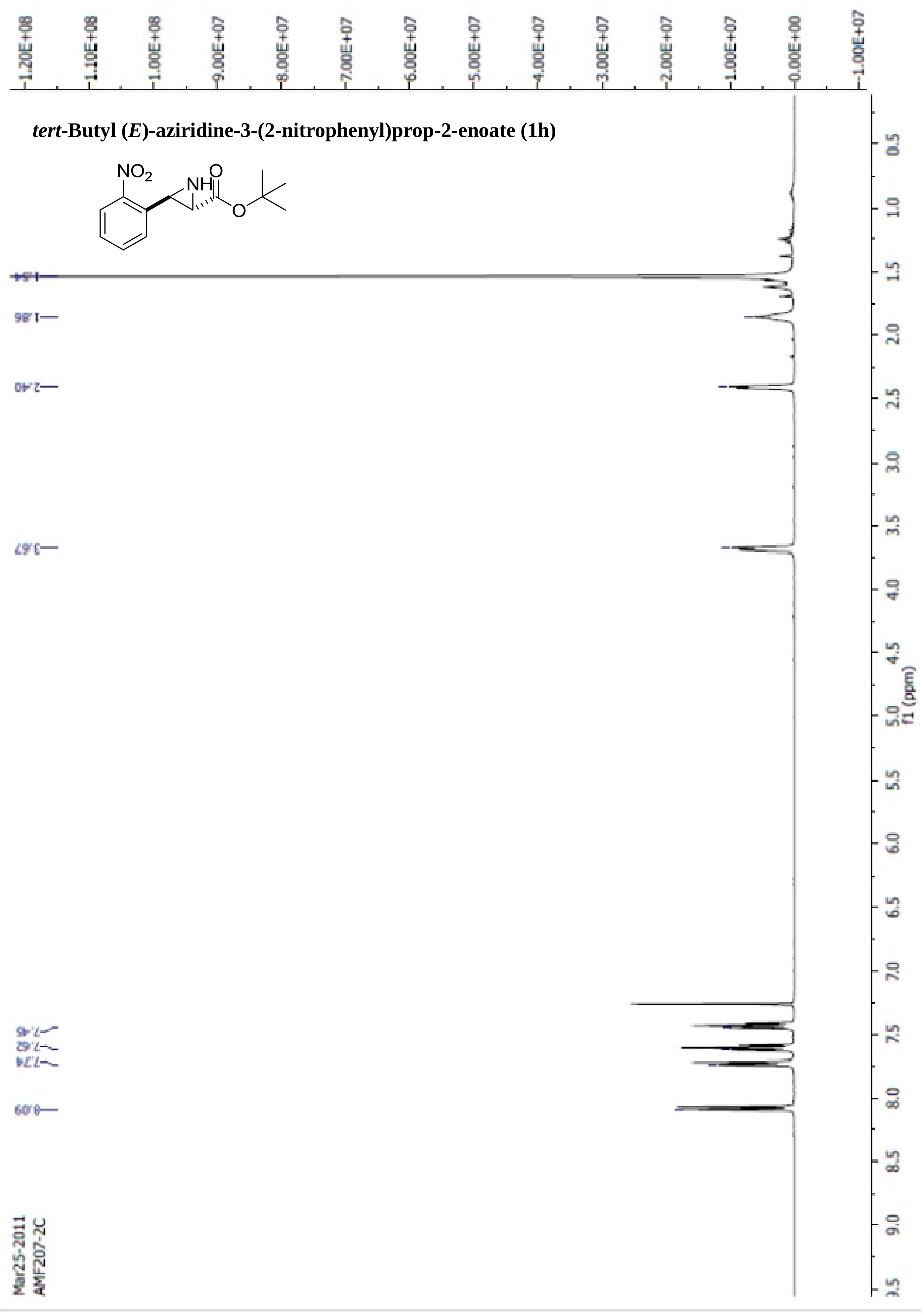

tert-Butyl (_E_)-aziridine-3-(2-nitrophenyl)prop-2-enoate (1h)

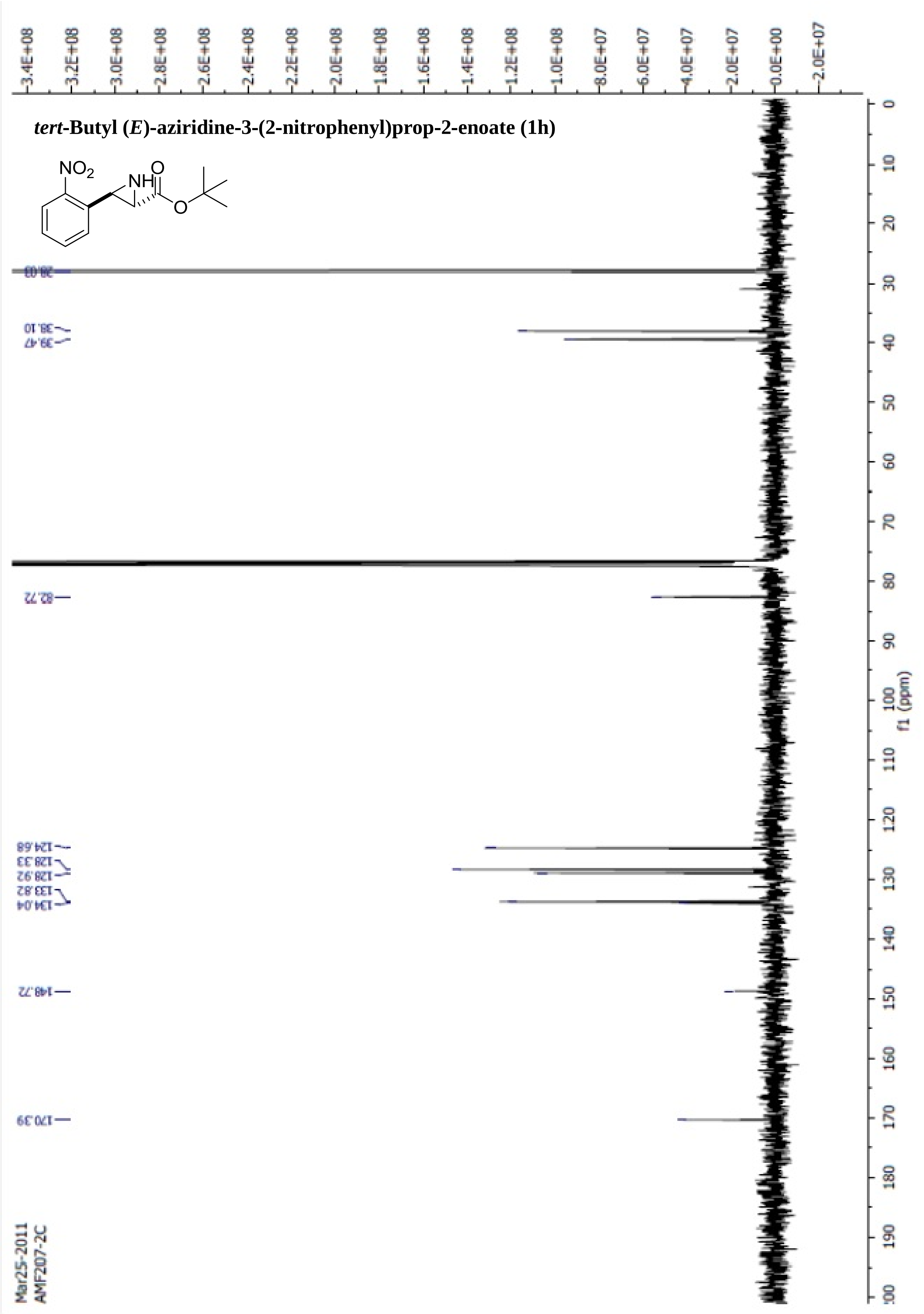

***tert*-Butyl (*E*)-aziridine-3-(5-chlorofuryl)prop-2-enoate (1j)**

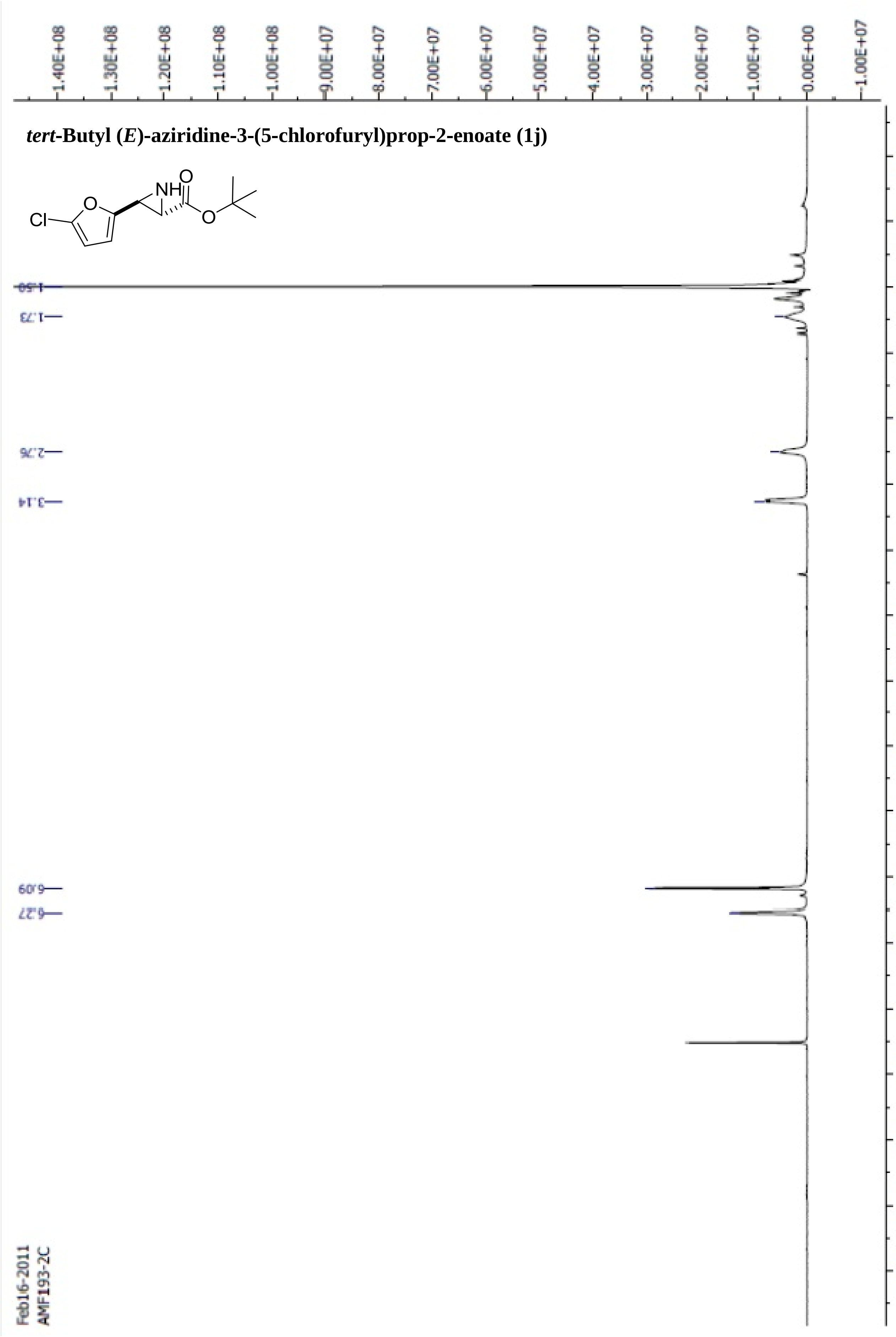

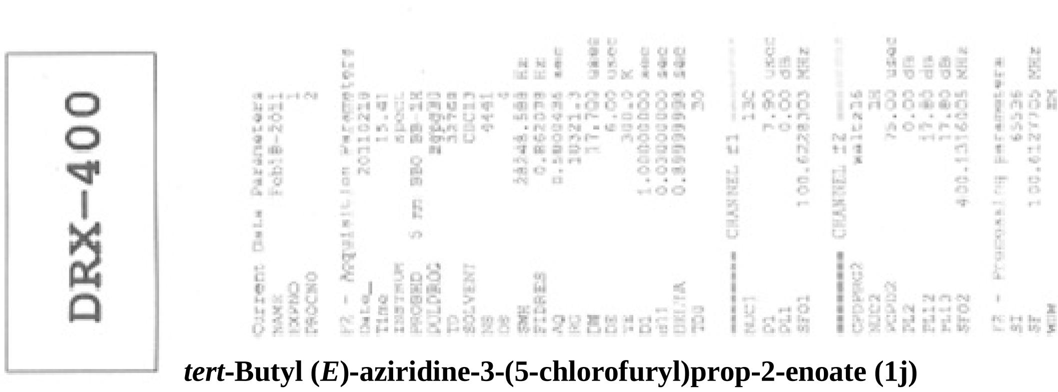

tert-Butyl (E)-aziridine-3-(5-chlorofuryl)prop-2-enoate (1j)

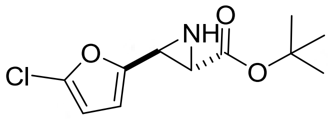

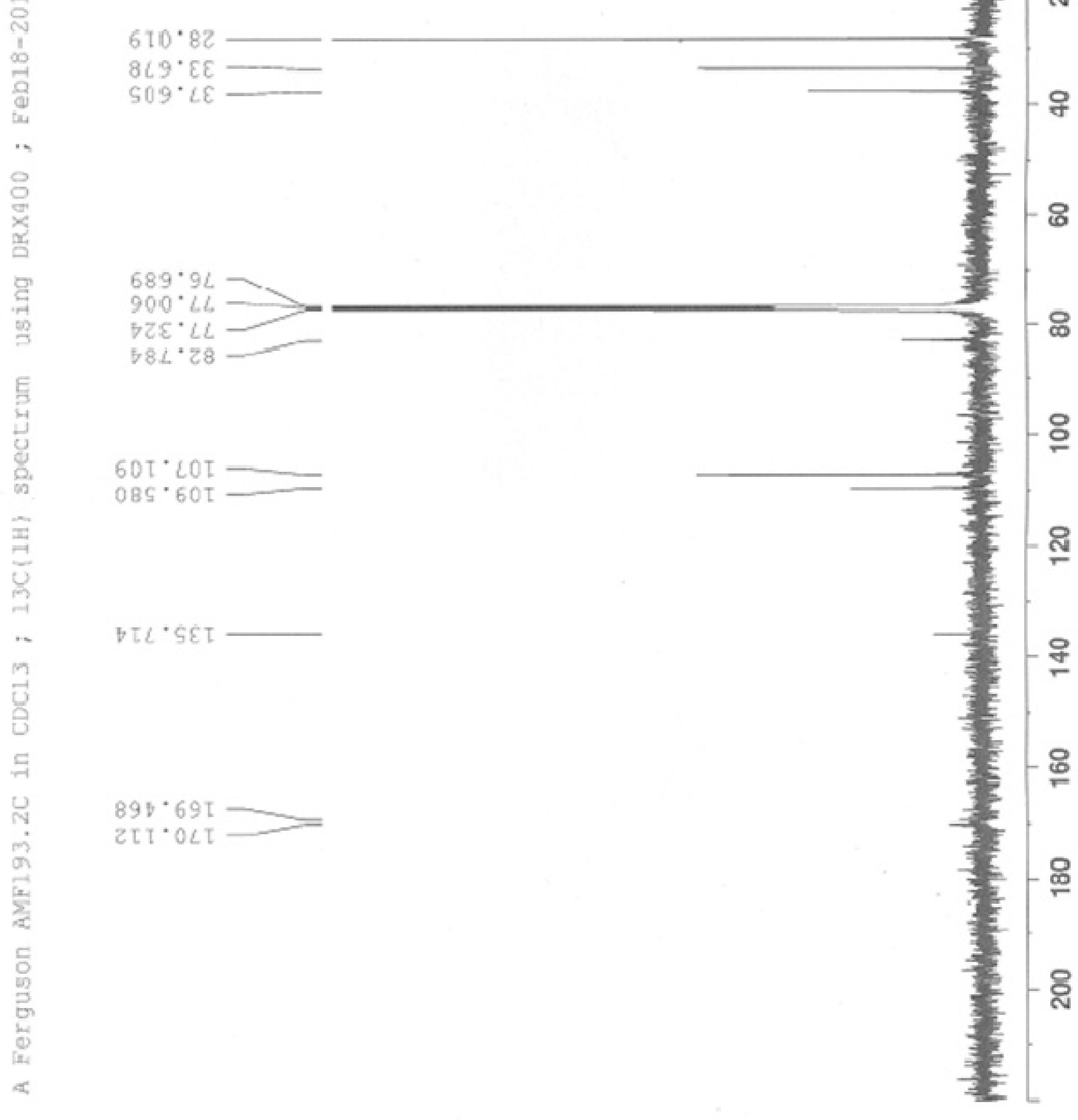

***tert*-Butyl (*E*)-aziridine-3-(5-chlorofuryl)prop-2-enoate (1j)**

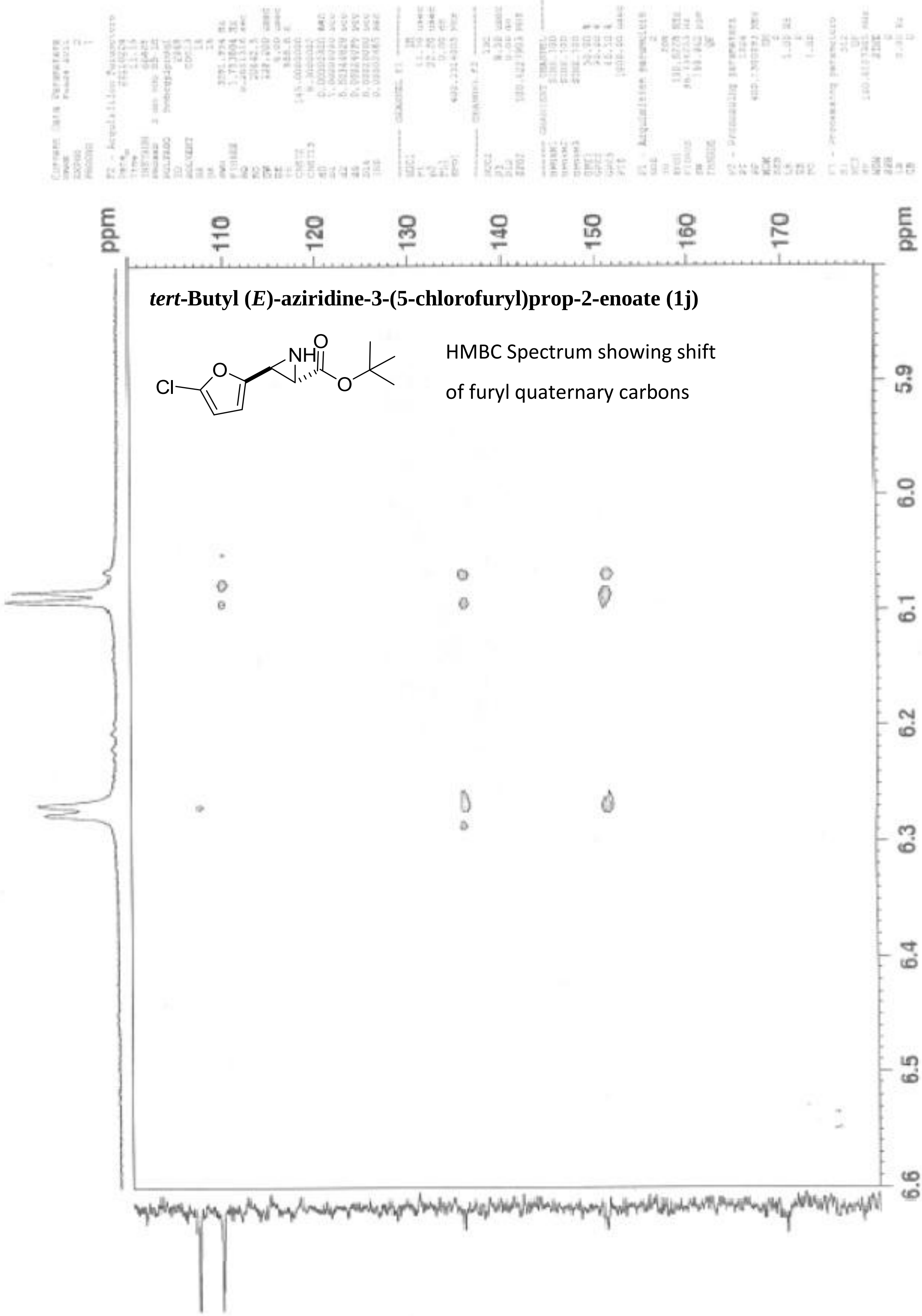

***tert*-Butyl 2-amino-3-phenyl-3-(indol-3yl)propanoate (2a)**

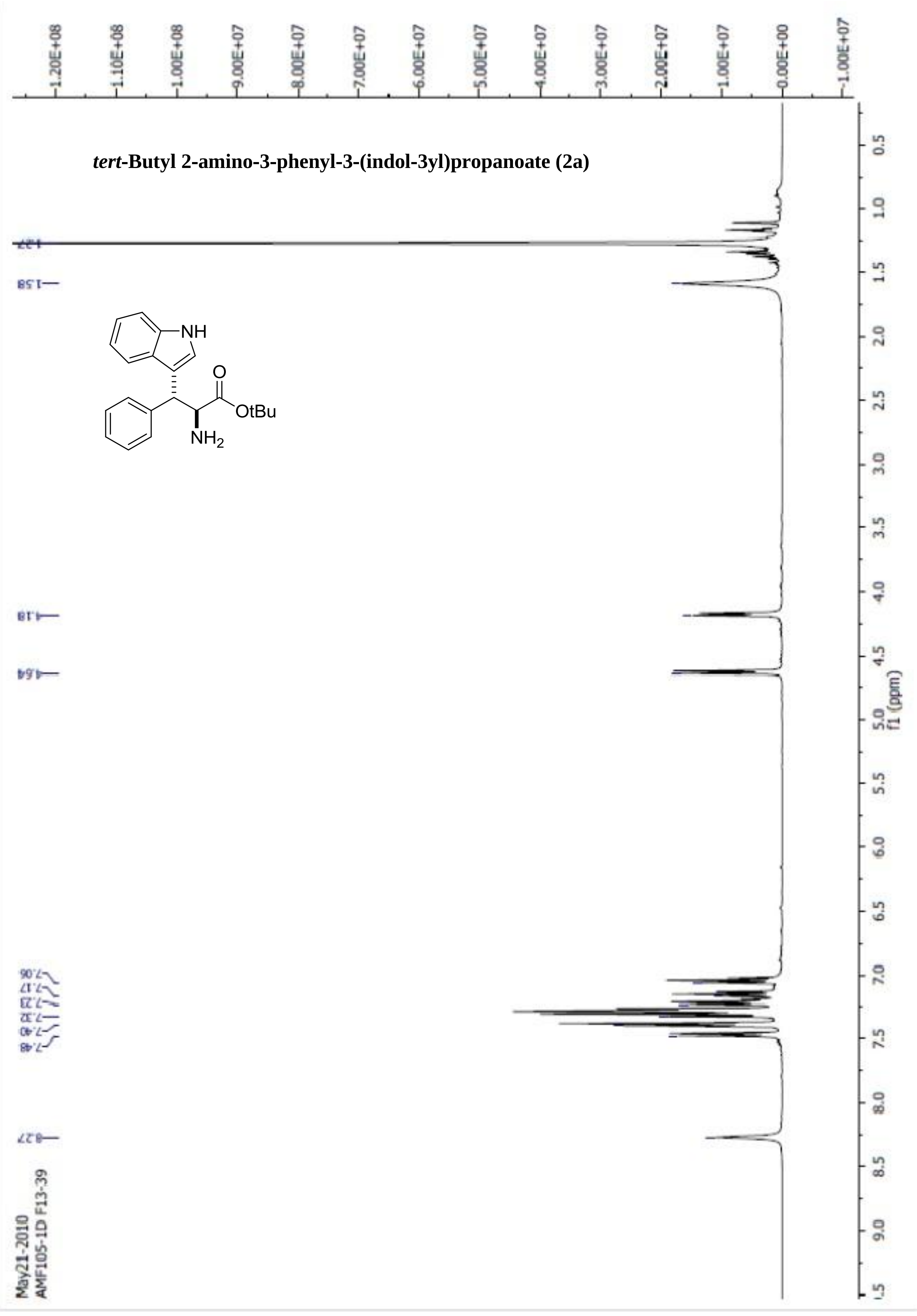

***tert*-Butyl 2-amino-3-phenyl-3-(indol-3yl)propanoate (2a)**

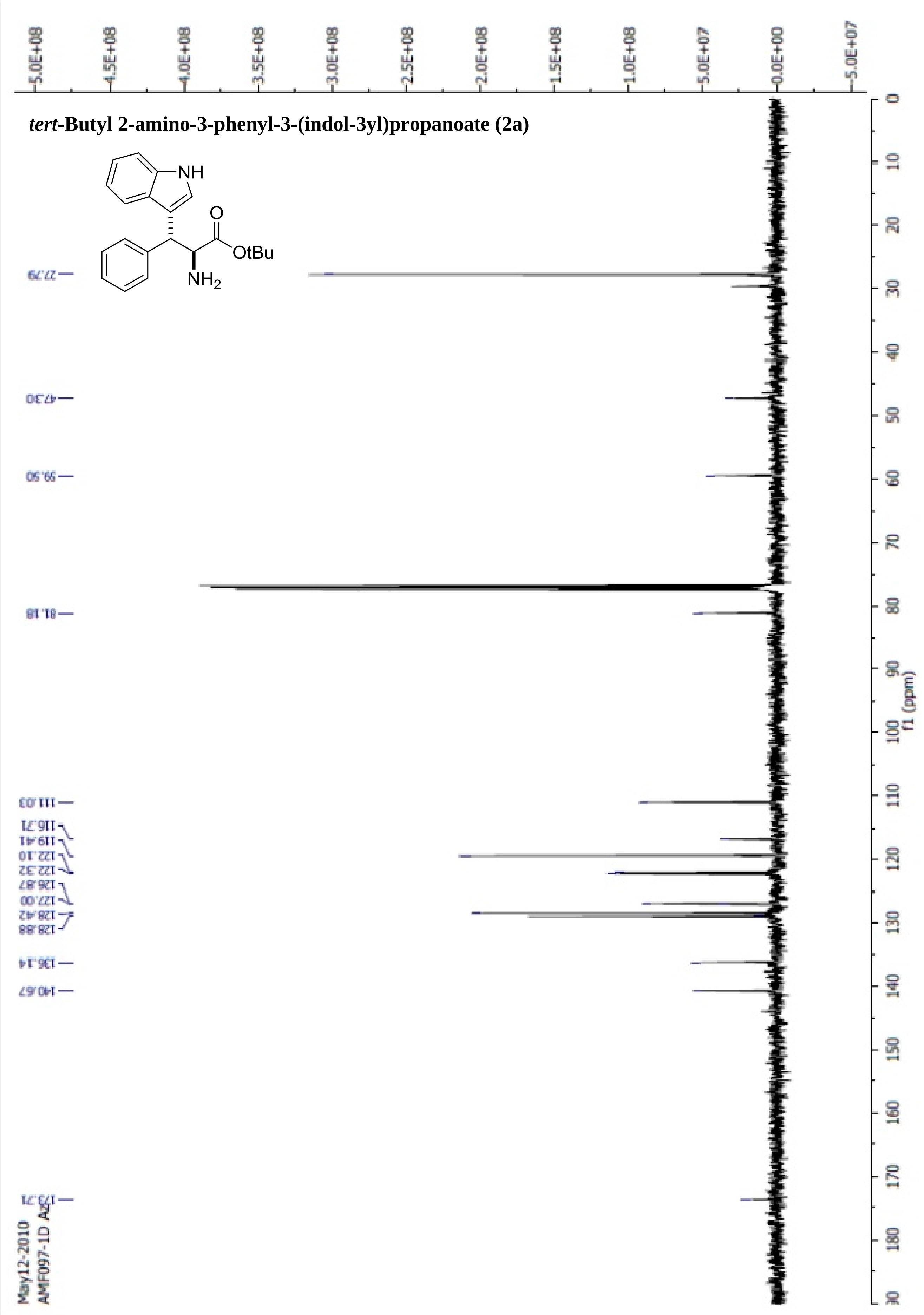

***tert*-Butyl 2-amino-3-phenyl-3-(hydroxybenzyl)propanoate (2e)**

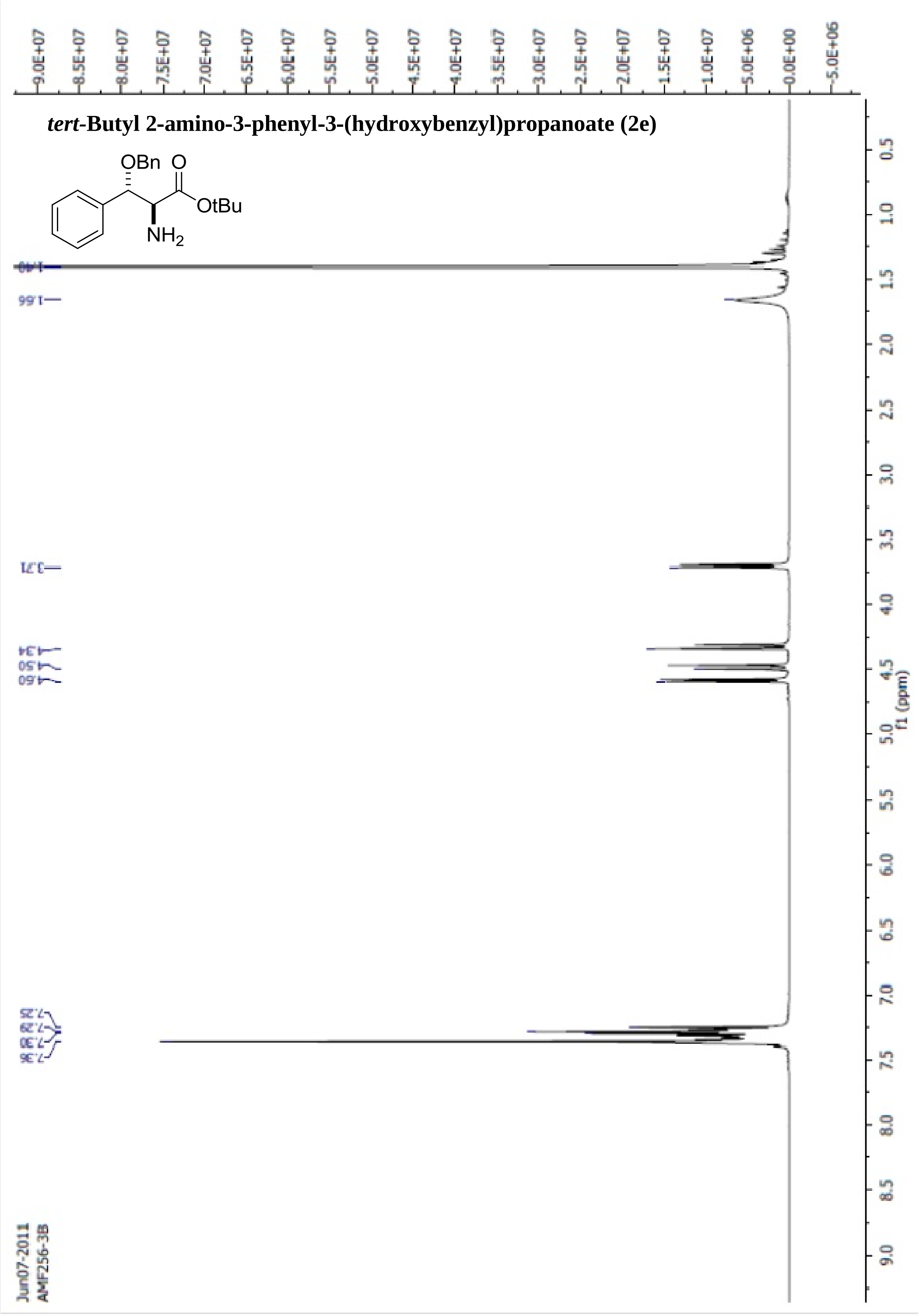

***tert*-Butyl 2-amino-3-phenyl-3-(hydroxybenzyl)propanoate (2e)**

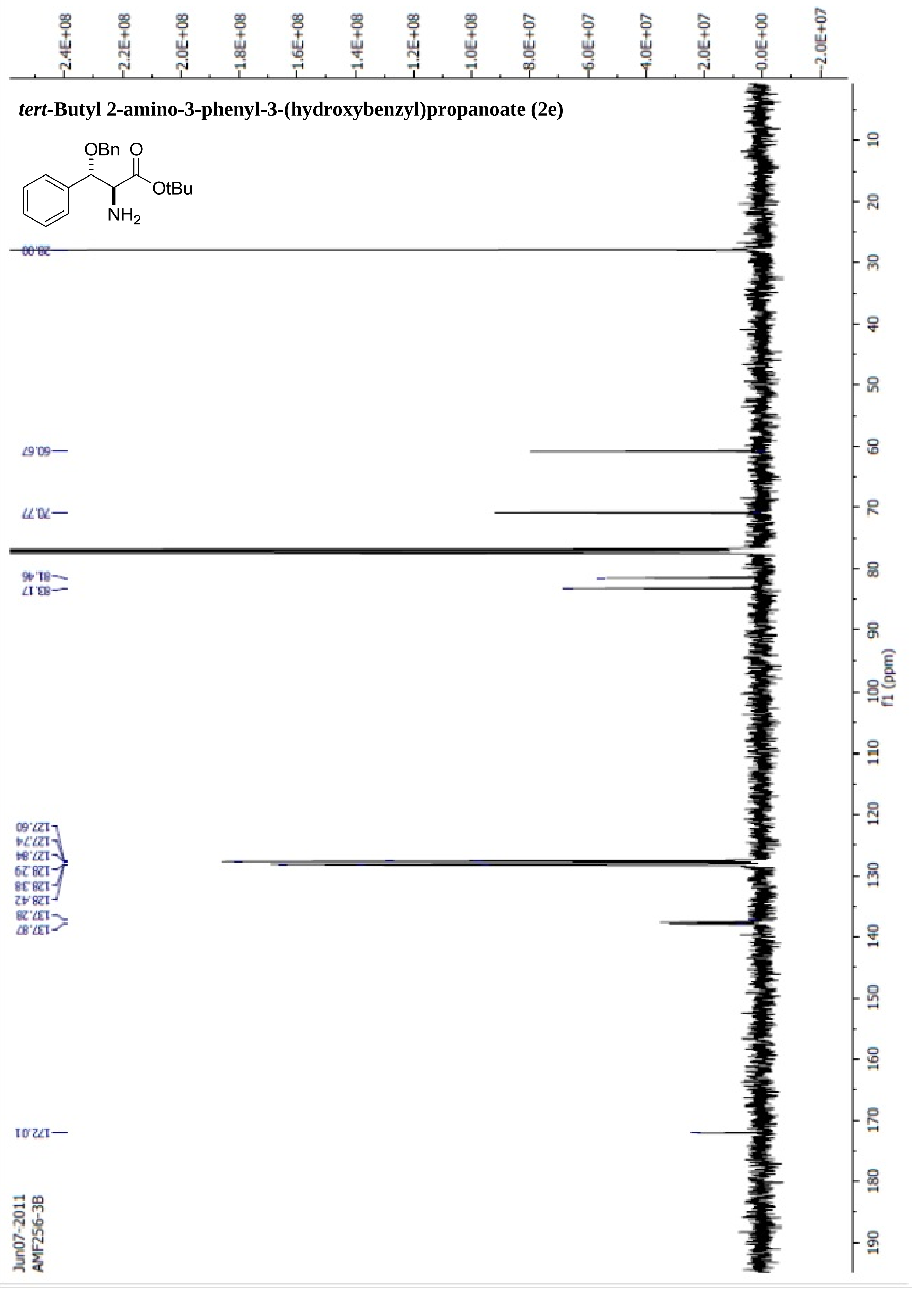

***tert*-Butyl 2-amino-3-phenyl-3-(hydroxyallyl)propanoate (2d)**

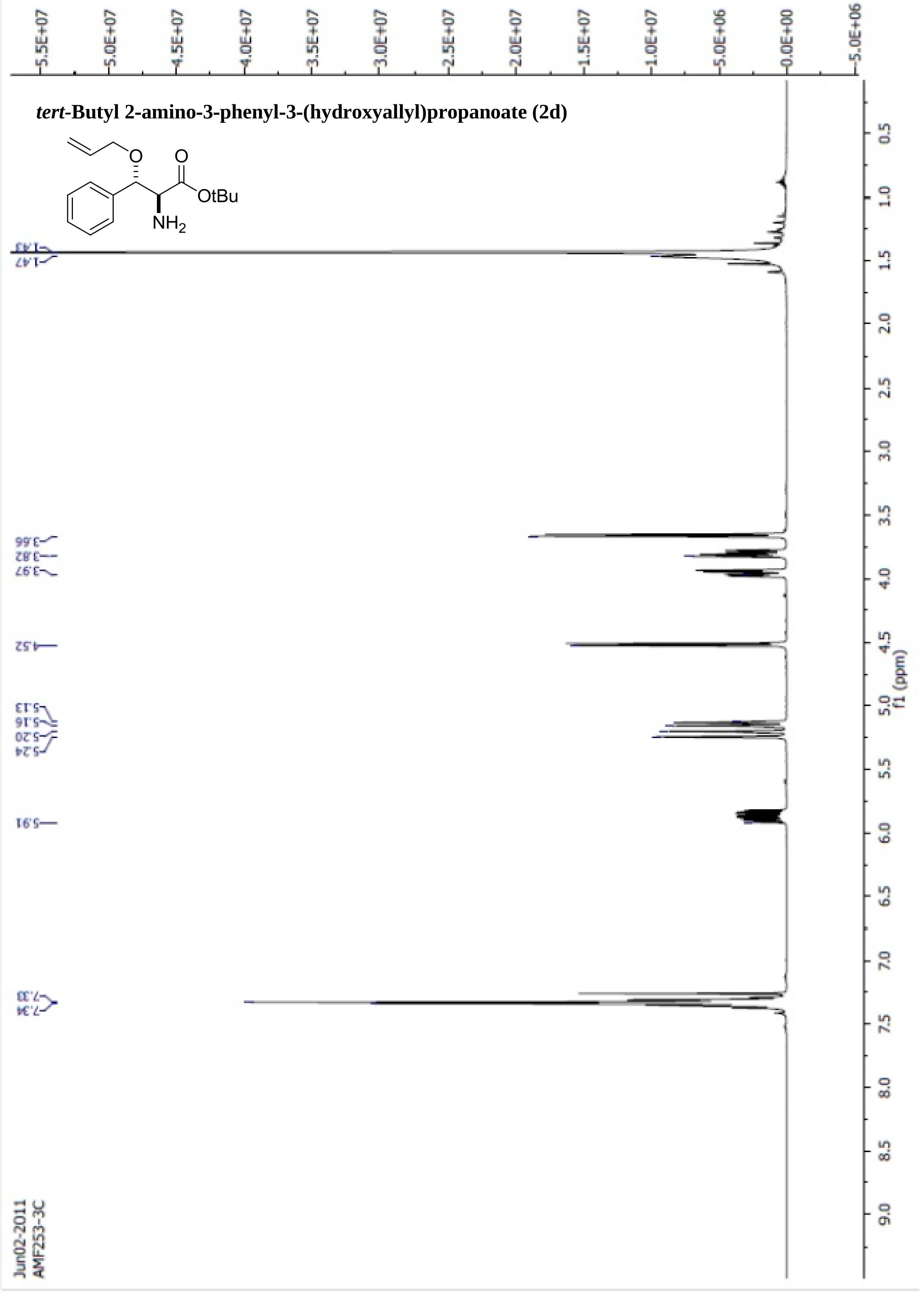

tert-Butyl 2-amino-3-phenyl-3-(hydroxyallyl)propanoate (2d)

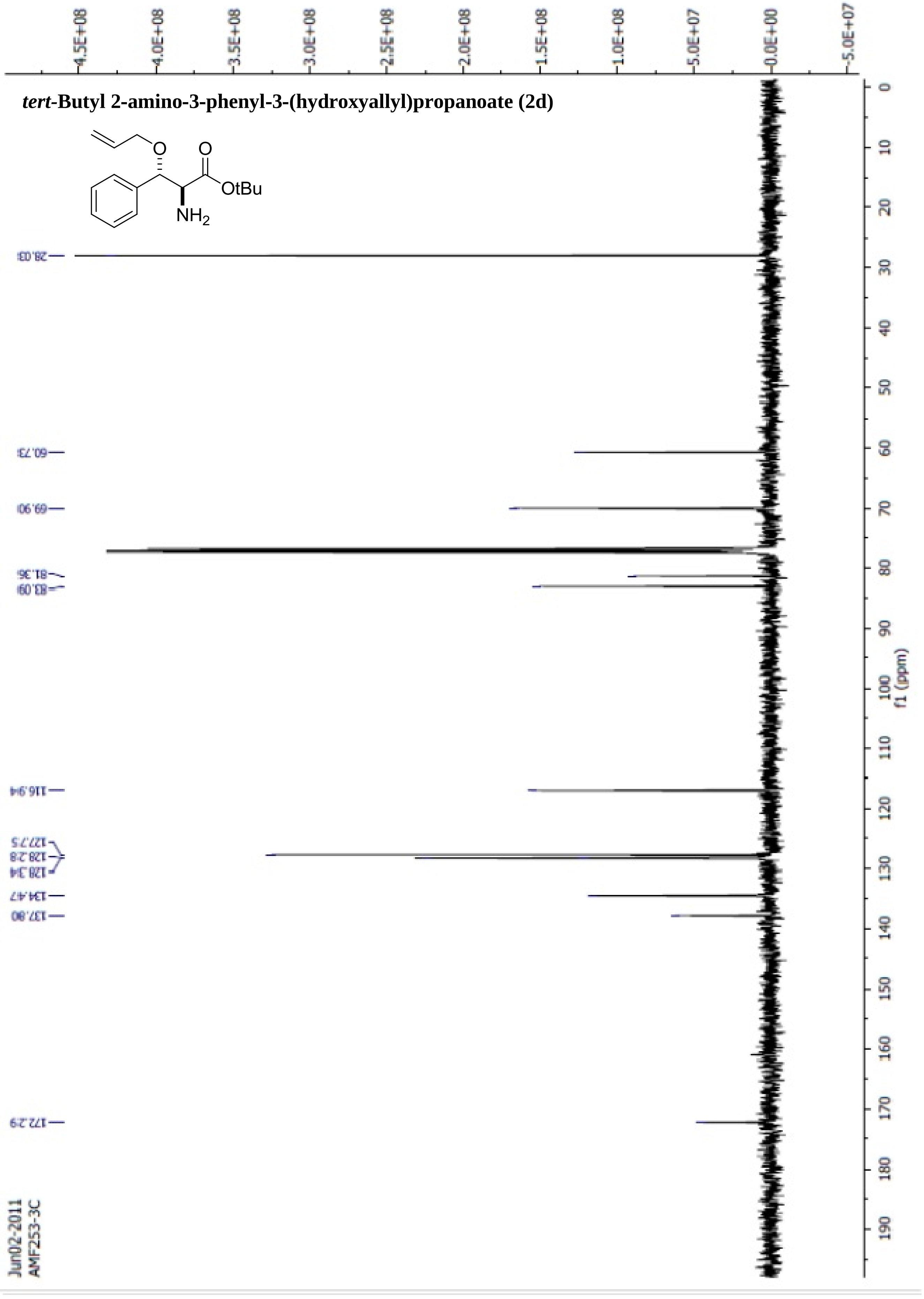

tert-Butyl 2-amino-3-phenyl-3-(methoxy)propanoate (2c)

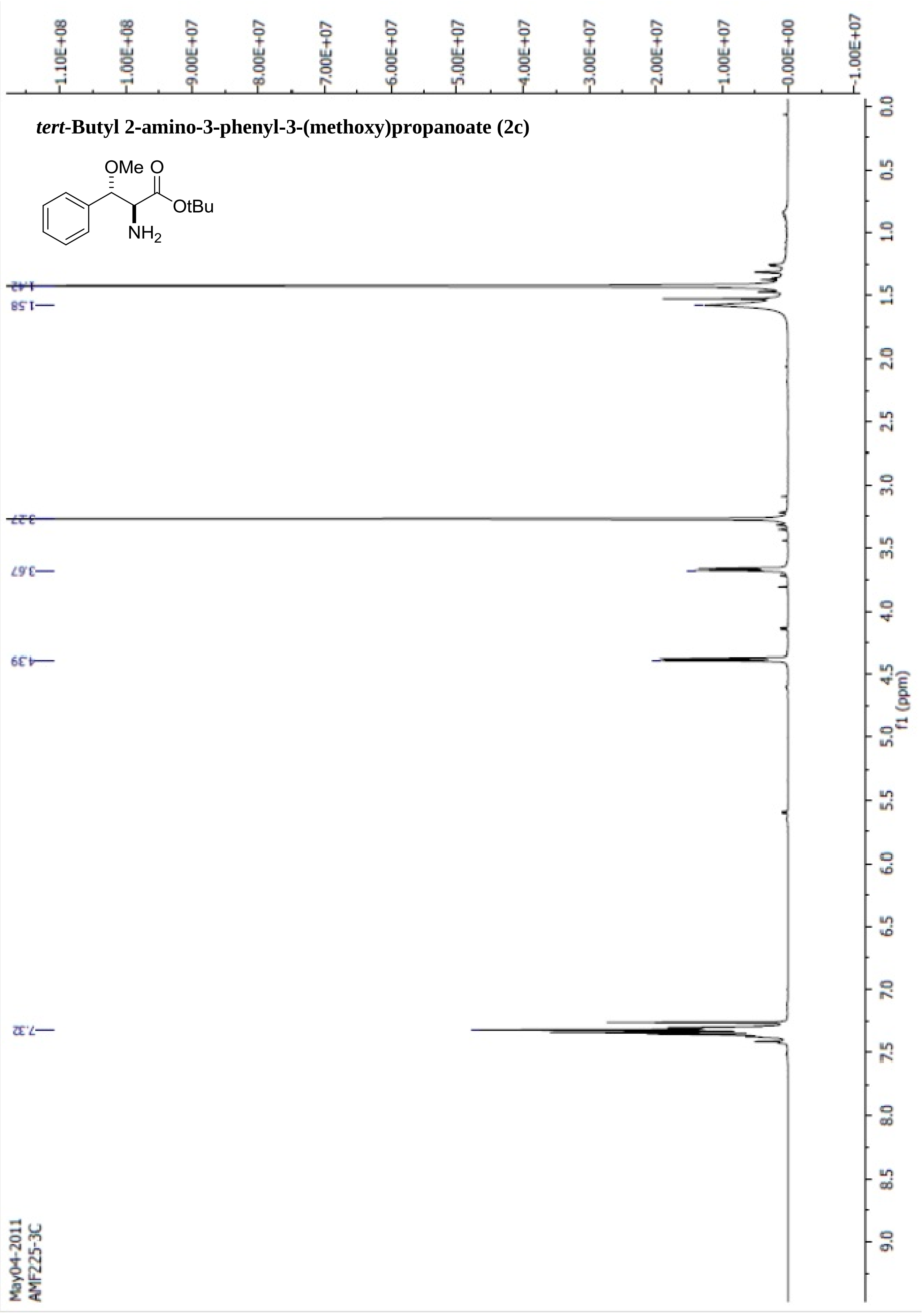

***tert*-Butyl 2-amino-3-phenyl-3-(methoxy)propanoate (2c)**

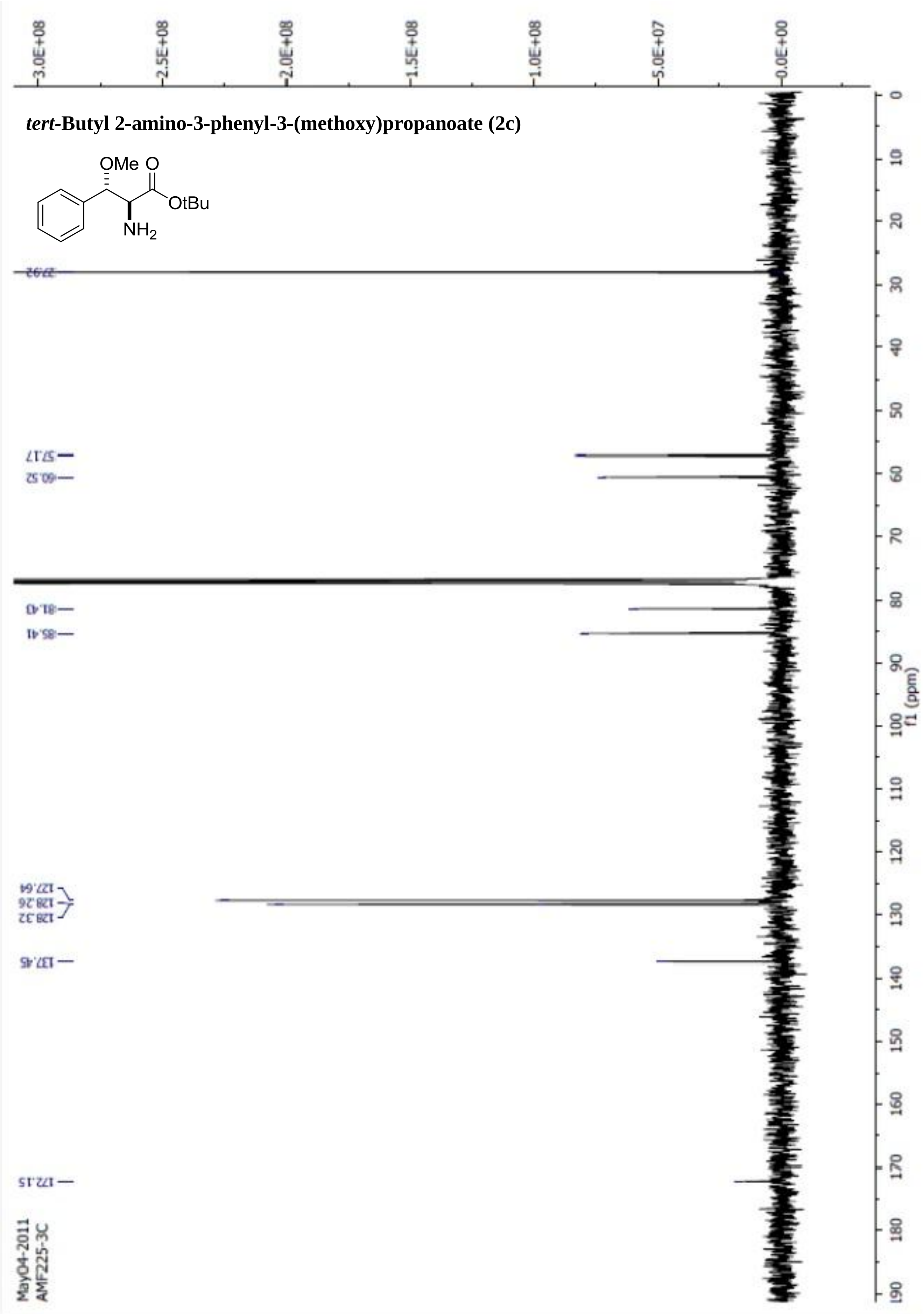

tert-Butyl 2-acetylamino-3-phenyl-3-(hydroxyl)propanoate (2f)

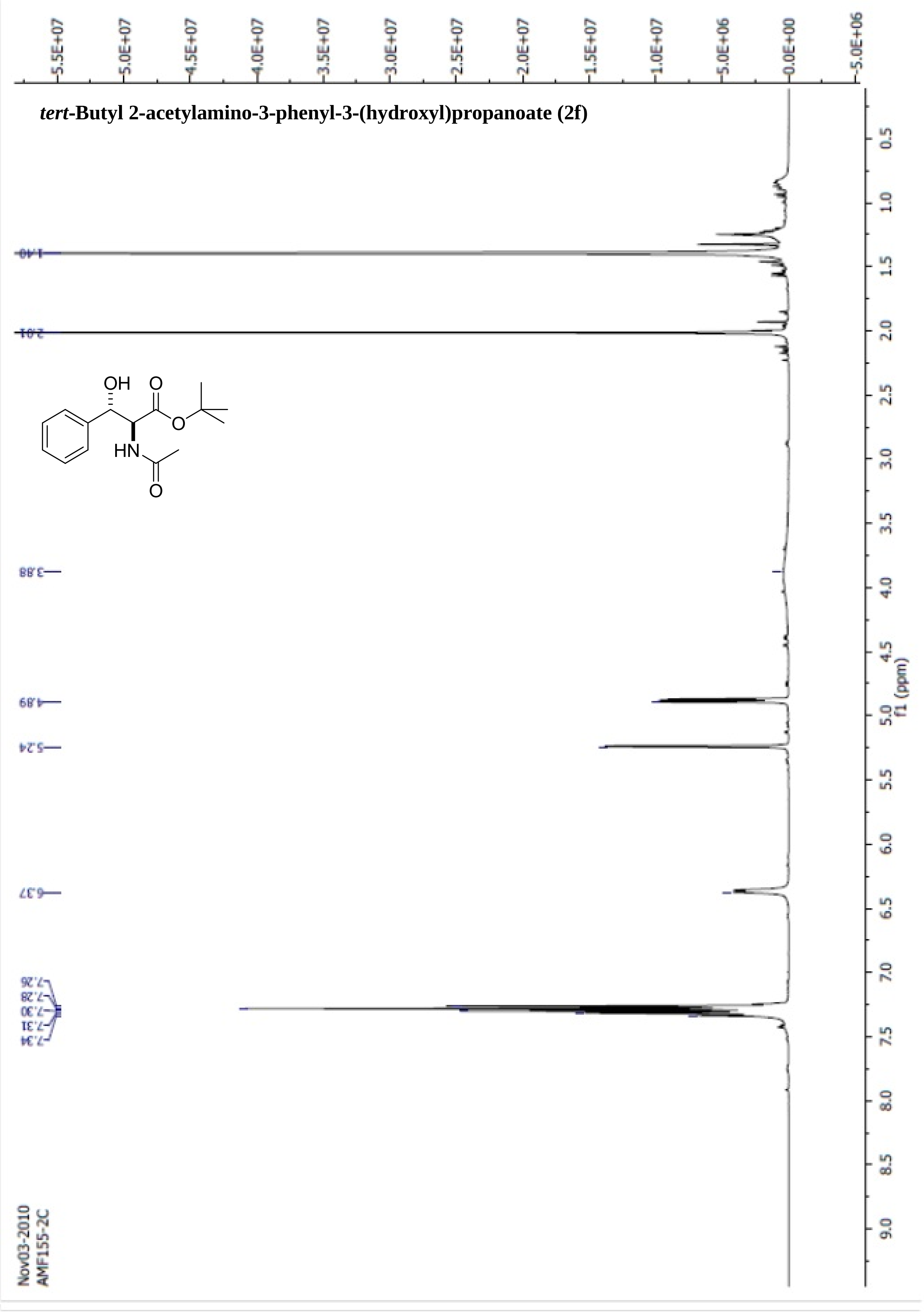

***tert*-Butyl 2-acetylamino-3-phenyl-3-(hydroxyl)propanoate (2f)**

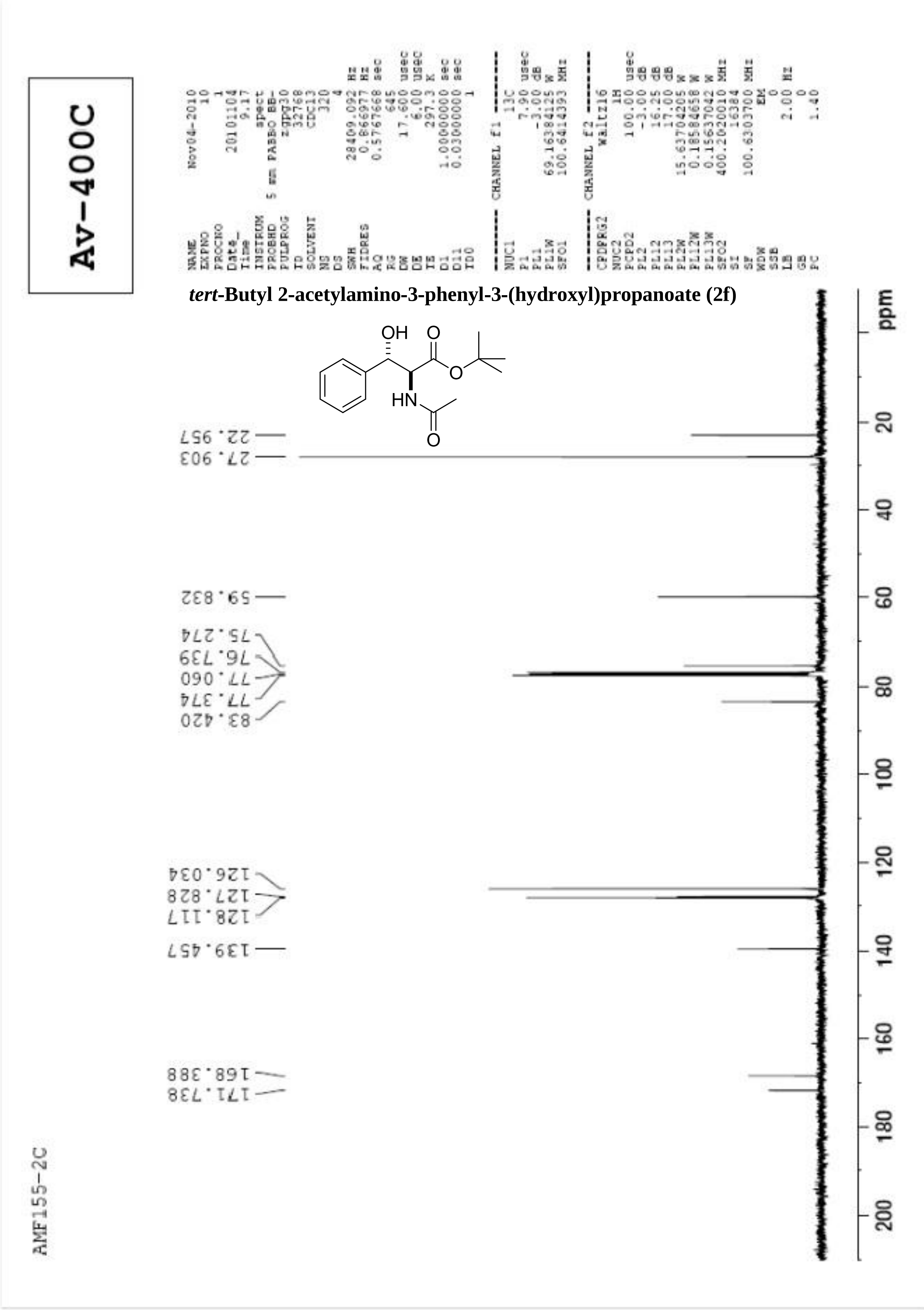

3-Chloro-3-phenylalanine *tert*-butyl ester (2i)

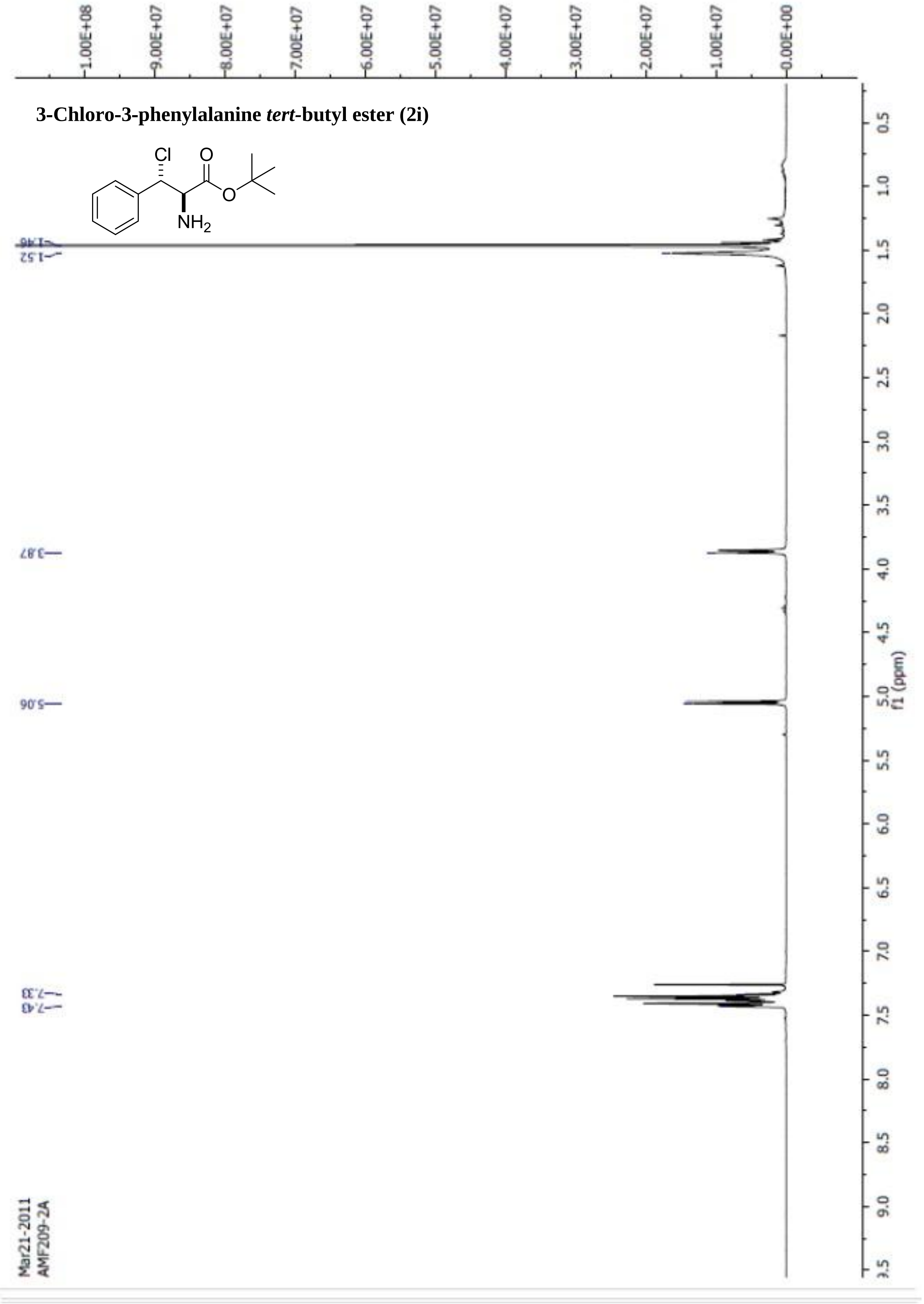

3-Chloro-3-phenylalanine *tert*-butyl ester (2i)

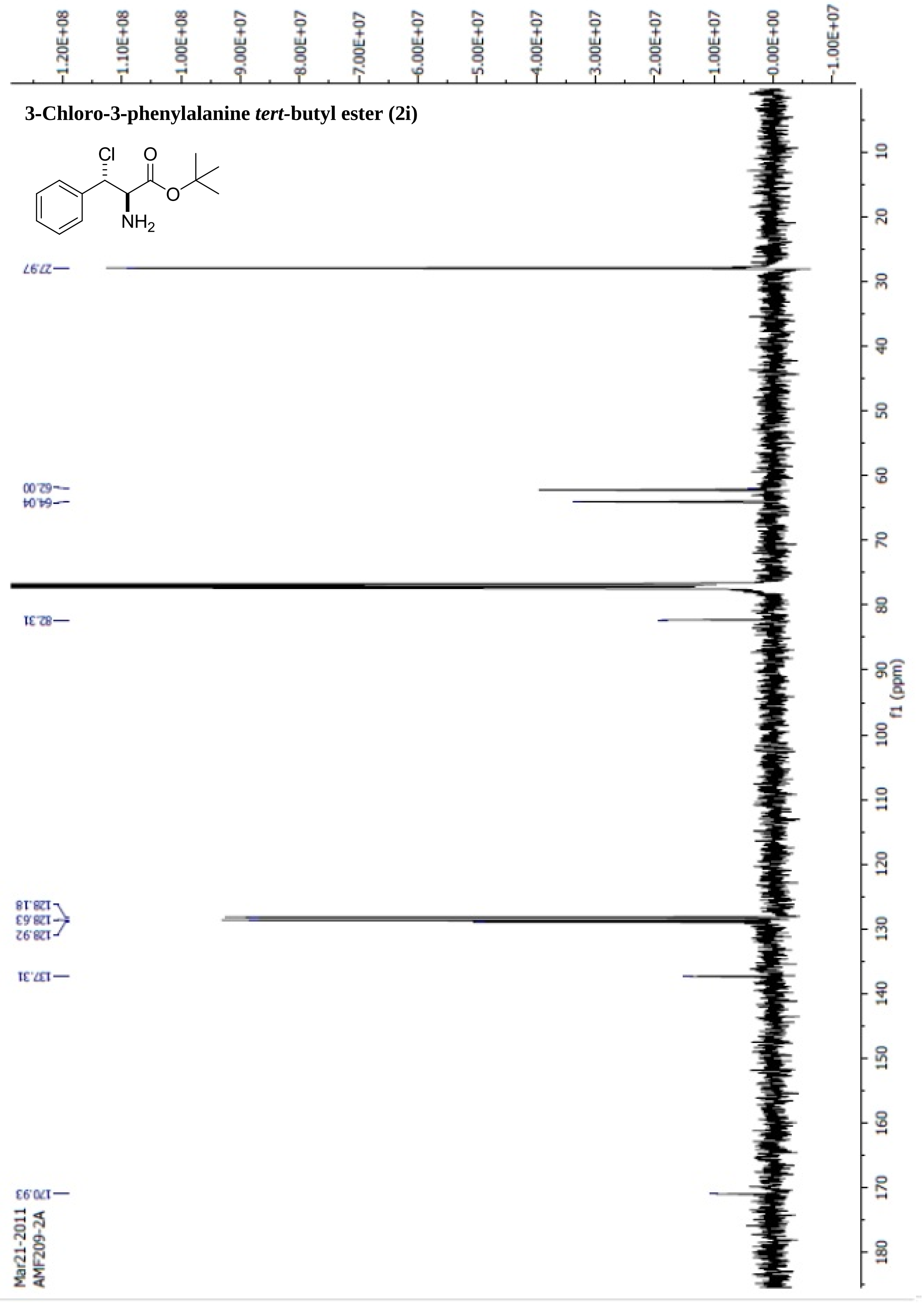

3-Chloro-3-phenylalanine *tert*-butyl ester hydrochloride (2h)

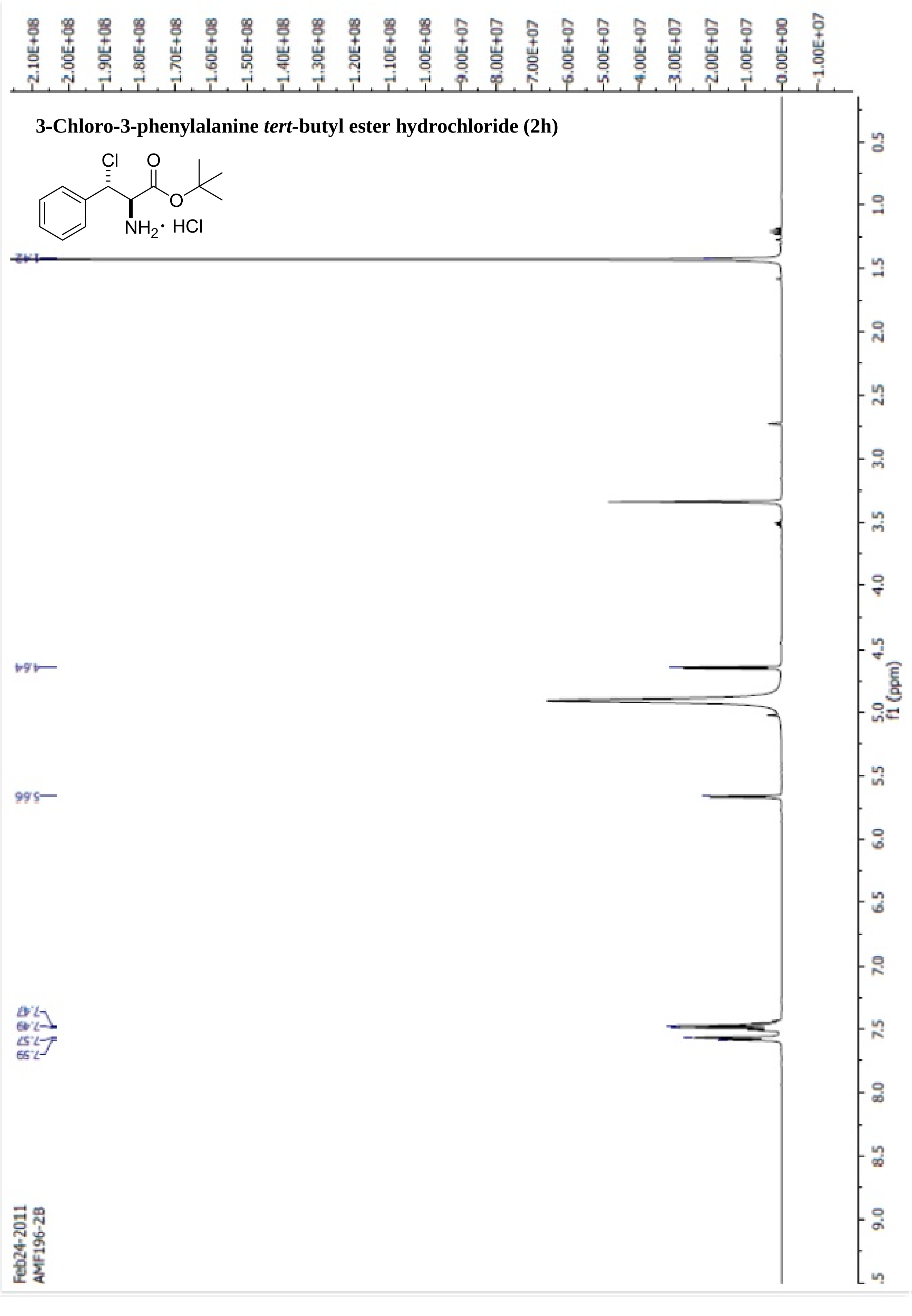

3-Chloro-3-phenylalanine *tert*-butyl ester hydrochloride (2h)

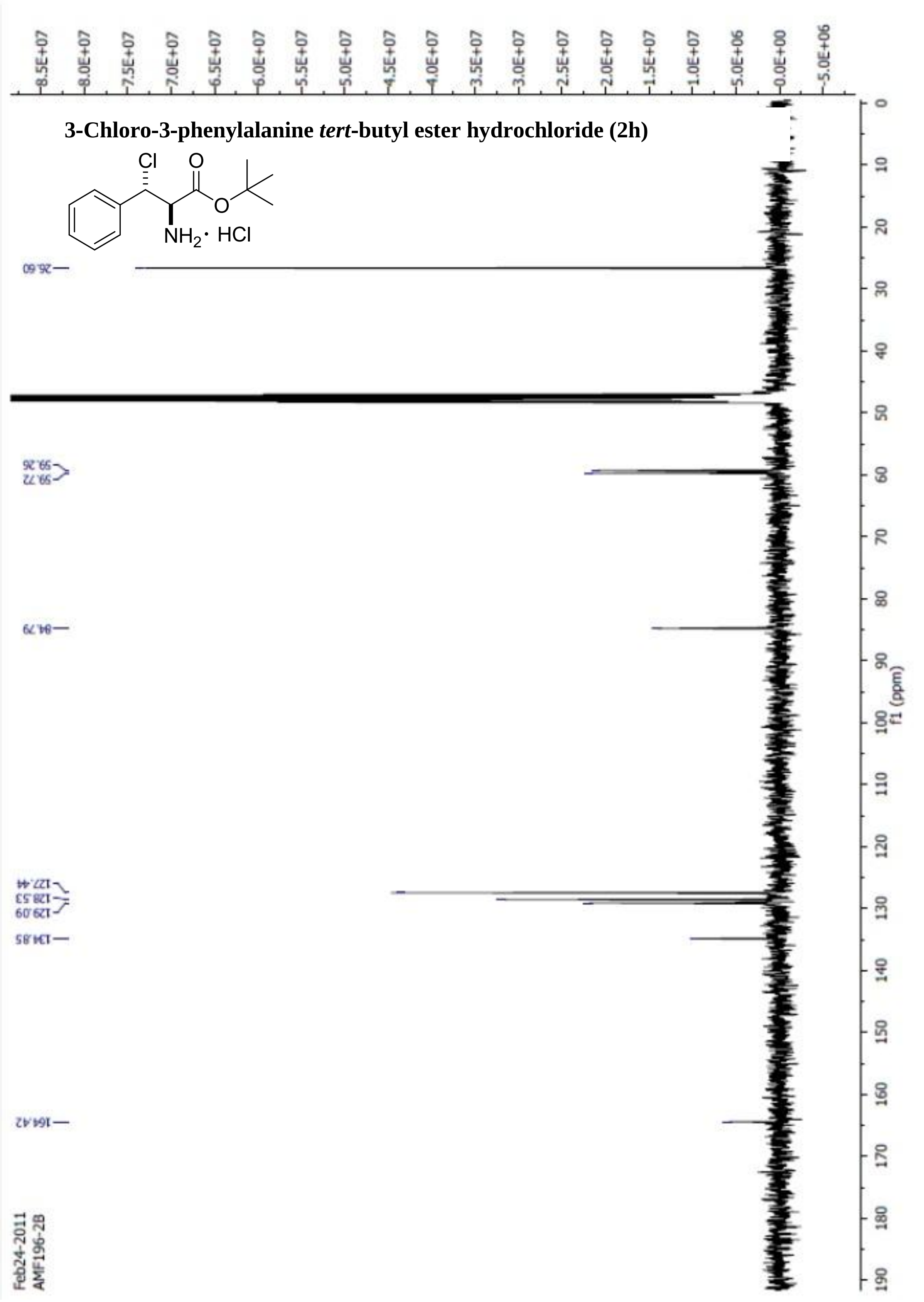

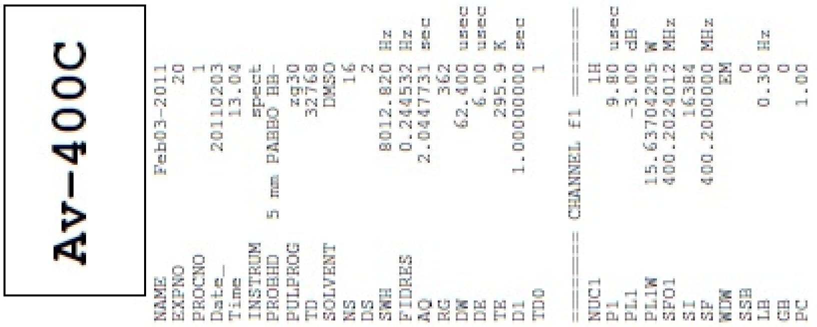

3-Chloro-3-phenylalanine hydrochloride (2g)

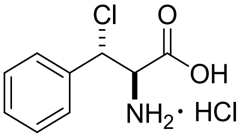

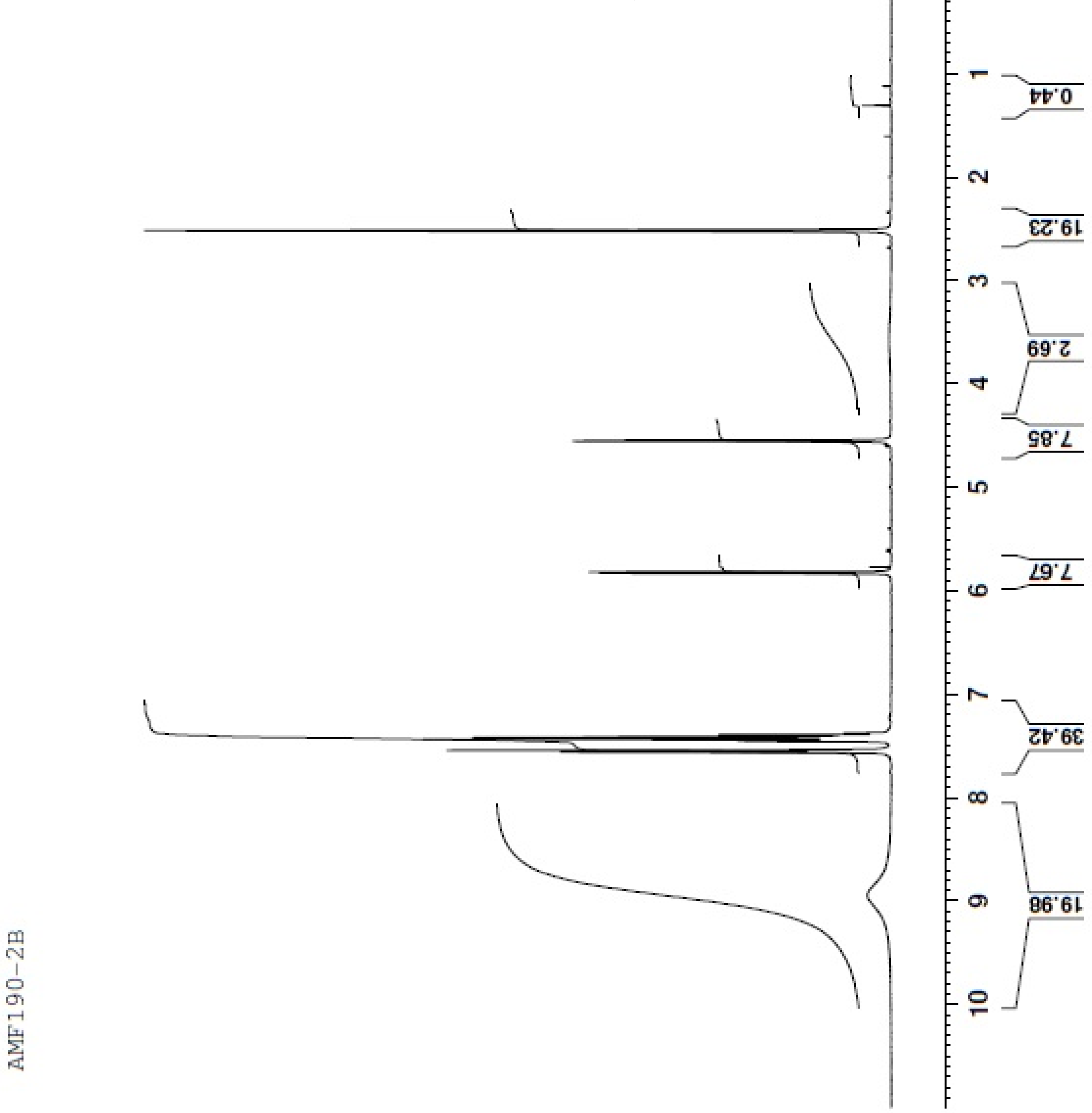

3-Chloro-3-phenylalanine hydrochloride (2g)

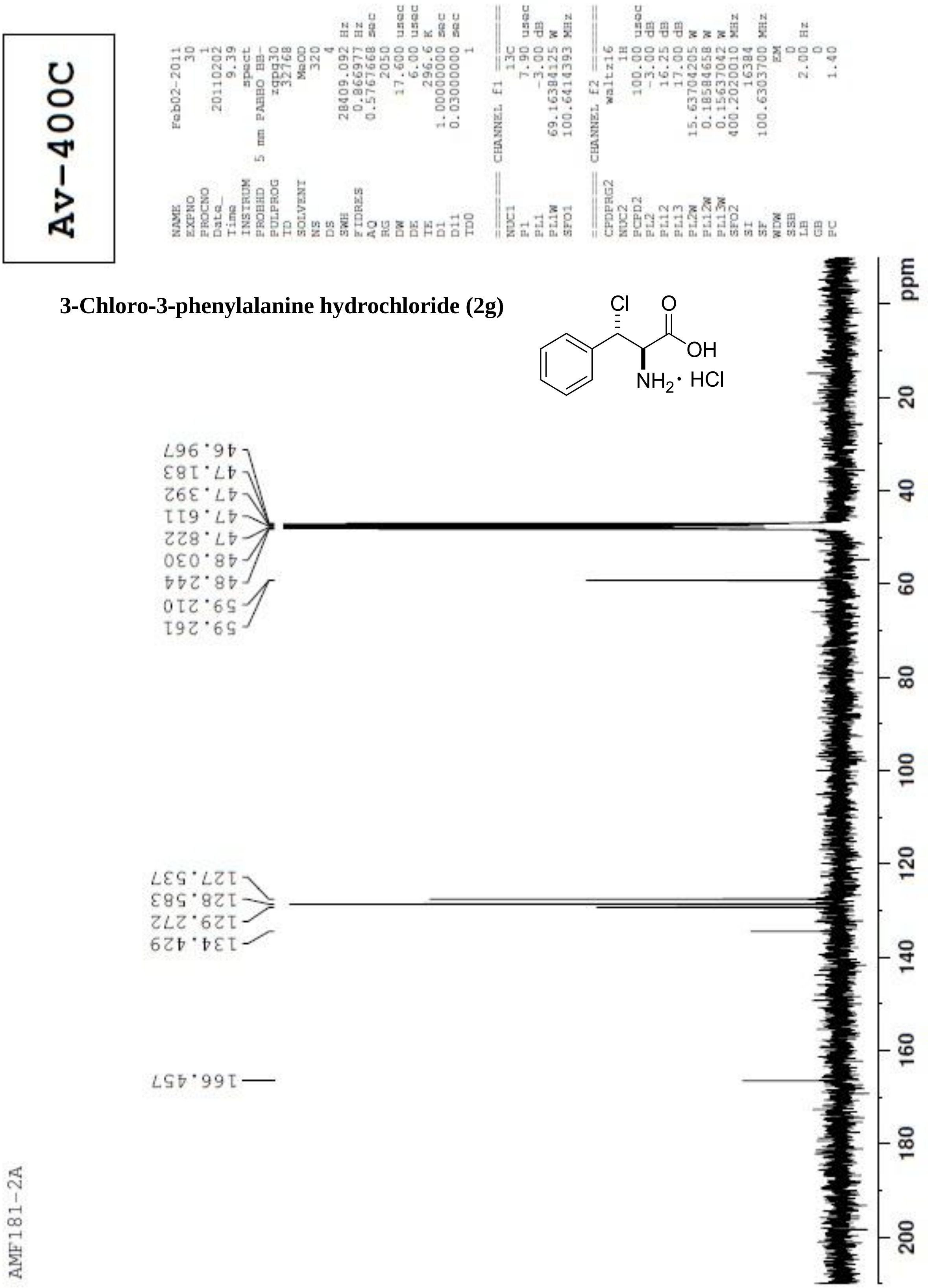

X-Ray Crystallography

The X-ray crystal structure of 1a

Crystal data for 1a: $C_{13}H_{17}NO_2$, M = 219.28, monoclinic, $P2_1/n$ (no. 14), a = 5.7745(3), b = 20.4714(8), c = 10.5178(4) Å, β = 98.175(4)°, V = 1230.70(9) Å^3, Z = 4, D_c = 1.183 g cm^{-3}, μ(Mo-Kα) = 0.080 mm^{-1}, T = 173 K, colourless thin plates, Oxford Diffraction Xcalibur 3 diffractometer; 2454 independent measured reflections (R_{int} = 0.0295), F^2 refinement, [10] R_1(obs) = 0.0487, wR_2(all) = 0.1092, 1619 independent observed absorption-corrected reflections [$|F_o| > 4\sigma(|F_o|)$, $2\theta_{max}$ = 56°], 167 parameters. CCDC 884567.

The N–H proton was located from a ΔF map and refined freely subject to an N–H distance constraint of 0.90 Å. The O(11)-bound tert-butyl group was found to be disordered, and two orientations of ca. 84 and 16% occupancy were identified. The geometries of the two orientations were optimised, the thermal parameters of adjacent atoms were restrained to be similar, and only the non-hydrogen atoms of the major occupancy orientation were refined anisotropically (the remainder were refined isotropically).

Figures

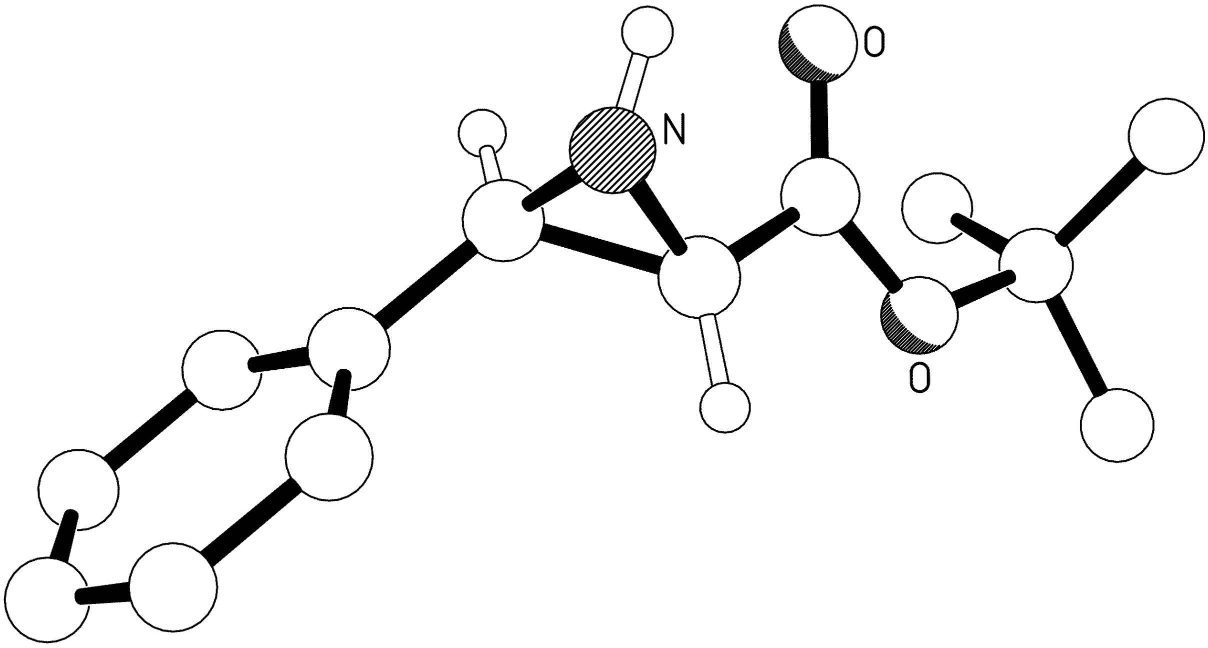

Figure S1: The crystal structure of **1a**.

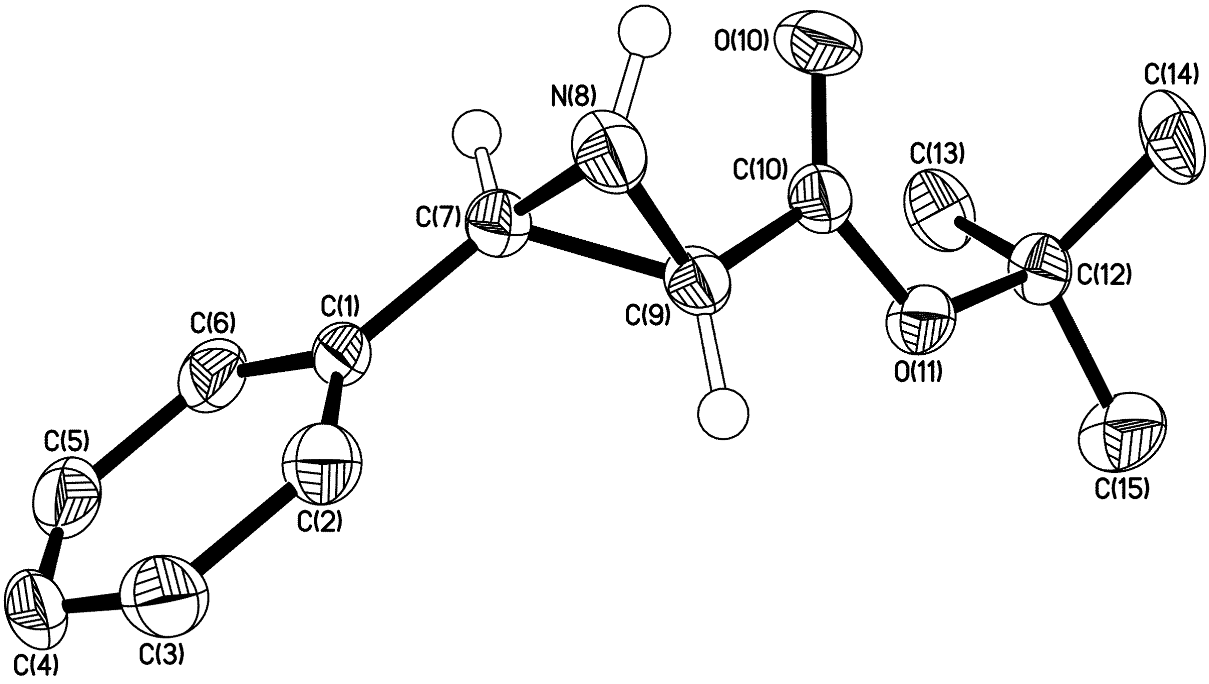

Figure S2: The crystal structure of **1a** (50% probability ellipsoids).

References

1. Claridge, T. D. W.; Davies, S. G.; Lee, J. A.; Nicholson, R. L.; Roberts, P. M.; Russell, A. J.; Smith, A. D.; Toms, S. M., *Org. Lett.* **2008,** 10, 5437–5440.
2. Gillespie, K. M.; Sanders, C. J.; O'Shaughnessy, P.; Westmoreland, I.; Thickitt, C. P.; Scott, P., *J. Org. Chem.* **2002,** 67, 3450–3458.
3. Davies, S. G.; Mulvaney, A. W.; Russell, A. J.; Smith, A. D., *Tetrahedron Asymm.* **2007,** 18, 1554–1566.
4. Paquin, J.-F.; Stephenson, C. R. J.; Defieber, C.; Carreira, E. M., *Org. Lett.* **2005,** 7, 3821–3824.
5. Bull, S. D.; Davies, S. G.; Delgado-Ballester, S.; Kelly, P. M.; Kotchie, L. J.; Gianotti, M.; Laderas, M.; Smith, A. D., *J. Chem. Soc. Perkin Trans. 1* **2001,** 23, 3112–3121.
6. Li, K.; Ran, L.; Yu, Y.-H.; Tang, Y., *J. Org. Chem.* **2004,** 69, 3986–3989.
7. Armstrong, A.; Baxter, C. A.; Lamont, S. G.; Pape, A. R.; Wincewicz, R., *Org. Lett.* **2007,** *9*, 351–353.
8. Nemes, C.; Jeannin, L.; Sapi, J.; Laronze, M.; Seghir, H.; Auge, F.; Laronze, J., *Tetrahedron* **2000,** *56*, 5479.
9. D'Arrigo, P.; Kanerva, L. T.; Li, X.-G.; Saraceno, C.; Servi, S.; Tessaro, D., *Tetrahedron Asymm.* **2009,** *20*, 1641–1645.
10. Sheldrick, G. M. *Acta Cryst.*, **2008,** A64, 112–122.
